# STING Promotes the Progression of ADPKD by Regulating Mitochondrial Function, Inflammation, Fibrosis, and Apoptosis

**DOI:** 10.3390/biom14101215

**Published:** 2024-09-26

**Authors:** Jiao Wu, Shasha Cheng, Geoffray Lee, Ewud Agborbesong, Xiaoyan Li, Xia Zhou, Xiaogang Li

**Affiliations:** 1 Department of Internal Medicine, Mayo Clinic, Rochester, MN 55905, USA; wujiao@ctgu.edu.cn (J.W.); cheng.shasha@mayo.edu (S.C.); geoffraylee07@gmail.com (G.L.); agborbesong.ewud@mayo.edu (E.A.); li.xiaoyan@mayo.edu (X.L.); zhou.xia@mayo.edu (X.Z.); 2 Department of Biochemistry and Molecular Biology, Mayo Clinic, Rochester, MN 55905, USA

**Keywords:** ADPKD, STING, mitochondrial

## Abstract

Autosomal dominant polycystic kidney disease (ADPKD) is a predominant genetic disease, which is caused by mutations in PKD genes and is associated with DNA damage in cystic cells. The intrinsic stimulator of interferon genes (STING) pathway is crucial for recognizing damaged DNA in the cytosol, triggering the expression of inflammatory cytokines to activate defense mechanisms. However, the precise roles and mechanisms of STING in ADPKD remain elusive. In this study, we show that *Pkd1* mutant mouse kidneys show upregulation of STING, which is stimulated by the DNAs of nuclear and mitochondrial origin. The activation of STING promotes cyst growth through increasing (1) the activation of NF-κB in *Pkd1* mutant cells and (2) the recruitment of macrophages in the interstitial and peri-cystic regions in *Pkd1* mutant mouse kidneys via NF-κB mediating the upregulation of TNF-α and MCP-1. Targeting STING with its specific inhibitor C-176 delays cyst growth in an early-stage aggressive *Pkd1* conditional knockout mouse model and a milder long-lasting *Pkd1* mutant mouse model. Targeting STING normalizes mitochondrial structure and function, decreases the formation of micronuclei, induces *Pkd1* mutant renal epithelial cell death via p53 signaling, and decreases renal fibrosis in *Pkd1* mutant mouse kidneys. These results support that STING is a novel therapeutic target for ADPKD treatment.

## 1. Introduction

Autosomal dominant polycystic kidney disease (ADPKD) is the most common genetic disease, which is caused by mutations in PKD1 and PKD2 genes, encoding for polycystin-1 and polycystin-2 proteins, respectively [1]. ADPKD is characterized by the formation and progressive growth of innumerous fluid-filled cysts in both kidneys [1]. Cyst growth and expansion in ADPKD ultimately destroys normal kidney tissue leading to end stage kidney disease (ESKD). ADPKD progression is highly variable and depends on the nature of the mutated gene. The “two-hit hypothesis” is a proposed theory to explain the kidney phenotype observed in ADPKD patients, in which an individual has an inherited germ line mutation (“first hit”), and the development of cysts starts only when another mutation (somatic mutations) occurs in either PKD1 or PKD2 (“second hit”) [2]. In the past two decades, diverse molecular mechanisms and numerous pathways have been identified that contribute to the regulation of cyst progression in ADPKD [3]. But to date, attempts to completely inhibit cystogenesis have not been successful. Thus, it is necessary to continue identifying novel mechanisms and pathways in promoting cystogenesis in ADPKD.

Chronic interstitial inflammation developed in ADPKD kidney results in cyst expansion and renal injury, even though PKD is not known to be primarily an inflammatory disorder [4,5]. The increase of tumor necrosis factor alpha (TNF-α) and subsequent signal transduction promote cyst growth in ADPKD mouse models [4]. An increase of the recruitment of macrophages in cystic kidneys has been reported to promote cystogenesis and disease progression [5]. The microphage migration inhibitory factor (MIF) also regulates the recruitment of macrophages through TNF-α and monocyte chemoattractant protein-1 (MCP-1) and promotes cyst growth in *Pkd1* mutant mouse kidneys [6]. In addition, inflammatory mediators and cytokines released within the cystic microenvironment may induce DNA damage in renal epithelial cells, exacerbating tissue injury [7]. Disruption of DNA repair mechanisms has been implicated in ADPKD pathogenesis [8]. Defects in DNA repair pathways, such as those involving homologous recombination or non-homologous end joining, may render cells more susceptible to accumulating genetic mutations and genomic instability, resulting in exacerbating disease progression [9]. Growing evidence indicates that the intrinsic stimulator of interferon genes (STING) pathway is crucial for recognizing damaged DNA or DNA pathogens in the cytosol [10]. In various disorders, the constitutively active DNA damage response can promote cytoplasmic DNA accumulation, which may trigger the activation of the cyclic GMP–AMP synthase (cGAS)-STING pathway and then an immune response [11]. In addition, viral infection and invasion by some intracellular bacteria may also result in an increase of DNA to the cytosol to activate the cGAS-STING pathway [12]. A key role of the cGAS–STING pathway is to detect cytosolic DNA and induce an immune response [13]. STING is a signaling molecule located on the endoplasmic reticulum (ER) and plays a crucial role in regulating the transcription of various host defense genes, including type I interferons (IFNs) and pro-inflammatory cytokines [14]. This regulation occurs in response to the recognition of aberrant DNA species or cyclic dinucleotides (CDNs) in the cell’s cytosol. CDNs are produced by cGAS upon binding to cytosolic DNA, such as viral DNA or self-DNA originating from the nucleus or mitochondria, which may occur following cell division, as a result of DNA damage [15], or from damaged tumor cells [14]. DNA-damaging agents can lead to the release of nuclear DNA into the cytosol, which activates STING-dependent cytokine production and promotes phagocyte infiltration. This process may be crucial for the elimination of damaged cells and the generation of antitumor T cell responses [14]. However, chronic activation of the STING pathway may contribute to inflammation-driven cancer progression. As a result, both STING agonists and antagonists demonstrate significant antitumor activity across various cancer types [16].

STING is broadly expressed in numerous cells and tissues of both immune and non-immune origin [17,18]. In this study, we investigate the role and mechanism of STING in ADPKD by focusing on whether and how STING regulates immune responses and macrophage recruitment as well as cystic epithelial cell apoptosis and renal fibrosis in *Pkd1* mutant mouse kidneys, and testing whether targeting STING with a specific inhibitor delays cyst growth in two *Pkd1* mutant mouse models.

## 2. Material and Methods

### 2.1. Cell Culture and Reagents

*Pkd1* WT and *Pkd1* null MEK cells were maintained as previously described in our recent publications [14,16,17]. *Pkd1* heterozygous PH2 cells and homozygous PN24 cells were provided by Stefan Somlo through the George M. O’Brien Kidney Center at Yale University (New Haven, CT, USA) and were cultured according to previously established protocols [14]. C-176 was obtained from Cayman (Catalog ID: 25859) and dissolved in DMSO (Sigma-Aldrich, St. Louis, MO, USA) to create a 20 mM stock solution. PN24 cells were treated with C-176 for 24 h, after which they were harvested and analyzed by Western blot. Antibodies used for Western blot analysis included the following: (a) anti-STAT3 (sc-482), anti-p65 (sc372 and sc-8008), and anti-fibronectin (sc-59826) purchased from Santa Cruz Biotechnology Inc (Dallas, TX, USA); (b) anti-STING (13647), anti-cGAS (31659), anti-TBK1 (3504), and anti-phosphorylated TBK1 (5483) purchased from Cell Signaling Technology (Danvers, MA, USA); (c) the antibodies for phosphorylated STAT3-Y705 (no. 9131), p65-S536 (no. 3031) also purchased from Cell Signaling Technology (Danvers, MA, USA); (d) anti-α-SMA (ab7817) was purchased from Abcam (Waltham, MA USA).

### 2.2. Western Blot Analysis

Cell pellets were collected and resuspended in a lysis buffer composed of 20 mM Tris-HCl (pH 7.4), 150 mM NaCl, 10% glycerol, 1% Triton X-100, 1 mM Na_3_VO_4_, 25 mM β-glycerol phosphate, 0.1 mM PMSF, Roche Complete Protease Inhibitor Set, and Sigma-Aldrich Phosphatase Inhibitor Set. The resuspended pellets were vortexed for 20 s, incubated on ice for 30 min, and then centrifuged at 20,000× *g* for 30 min. The supernatants were collected for Western blot analysis.

### 2.3. Histology and Immunohistochemistry (IHC)

Kidneys were fixed in 4% paraformaldehyde (pH 7.4). Paraffin-embedded sections (5 μm) were subjected to H&E and immunohistochemical (IHC) staining. For STING staining, a polyclonal rabbit anti-STING antibody (Cell Signaling Technology, Danvers, MA, USA; 1:200 dilution) and a biotinylated secondary antibody (Santa Cruz Biotechnology Inc. Dallas, TX, USA; 1:100 dilution) were utilized. Kidney sections were counterstained with hematoxylin. Images were captured and analyzed using a Nikon TI2-E microscope (Minato City, Japan).

### 2.4. Immunofluorescence Staining

For STING staining, a rabbit anti-STING antibody (Catalog No. 13647, Cell Signaling Technology, Danvers, MA, USA) and an Alexa Fluor 488 anti-rabbit IgG secondary antibody were employed. Images were analyzed using ImageJ software (1.54g) [19]. Briefly, the “Split Channels” option was used to separate the color channels into distinct windows, allowing for individual quantitation of each fluorescent channel. An image stained with a nuclear stain was first used to determine the total number of cells in the tissue slice. Subsequently, the STING staining image was used to measure the mean fluorescence intensity (MFI) in the same tissue slice. The STING intensity was calculated as the ratio of the MFI to the total number of cells. For Ki67 staining, a rabbit anti-Ki67 antibody (Catalog No. ab15580; Abcam) and an Alexa Fluor 488 anti-rabbit IgG secondary antibody were utilized. Macrophages were detected by immunofluorescence staining using the pan-macrophage marker F4/80 (1:100, MCA497, BIO-RAD, Hercules, CA, USA). Following antigen retrieval, tissue sections were incubated overnight with a rat anti-mouse F4/80 antibody (Catalog No. 14-4801-82, Invitrogen, P36931, Carlsbad, CA, USA).; 1:100 dilution), then with an Alexa Fluor 555 anti-rat IgG secondary antibody, and mounted in ProLong Gold Antifade reagent with DAPI (Invitrogen, P36931, Carlsbad, CA, USA). For α-SMA staining, a mouse anti-α-SMA antibody and an Alexa Fluor 555 anti-mouse IgG secondary antibody were used. Images were captured and analyzed using a Nikon TI2-E microscope (Minato City, Japan).

### 2.5. Terminal Deoxynucleotidyl Transferase dUTP Nick-End Labeling (TUNEL) Assay

*Pkd1* heterozygous PH2 cells and homozygous PN24 cells were transfected with p53 siRNA for 24 h, and then treated with or without C-176. Cells and kidney sections were analyzed using the TUNEL assay, following the manufacturer’s protocols (In Situ Death Detection Kit; Roche Diagnostics, Roche, Basel, Switzerland). ProLong Gold Antifade reagent with DAPI (Invitrogen) was employed for mounting. Images were captured using a Nikon Eclipse 80i microscope (Minato City, Japan).

### 2.6. Quantitative Reverse-Transcription PCR (qRT-PCR)

Total RNA was extracted using either the RNeasy Plus Mini Kit (Qiagen, Hilden, Germany) or Trizol reagents. One microgram of total RNA was used for reverse transcription reactions in a 20 μL volume to synthesize complementary DNA (cDNA) using the iScript cDNA Synthesis Kit (Bio-Rad, Hercules, CA, USA) or the MicroRNA First-Strand Synthesis Kit (Takara Bio, San Jose CA, USA). RNA or miRNA expression profiles were analyzed by real-time PCR employing iTaq SYBR Green Supermix with ROX (Bio-Rad, Hercules, CA, USA) on an iCycler iQ Real-Time PCR Detection System. The PCR protocol consisted of 40 cycles of 10 s at 95 °C and 20 s at 60 °C, followed by a melting curve analysis and a cooling step. Each sample was assayed in triplicate, and experiments were conducted in triplicate as well. Target gene expression levels were normalized to the expression level of actin. All primers used are listed in Supplementary Table S1.

### 2.7. RNA Interference

RNA oligonucleotides specifically targeting mouse p53 were procured from Santa Cruz Biotechnology Inc., Dallas, TX, USA These oligonucleotides were transfected using the DharmaFECT siRNA Transfection Reagent (GE Healthcare, T-2001, Little Chalfont, Buckinghamshire, UK). Cells were harvested and analyzed 24 h after transfection by Western blotting.

### 2.8. Isolation and Analysis of Cytosolic DNA and mtDNA

To isolate cytosolic DNA, cells were washed twice with PBS and lysed on ice for 10 min in a buffer consisting of PBS, 0.02% saponin, 10 mM Tris-Cl (pH 8.0), and 5 mM EDTA. The lysate was centrifuged at 800× *g* for 10 min at 4 °C to separate the cytosolic fraction from nuclei and organelles. The supernatant was transferred to a fresh tube and further centrifuged at 16,000× *g* for 10 min at 4 °C to remove residual debris. DNA was purified from the supernatant through two phenol:chloroform:isoamyl alcohol extractions and then precipitated with ethanol. The DNA was resuspended in 10 mM Tris-HCl (pH 8.0), and a quantitative PCR was performed to amplify the mt-Rnr-2 mitochondrial genes. Measurements were recorded in triplicate for three biological replicates. Expression levels were normalized to the β-actin expression from the corresponding non-cytosolic fraction, which was prepared by lysing cellular pellets in 10 mM Tris-HCl (pH 8.0), 10 mM EDTA, 0.2% SDS, and 200 μg/mL proteinase K, followed by a 60 min digestion at 50 °C and subsequent double extraction with phenol:chloroform:isoamyl alcohol and ethanol precipitation.

### 2.9. Mouse Strain and Treatment

One-month-old *Pkd1^RC/RC^* mice were administered C-176 (3 μg/g) or DMSO via daily intraperitoneal injections from one to three months of age. Kidneys were harvested from three-month-old mice for subsequent analysis. *Pkd1^fl/fl^: Pkhd1-Cre* mice were generated by crossbreeding *Pkd1^fl/+^: Pkhd1-Cre* females with *Pkd1^fl/+^: Pkhd1-Cre* males. Neonatal mice were intraperitoneally injected daily with C-176 (3 μg/g) or DMSO from postnatal day 8 (PN8) to PN24, and kidneys were collected and analyzed at PN25. Littermate controls were utilized in all experiments. All animal procedures were conducted under IACUC protocol A00003756-18 (19 July 2021), A00003756-R24 (26 July 2024), approved by the Mayo Clinic IACUC, and in compliance with National Institutes of Health, United States Department of Agriculture, and Association for Assessment and Accreditation of Laboratory Animal Care guidelines.

### 2.10. Measurement of Cyst Area

Cyst volume was assessed in whole kidneys following H&E staining using Image-Pro Plus software (version 5.0, Media Cybernetics). The cyst area was calculated as (cyst area/total area) × 100%. Three sections from each kidney were analyzed per mouse.

### 2.11. Quantitative BUN Determination

Serum samples were diluted 5-fold with distilled water before the assay. Subsequently, 5 μL of water (blank), 5 μL of standard (50 mg/dl), and 5 μL of sample were pipetted in triplicate into wells of a clear-bottom 96-well plate. Approximately 200 μL of working reagent was added to each well, mixed gently, and the plate was incubated for 20 min at room temperature. Optical density was measured at 520 nm.

### 2.12. Ultrasonic Testing in Mice

All imaging studies were conducted on live mice under isoflurane anesthesia. All imaging studies were performed on live animals under isoflurane anesthesia. Mice were positioned in dorsal recumbency, and hair removal cream was applied to the abdominal skin to ensure complete hair removal. A coupling gel was then applied to the skin surface to enhance transducer contact. Imaging was conducted using a commercially available ultrasound unit (Vevo 3100, FUJIFILM VisualSonics, Toronto, ON, Canada). An MX250s probe was selected for its relatively small footprint, which facilitated better skin contact on the mice. Imaging depth was standardized at 2 cm, and preset gain settings were used to ensure consistency for intermouse image comparison and quantification. Bilateral kidney length, width, and height were recorded to calculate total kidney volumes.

### 2.13. Statistics

Data are presented as mean ± SEM. Statistical analyses were performed using GraphPad Prism version 10. *p*-values were determined using an unpaired Student’s *t*-test and a one-way ANOVA, with a *p*-value of less than 0.05 considered statistically significant.

### 2.14. Study Approval

All animal procedures were approved by the Laboratory Animal Resources of Mayo Clinic and conducted in accordance with Institutional Animal Care and Use Committee regulations.

## 3. Result

### 3.1. STING Expression Was Upregulated in Pkd1 Mutant Mouse Kidneys

**Figure 1:**
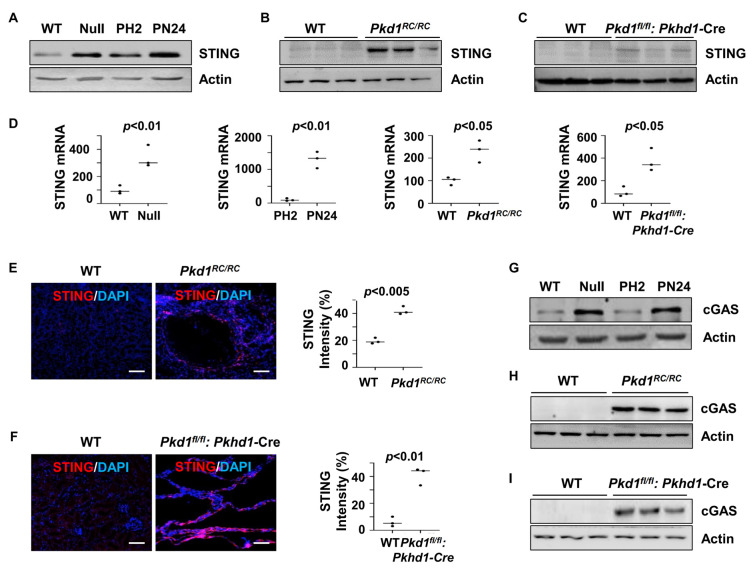
Pkd1 mutant renal epithelial cells and tissues demonstrated increased expression of STING. (**A**) Western blot analysis of the protein expression of STING from whole-cell lysates of *Pkd1* wild type (WT) and *Pkd1*null MEK cells as well as in postnatal *Pkd1* heterozygous PH2 cells and *Pkd1* homozygous PN24 cells. (**B**,**C**) Western blot analysis of the protein expression of STING in three-month-old kidneys from *Pkd1^RC/RC^* mice compared to WT mice (**B**) and in PN25 kidneys from *Pkd1^fl/fl^*: *Pkhd1*-Cre mice compared to *Pkd1^fl/+^*: *Pkhd1*-Cre (WT) mice (**C**). (**D**) qRT-PCR analysis of the expression of STING mRNA in *Pkd1* wild type and Null MEK cells (*p* < 0.01), PH2 cells and PN24 (PN24) cells (*p* < 0.01) as well as in kidneys from three-month-old *Pkd1^RC/RC^* mice (*p* < 0.05) and PN25 *Pkd1^fl/fl^*: *Pkhd1*-Cre mice (*p* < 0.05) compared to that in kidneys from age-matched wild type mice. Statistical analysis was performed using an unpaired 2-tailed Student’s *t* test. (**E**,**F**) STING expression was increased in cyst-lining epithelial cells in *Pkd1* mutant kidneys as examined by immunofluorescence staining with anti-STING antibody in three-month-old kidneys from WT and *Pkd1^RC/RC^* mice (*p* < 0.005) as well as in PN25 kidneys from WT and *Pkd1^fl/fl^*: *Pkhd1*-Cre (Flox) mice (*p* < 0.01). Scale bars: 100 μm. Statistical analysis was performed using an unpaired 2-tailed Student’s *t* test. (**G**–**I**) Western blot analysis of the protein expression of cGAS from whole-cell lysates of *Pkd1* null MEK cells compared to WT MEK cells and from PN24 cells compared to PH2 cells as well as in three-month-old kidneys from *Pkd1^RC/RC^* mice compared to that in kidneys from WT mice and in PN25 kidneys from *Pkd1^fl/fl^*: *Pkhd1*-Cre (Flox) mice compared to that in kidneys from age-matched WT mice. Original Western blot Images can be found in Supplementary File S1.

To assess the role of STING in PKD, we examined its expression in *Pkd1* mutant renal epithelial cells and tissues. Our findings revealed that STING expression was elevated in *Pkd1* null mouse embryonic kidney (MEK) cells and postnatal *Pkd1* homozygous PN24 cells compared to *Pkd1* wild-type (WT) MEK cells and postnatal *Pkd1* heterozygous PH2 cells. Additionally, increased STING expression was observed in the kidneys of *Pkd1^RC/RC^* mice and *Pkd1^fl/fl^: Pkhd1-Cre* mice compared to age-matched controls, as determined by western blot (Figure 1A–C) and quantitative reverse transcription PCR (qRT-PCR) (Figure 1D). The upregulation of STING in cyst-lining epithelial cells in the kidneys of *Pkd1^RC/RC^* and *Pkd1^fl/fl^: Pkhd1-Cre* mice was further confirmed by immunofluorescence staining (Figure 1E,F). Moreover, the expression of cGAS, a key component of the cGAS-STING pathway, was elevated in *Pkd1* mutant renal epithelial cells and tissues, as shown by the western blot analysis (Figure 1G–I). These findings suggest the activation of the STING pathway in *Pkd1* mutant kidneys.

### 3.2. Treatment with an Inhibitor of STING, C-176, Delays Cyst Growth in Pkd1 Mutant Mouse Kidneys

**Figure 2:**
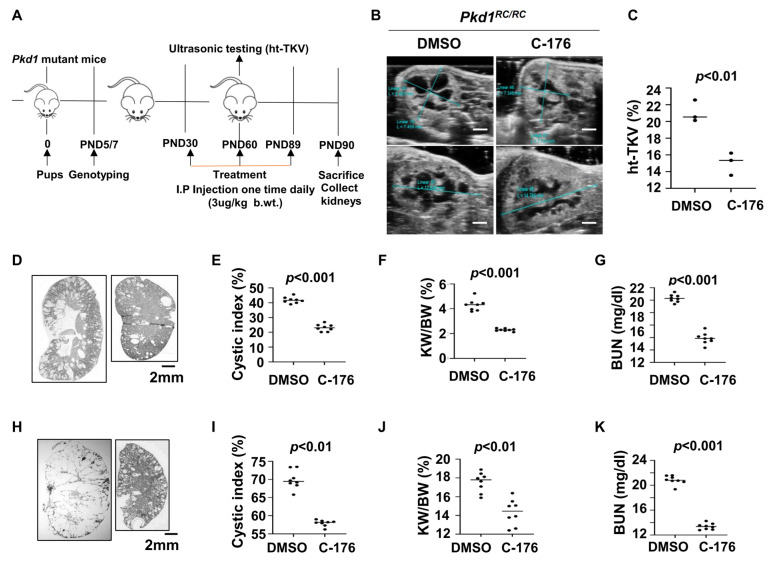
Treatment with an inhibitor of STING, C-176, delays cyst growth in *Pkd1* mutant kidneys. (**A**) Protocol of the treatment with STING inhibitor C-176 to the *Pkd1^RC/RC^* mice. (**B**) Ultrasound examination of PN60 kidneys from *Pkd1^RC/RC^* mice treated with vehicle (DMSO) (n = 3) or C-176 (n = 3). Scale bar: 2 mm. (**C**) Kidney height to total kidney volume (ht-TKV) ratios were decreased in PN60 kidneys from *Pkd1^RC/RC^* mice treated with C-176 (n = 3) compared with age-matched mice treated with DMSO (n = 3). *p <* 0.01. (**D**) Histologic examination of PN90 kidneys from *Pkd1^RC/RC^* mice treated with vehicle (DMSO) (n = 8) and C-176 (n = 8). Scale bar: 2 mm. (**E**) Quantification of the percentage of cystic areas over total kidney-section areas of PN90 kidneys from *Pkd1*^RC/RC^ mice treated as in (**D**). Shown is mean ± SEM of all sections quantified for each condition. *p <* 0.001. (**F**,**G**) Kidney weight to body weight (KW/BW) ratios (**F**) and blood urea nitrogen (BUN) levels (**G**) were decreased in PN90 kidneys from *Pkd1^RC/RC^* mice treated with C-176 compared with age-matched mice treated with DMSO (control). *p* < 0.01. (**H**) Histologic examination of PN25 kidneys from *Pkd1^fl/fl^: Pkhd1*-Cre mice treated with vehicle (DMSO) (n = 8) and C-176 (n = 8). Scale bar: 2 mm. (**I**) Quantification of the percentage of cystic areas over total kidney section areas of PN25 kidneys from *Pkd1^fl/fl^*: *Pkhd1*-Cre mice treated as in (**H**). Shown is mean ± SEM of all sections quantified for each condition. *p < 0.01*. (**J**,**K**) KW/BW ratios (**J**), and BUN levels (**K**) were decreased in PN25 kidneys from *Pkd1^fl/fl^*: *Pkhd1*-Cre treated with C-176 compared with age-matched mice treated with DMSO (control). *p* < 0.01. Statistical analysis was performed using an unpaired 2-tailed Student’s *t* test.

For the upregulation of STING in cystic renal epithelial cells and tissues, we investigated whether targeting STING with its inhibitor, C-176 [20], delayed cyst growth in vivo. We first treated *Pkd1^RC/RC^* mice with C-176 (3 μg/kg) (n = 8) or vehicle (n = 8) by daily intraperitoneal (IP) injections from postnatal day 30 (PN30) to PN89 and collected kidneys at PN91 (Figure 2A). The dosage, route, and timing of C-176 administration to *Pkd1* mutant mice were based on a previous publication [21]. Height-adjusted total kidney volume (ht-TKV) has been used to predict the progression of cyst growth in ADPKD [22,23]. We analyzed the ht-TKV in *Pkd1^RC/RC^* mice at PN60 with an ultrasound and found that the ht-TKV values were decreased in C-176 treated *Pkd1^RC/RC^* mice compared with age-matched vehicle-treated control mice (Figure 2B,C). We further found that treatment with C-176 (n = 8) delayed cyst growth as seen by a decrease of cystic index (Figure 2D,E), kidney weight/body weight (KW/BW) ratios (Figure 2F), and blood urea nitrogen (BUN) levels (Figure 2G) in PN91 *Pkd1^RC/RC^* mice compared to age-matched *Pkd1^RC/RC^* mice treated with vehicle.

To extend the translational significance of the in vivo findings, we also treated early stage *Pkd1^fl/fl^: Pkhd1-Cre* mice with C-176 (3 μg/kg) (n = 8) or vehicle (n = 8) by daily intraperitoneal injection from postnatal day 8 (PN8) to PN24 and sacrificed the mice at PN25. Treatment with C-176 also decreased cyst growth as seen by a decrease of the cystic index, KW/BW, and BUN levels in *Pkd1^fl/fl^: Pkhd1-Cre* mice compared to that in age-matched vehicle-treated control mice (Figure 2H–K). These results suggest that targeting STING with a pharmacological inhibitor may delay cyst growth in ADPKD patients.

### 3.3. Treatment with the STING Inhibitor C-176 Reduces Cytokine Expression and Decreases Macrophage Recruitment in Pkd1 Mutant Kidneys

**Figure 3:**
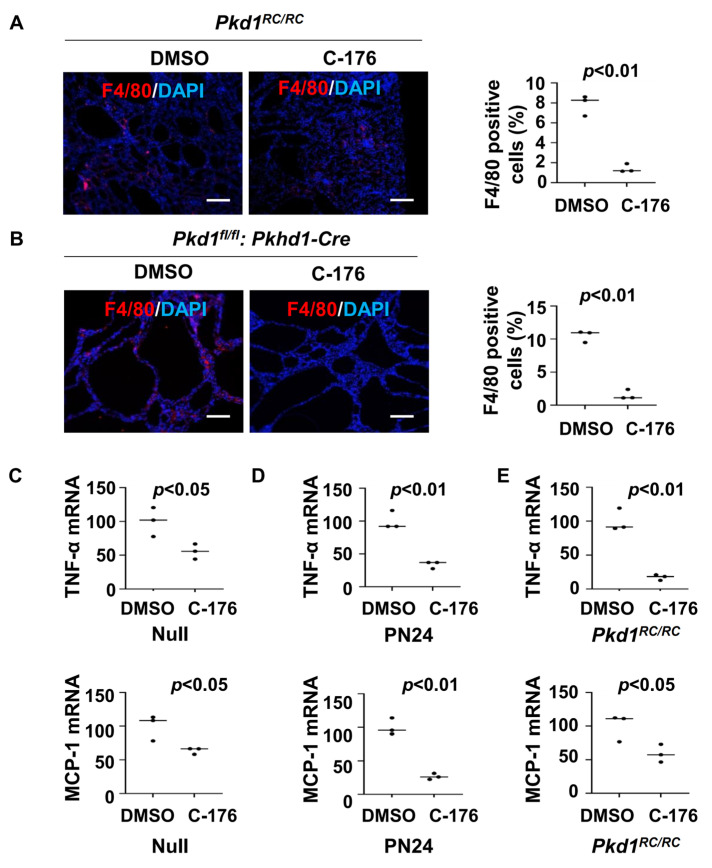
Treatment with C-176 decreases the recruitment of macrophages in *Pkd1* mutant kidneys. (**A**) F4/80 staining of kidneys from *Pkd1^RC/RC^* mice treated with C-176 (n = 3) or vehicle (n = 3). *p* < 0.01. Scale bars: 100 μm. (**B**) F4/80 staining of kidneys from *Pkd1^fl/fl^: Pkhd1-Cre* mice treated with C-176 (n = 3) or vehicle (n = 3). *p* < 0.05. Scale bars: 100 μm. (**C**) qRT-PCR analysis of the expression of TNF-α and MCP-1 in *Pkd1* null MEK cells treated with C-176 and vehicle. Data were analyzed from three experiments. *p* < 0.05. (**D**) qRT-PCR analysis of the expression of TNF-α and MCP-1 in postnatal *Pkd1* homozygous PN24 cells treated with C-176 and vehicle. Data were analyzed from three experiments. *p < 0.01*. (**E**) qRT-PCR analysis of the expression of TNF-α and MCP-1in kidneys of *Pkd1*^RC/RC^ mice treated with C-176 and vehicle. *p* < 0.05. All statistical data are represented as mean ± SEM, and *p*-values are calculated by two-tailed unpaired *t* tests.

Previous studies have indicated a close relationship between STING and macrophages [24,25]. The recruitment of M2-like macrophages to interstitial and pericystic regions is increased in orthologous ADPKD mouse models, leading to promoted cyst growth [6]. We found that treatment with STING inhibitor C-176 decreased the recruitment of macrophages in *Pkd1* mutant kidneys compared to that in age-matched control kidneys treated with vehicle as analyzed with F4/80 immunofluorescence staining (Figure 3A,B). This result suggests that STING signaling contributes to the recruitment of macrophages in ADPKD kidneys.

Cytokines, such as TNF-α, IL-6, and MCP-1, play crucial roles in renal inflammation within cystic kidneys [26], which can regulate the recruitment of macrophages in *Pkd1* mutant kidneys [6]. We found that treatment with STING inhibitor C-176 decreased the expression of TNF-α and MCP-1 mRNA in *Pkd1* null MEK cells and PN24 cells compared to that in vehicle-treated control cells (Figure 3C,D) as well as in *Pkd1^RC/RC^* kidneys compared to that in vehicle-treated age-matched control kidneys as analyzed with the qRT-PCR analysis (Figure 3E). These results suggest that the activation of the STING pathway may stimulate the recruitment of macrophages through the regulation of the expression of cytokines, thereby promoting cyst progression.

### 3.4. Treatment with STING Inhibitor C-176 Alleviates the Inflammatory Response Possibly through NF-κB Pathway

**Figure 4:**
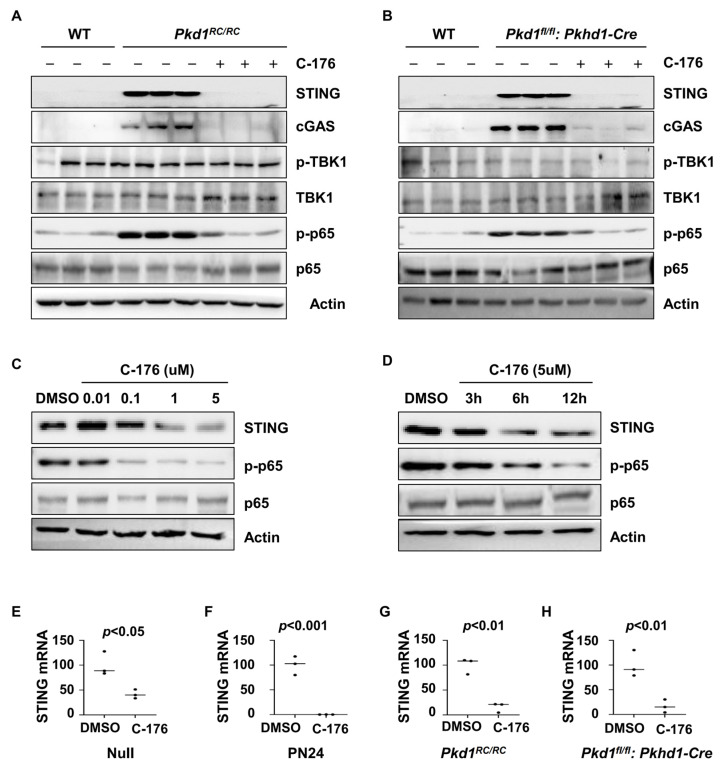
Treatment with C-176 alleviates the inflammatory response through the STING/NF-κB pathway. (**A**,**B**) Western blot analysis of the expression of STING, p-TBK1, TBK1, p-p65, and p65 in kidneys from PN91 *Pkd1^RC/RC^* mice (**A**) and PN25 *Pkd1^fl/fl^: Pkhd1-Cre* mice (**B**) treated with C-176 and vehicle as well as in kidneys from age-matched WT mice. (**C**,**D**) Western blot analysis of the expression of STING, p-p65, and p65 in PN24 cells treated with different concentrations (**C**) of C-176 at different time points (**D**). (**E**,**F**) qRT-PCR analysis of the expression of STING mRNA in *Pkd1* null MEK cells (**E**) (*p* < 0.05) and *Pkd1* homozygous PN24 (PN24) cells (**F**) (*p* < 0.001) treated with C-176 and DMSO, as well as in three-month-old kidneys from *Pkd1*^RC/RC^ mice (*p* < 0.01) (**G**) and PN25 kidneys from *Pkd1^fl/fl^*: *Pkhd1*-Cre (*p* < 0.01) treated with C-176 and DMSO (**H**). Statistical analysis was performed using an unpaired 2-tailed Student’s *t* test. Original Western blot Images can be found in Supplementary File S1.

It has been reported that the activation of STING may lead to inflammatory responses through two main pathways, including STING mediated TBK1/IRF3 and IKK/NF-κB pathways [27,28]. We found that treatment with C-176 decreased the phosphorylation of the p65 subunit of NF-κB but not the phosphorylation of TBK1 in the kidneys of *Pkd1 ^RC/RC^* and *Pkd1^fl/fl^: Pkhd1-Cre* mice as examined with western blot analysis (Figure 4A,B). Treatment with C-176 decreased the phosphorylation of p65 in *Pkd1* mutant PN24 cells in a dose (Figure 4C) and time-dependent manner (Figure 4D). These results suggest that the activation of STING regulates the inflammatory response in ADPKD kidneys through NF-κB signaling but not TBK1 signaling. In addition, treatment with C-176 also decreased STING protein and mRNA in *Pkd1* mutant cells and kidneys compared to vehicle-treated controls as analyzed with a qRT-PCR (Figure 4E–H).

### 3.5. Treatment with STING Inhibitor C-176 Alleviates Renal Fibrosis in Pkd1 Mutant Mouse Kidneys

**Figure 5:**
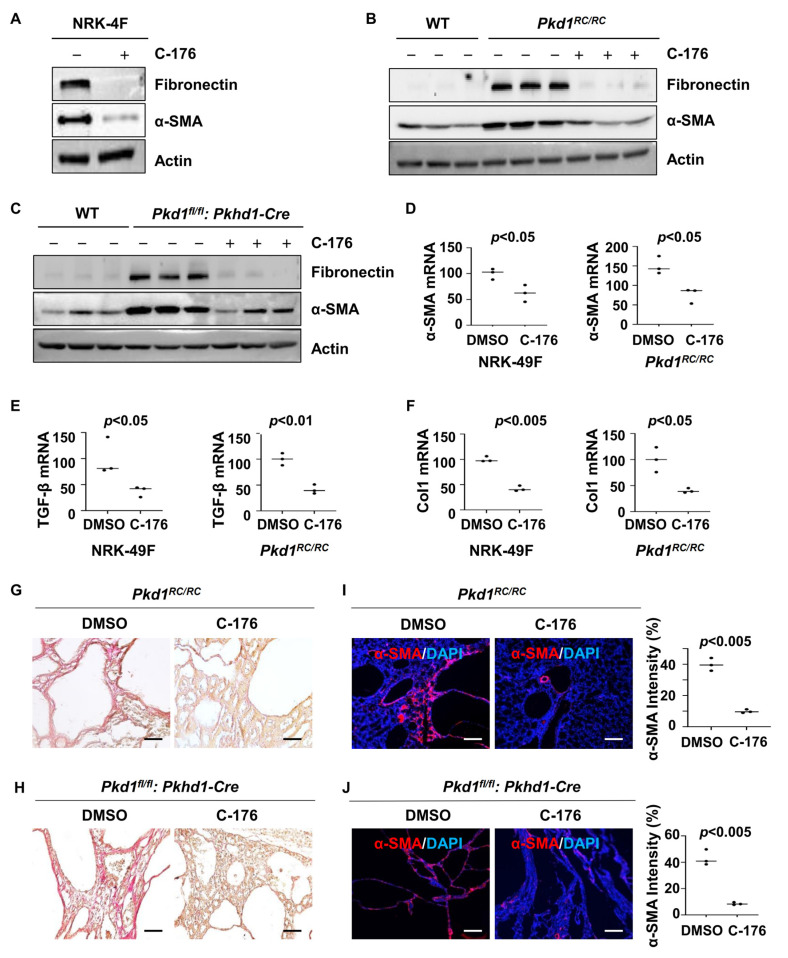
Treatment with C-176 decreases fibrosis in *Pkd1* mutant kidneys and inhibits the activation of NRK-49F cells. (**A**) Western blot analysis of the expression of α-SMA and fibronectin in whole-cell lysates of rat kidney fibroblasts (NRK-49F) treated with C-176 and vehicle. (**B**) Western blot analysis of the expression of α-SMA and fibronectin in kidneys from *Pkd1^RC/RC^* mice treated with D-176 or vehicle. (**C**) Western blot analysis of the expression of α-SMA and fibronectin in kidneys from *Pkd1^fl/fl^: Pkhd1-Cre* mice treated with D-176 or vehicle. (**D**–**F**) qRT-PCR analysis of α-SMA (**D**), TGF-β (**E**), and collagen 1 (**F**) mRNA in NRK-49F cells treated with C-176 and vehicle as well as in kidneys from *Pkd1*^RC/RC^ mice treated with C-176 and vehicle. *p < 0.05*. All data of NRK-49F cells were analyzed from three experiments. (**G**,**H**) Picrosirius red staining revealed a decrease of renal fibrosis in kidneys of *Pkd1*^RC/RC^ mice (**G**) and *Pkd1^fl/fl^: Pkhd1-Cre* mice (**H**) treated with C-176 compared to that in kidneys of age-matched control mice treated with vehicle. Scale bars: 50 μm. (**I**,**J**) Immunostaining of α-SMA in kidneys from *Pkd1^RC/RC^* mice (**I**) and *Pkd1^fl/fl^: Pkhd1-Cre* mice (**J**) treated with C-176 compared to that in kidneys from age-matched vehicle-treated mice. Scale bars: 100 μm. All statistical data are represented as mean ± SEM, and *p*-values are calculated by unpaired Student’s *t*-test. Original Western blot Images can be found in Supplementary File S1.

Renal fibrosis represents a final common pathway leading to end-stage renal failure in ADPKD. We found that treatment with C-176 decreased the expression of fibrotic markers, including fibronectin and α-SMA, in rat kidney fibroblasts (NRK-49F) compared to that in cells treated with vehicle as well as in kidneys from *Pkd1^RC/RC^* mice or *Pkd1^fl/fl^: Pkhd1-Cre* mice compared to that in kidneys from vehicle-treated age-matched control mice as analyzed with western blot analysis (Figure 5A–C). Treatment with C-176 also decreased the mRNAs of α-SMA, TGF-β, and Col1 in NRK-49F cells and kidneys from *Pkd1^RC/RC^* mice as analyzed with qRT-PCR analysis (Figure 5D–F). The administration of C-176 decreased renal fibrosis as seen by a reduction of extracellular matrix deposition and a decrease of the expression of α-SMA in both *Pkd1^RC/RC^* and *Pkd1^fl/fl^: Pkhd1-Cre* kidneys compared to that in control kidneys treated with vehicle as examined by picrosirius red (Figure 5G,H) and α-SMA immunofluorescence staining analysis (Figure 5I,J).

### 3.6. Inhibition of STING Induces p53-Dependent Cystic Renal Epithelial Cell Apoptosis

**Figure 6:**
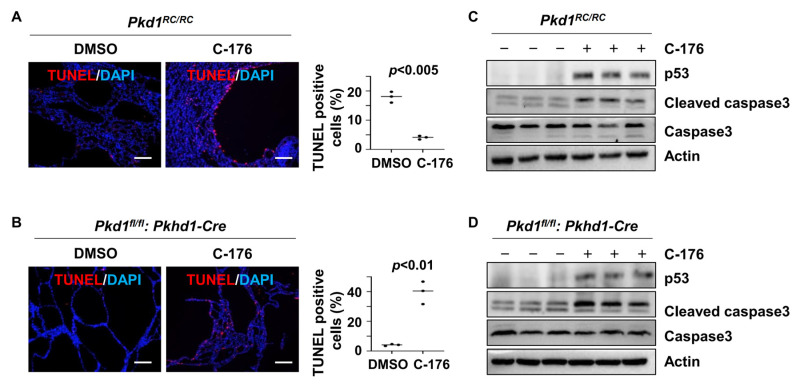
Inhibition of STING induces cystic renal epithelial cell apoptosis in *Pkd1* mutant mouse kidneys. (**A**,**B**) Treatment with C-176 induced cyst-lining epithelial cell apoptosis in *Pkd1^RC/RC^* (**A**) and *Pkd1^fl/fl^: Pkhd1-Cre* (**B**) kidneys, as detected by TUNEL assay. *p* < 0.01. Scale bars: 100 μm. Statistical analysis was performed using an unpaired 2-tailed Student’s *t* test. (**C**,**D**) Western blot analysis of the expression of p53, cleaved caspase-3, and caspase-3in *Pkd1^RC/RC^* (**C**) and *Pkd1^fl/fl^: Pkhd1-Cre* kidneys (**D**) treated with C-176 and vehicle. The levels of p53 and cleaved caspase 3 were increased in kidneys from *Pkd1^RC/RC^* mice (**C**) and *Pkd1^fl/fl^: Pkhd1-Cre* mice (**D**) treated with C-176 compared to those in kidneys from age-matched control mice treated with vehicle. Original Western blot Images can be found in Supplementary File S1.

We also found that treatment with C-176 induced cyst-lining epithelial cell death in kidneys from *Pkd1^RC/RC^* and *Pkd1^fl/fl^: Pkhd1-Cre* mice compared to that in kidneys from age-matched control mice treated with vehicle as analyzed by the terminal-deoxynucleotidyl transferase dUTP nick end labeling (TUNEL) assay (Figure 6A,B). We further found that treatment with STING inhibitor C-176 increased the expression of p53 and the cleavage of caspase 3 in kidneys from *Pkd1^RC/RC^* and *Pkd1^fl/fl^: Pkhd1-Cre* mice compared to those in kidneys from age-matched control mice treated with vehicle (Figure 6C,D).

**Figure 7:**
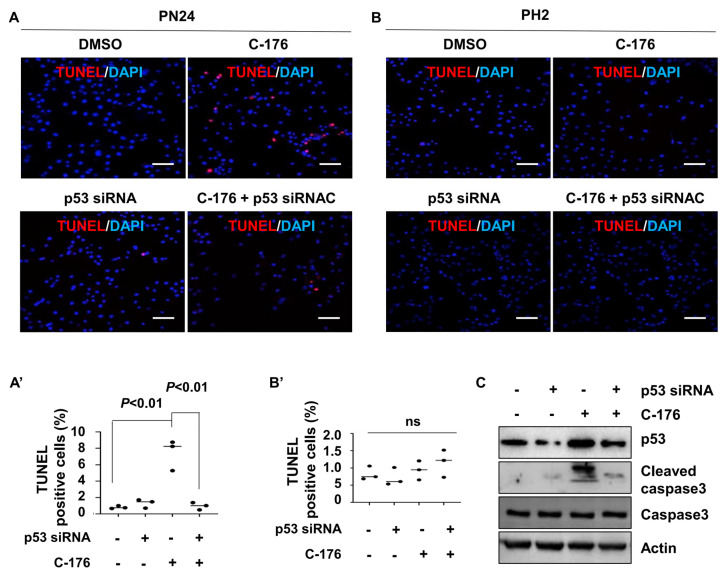
Inhibition of STING induces p53−dependent cystic renal epithelial cell apoptosis in *Pkd1* mutant cells. (**A**,**A’**) Inhibition of STING with C-176 induced apoptosis in postnatal *Pkd1* homozygous PN24 cells, while knockdown of p53 with siRNA prevented C-176-induced apoptosis in these cells, as detected by TUNEL assay. Cells were transfected with p53 siRNA for 24 h and then treated with C-176 for 24 h. *p* < 0.01. Statistical analysis was performed using an unpaired 2-tailed Student’s *t* test. Scale bar: 100 μm. (**B**,**B’**) Inhibition of STING with C-176 did not induce apoptosis in postnatal *Pkd1* heterozygous PH2 cells as detected by TUNEL assay. Cells were transfected with p53 siRNA for 24 h and then treated with 5 μM C-176 for 24 h. *p* = ns. Statistical analysis was performed using an unpaired 2-tailed Student’s *t* test. (**C**) Western blot analysis reveals increased levels of p53 and cleaved caspase 3 in PN24 cells treated with C-176 compared to vehicle-treated cells. Knockdown of p53 with siRNA prevents the increase of p53 and cleaved caspase 3 in these cells, as detected by western blot. Original Western blot Images can be found in Supplementary File S1.

To investigate how the inhibition of STING induced cystic renal epithelial cell death in kidneys from *Pkd1^RC/RC^* mice or *Pkd1^fl/fl^: Pkhd1-Cre* mice and the involvement of p53 in this process, we knocked down p53 with siRNA in a *Pkd1* mutant PN24 cell and then treated with C-176. Our study revealed that inhibiting STING with C-176 induced apoptosis in *Pkd1* mutant PN24 cells (Figure 7A). However, the knockdown of p53 using siRNA significantly reduced apoptosis in *Pkd1* mutant renal epithelial cells treated with C-176 (Figure 7A). Notably, no apoptotic cells were observed in PH2 cells under any of these treatments, as determined by the TUNEL assay (Figure 7B). The efficiency of p53 siRNA knockdown and the levels of cleaved caspase-3 protein were confirmed by western blot analysis (Figure 7C). These results suggest that the activation of STING promotes cystic cell death through p53 signaling.

### 3.7. Mitochondria Impairment and Cytosolic DNA Trigger the Activation of STING in Pkd1 Mutant Renal Epithelial Cells and Tissues

**Figure 8:**
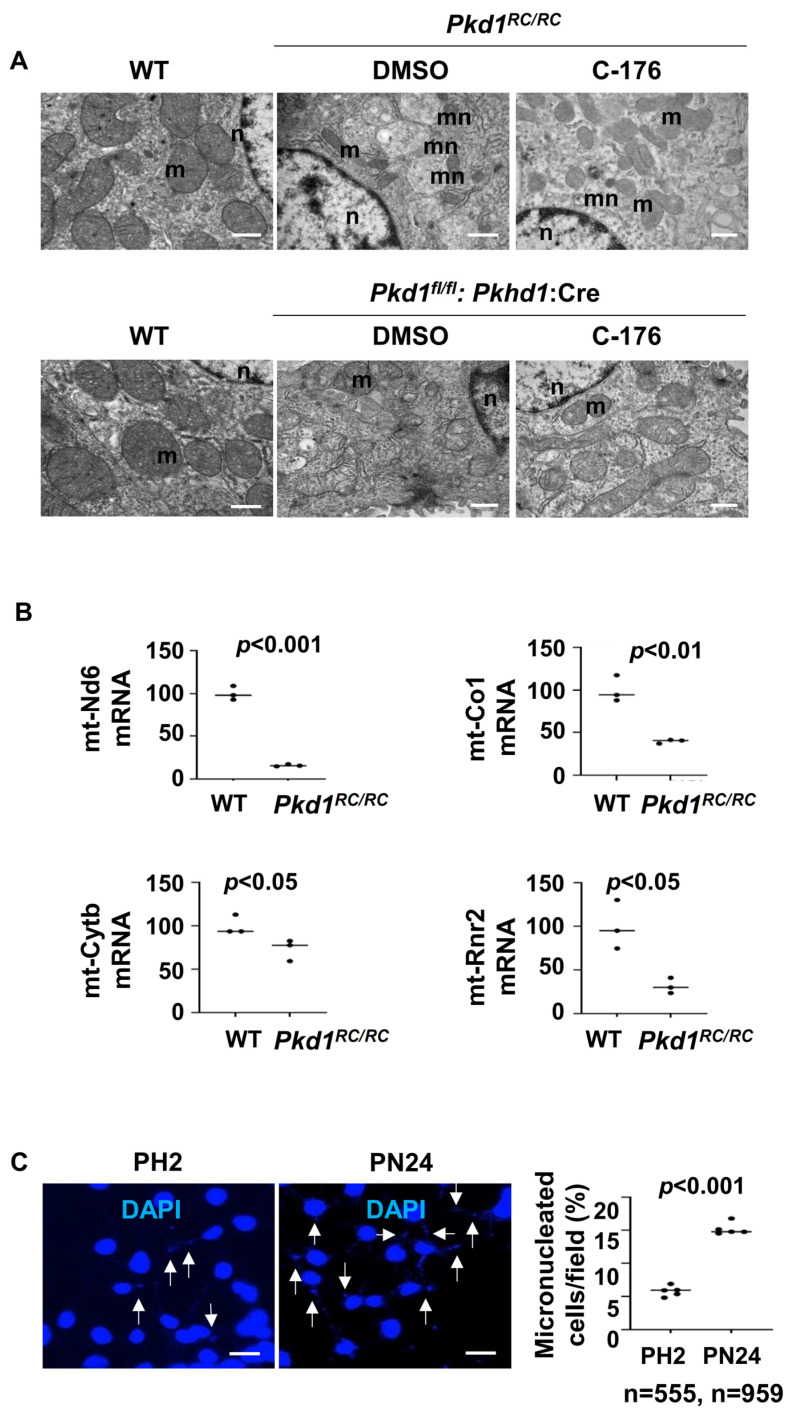
Mitochondria impairment and micronuclei are increased in *Pkd1* mutant mouse kidneys. (**A**) Transmission electron micrographs (TEM) analysis demonstrated that the morphology of mitochondria (m) was changed in renal epithelia cells in *Pkd1^RC/RC^* and *Pkd1^fl/fl^: Pkhd1-Cre* kidneys compared to that in age-matched wild type kidneys, Additionally, micronuclei (mn) were significantly increased in renal epithelia cells in *Pkd1^RC/RC^* compared to those in age-matched wild type kidneys, while treatment with C-176 led to a reversal of these changes in *Pkd1^RC/RC^* and *Pkd1^fl/fl^: Pkhd1-Cre* kidneys. Scale bars: 200 μm. m: mitochondrial. n: nuclear. mn: micronuclei. (**B**) Relative mRNA levels of mitochondrial oxidative phosphorylation (OXPHOS)-associated genes (mt-Nd6, mt-Co1, mt-Cytb, mt-Rnr2) in kidneys from *Pkd1^RC/RC^* mice were increased compared to WT mice (*p* < 0.05). (**C**) The DAPI staining experiment show the presence of micronuclei is significantly increased in postnatal *Pkd1* homozygous PN24 cells compared to PH2 cells. The arrow points to the micronucleus in the cell. Each data point indicates a different field from independent experiments. “n” denotes the total number of cells counted (*p* < 0.001). Scale bar: 100 μm. All statistical data are represented as mean ± SEM, and *p*-values are calculated by unpaired Student’s *t*-test.

The activation of STING is known to be dependent on the presence of cytosolic DNA, either of genomic or mitochondrial origin [28]. Various studies have reported significant alterations in in the morphology and function of mitochondria in *Pkd1*-deficient kidneys [29,30], which may be caused by an injury in the mitochondria, leading to the release of mtDNA to cytosol [31]. We observed that the mitochondria exhibited intermingled and abnormal structures, such as swelling and damage to cristae, in *Pkd1* mutant kidneys compared to that in kidneys from wild type mice under transmission electron microscopy (Figure 8A). Treatment with C-176 could normalize mitochondria morphology characterized by the decrease of mitochondrial swollen and damaged cristae (Figure 8A). We also observed higher numbers of micronuclei (mn) in kidneys from *Pkd1* mutant *Pkd1^RC/RC^* and *Pkd1^fl/fl^: Pkhd1-Cre* mice than those in wild type kidneys under transmission electron microscopy (Figure 8A), and treatment with STING inhibitor C-176 decreased the number of micronuclei in kidneys from *Pkd1* mutant mice (Figure 8A). The transcription of mitochondrial genes, including *mt-Nd6, mt-Co1, mt-Cytb*, *and mt-Rnr2,* was decreased in kidneys from *Pkd1* mutant mice compare to that in kidneys from wild type mice (Figure 8B). In addition, we found a higher number of micronuclei in *Pkd1* mutant PN24 cells compared to PH2 cells as examined with DAPI staining (Figure 8C).

## 4. Discussion

**Figure 9:**
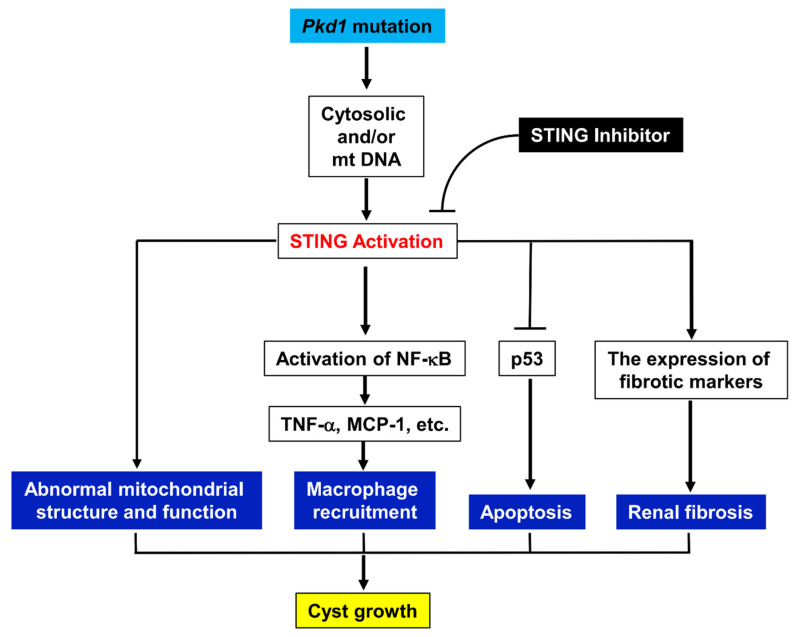
Working model of the STING regulates cyst growth in ADPKD. *Pkd1* mutation results in an upregulation and activation of STING in *Pkd1* mutant mouse kidneys. STING promotes cyst growth through the following: (1) the activation of NF-κB signaling in *Pkd1* mutant cells; (2) the increase of the recruitment of macrophages in interstitial and peri-cystic regions in *Pkd1* mutant mouse kidneys via NF-κB that mediated the upregulation of TNF-α and MCP-1; (3) the inhibition of *Pkd1* mutant renal epithelial cell death via p53 signaling; (4) the impairment of mitochondrial structure and function; and (5) the increase of renal fibrosis. Targeting STING with its specific inhibitor C-176 delays cyst growth by the normalization of these processes in *Pkd1* mutant kidneys. The upregulation and activation of STING is stimulated by the cytosolic DNAs of nuclear and mitochondrial origin.

STING, a key signaling component of the intracellular DNA sensing pathway, along with its downstream adaptors, plays a crucial role in the immune defense against endogenous damage-associated DNA and microbial DNA. It is also implicated in various immune-related diseases and cancers [32]. However, the role and mechanisms of STING signaling in ADPKD remain poorly understood. In this study, we demonstrate that STING is upregulated in *Pkd1* mutant mouse kidneys. Additionally, targeting STING with the specific inhibitor C-176 delays cyst growth in both an early-stage, aggressive *Pkd1* conditional knockout mouse model and a milder, long-lasting *Pkd1* mutant mouse model. As summarized in Figure 9, STING regulates cyst growth through the following: (1) the activation of NF-κB but not TBK1 signaling in *Pkd1* mutant cells; (2) the increase of the expression of cytokines, including TNF-α and MCP-1, mediated by NF-κB; (3) the recruitment of macrophages in interstitial and peri-cystic regions in *Pkd1* mutant mouse kidneys; (4) the inhibition of *Pkd1* mutant renal epithelial cell death via p53 signaling. In addition, targeting STING with its specific inhibitor C-176 decreases renal fibrosis in *Pkd1* mutant mouse kidneys via a decrease of the expression of fibrotic markers, including TGF-β, α-SMA, collegen1, and fibronectin, resulting in a decrease of extracellular matrix deposition in *Pkd1* mutant kidneys. Targeting STING also normalizes mitochondrial structure and function in *Pkd1* mutant kidneys. The upregulation and activation of STING may be stimulated by DNAs of nuclear and mitochondrial origin. These results indicate that STING plays a pivotal role in promoting the progression of ADPKD, suggesting it may be a promising novel therapeutic target for the treatment of this disease.

The activation of STING is triggered by cytosolic DNA, resulting in the translocation of STING from the endoplasmic reticulum to the Golgi apparatus, where it stimulates an immune response [33]. In general, DNA is predominantly confined to the nucleus and mitochondria and is rapidly degraded by nucleases within the cytosol and endo-lysosomal compartments. However, the undegraded cytosolic DNA produced by bacteria or by the mammalian enzyme cGAS can induce the activation of STING by the binding to its LBD domain [13], which causes a conformational change and allows STING to form a high-order oligomer and translocates from the ERto the Golgi [33]. In addition, STING can be activated by the following: (1) extracellular self-DNA, in which the entry of extracellular DNA into cytosol is often associated with cell death or perturbed phagocytic digestion; (2) mitochondrial DNA, which occurs during intrinsic apoptosis, resulting in a efflux of mtDNA into the cytosol; (3) nuclear chromatin; (4) cytosolic chromatin and micronuclei [15,28,34]. Micronuclei consist of chromatin fragments enclosed by a nuclear envelope-like structure. They are formed due to DNA double-strand breaks, mitotic errors, or issues with DNA replication, which can result in chromatin fragments or entire non-segregated chromosomes. These chromosomes may recruit their own nuclear envelopes during mitosis [35]. Elevated levels of cytosolic DNA, which can arise from mitotic stress in cancers, radiation therapy, cellular senescence, autoimmune disorders, or genotoxic stress, can lead to persistent activation of the cGAS–STING pathway. This activation results in chronic inflammation and the associated pathology. Additionally, mitochondrial dysfunction has been observed in ADPKD mouse kidneys [3]. However, whether ADPKD pathogenesis involves the release of mtDNA and activation of STING remains to be determined. In this study, we identified two primary sources of DNAs, including mitochondrial DNA and micronuclei, that might trigger STING activation in *Pkd1* mutant cells, supporting a mechanism of how the release of mtDNA promotes ADPKD progression. First, *PKD1* mutant cells exhibited significant metabolic alterations compared to normal renal epithelial cells, including heightened superoxide and oxidative stress levels [36], resulting in morphological and functional modifications of the mitochondria and leading to the release of mitochondrial DNA into the cytoplasm to activate the STING pathway in *Pkd1* mutant renal epithelial cells. Second, PKD1 mutations has been reported to increase DNA damage and genomic instability [37,38], leading to the formation of micronuclei. The DNA within these micronuclei is enclosed by a nuclear membrane [39]. Genomic instability in *PKD1* mutation cells results in the damage of the micronuclear envelope and then in the exposure of the micronuclear DNA directly to the cytoplasm to activate the STING pathway in *Pkd1* mutant renal epithelial cells.

STING activation and subsequent signal transduction is best understood for its capacity to trigger the activation of TBK1/IRF3 and NF-κB [40]. It has been reported that STING triggers the activation of NF-κB to a lesser extent than other pattern recognition receptor (PRR) cascades. Unlike other pattern recognition receptor (PRR) systems that assemble TRAF (tumor necrosis factor receptor-associated factor)-dependent signalosomes to recruit the TAK (transforming growth factor-β-activated kinase) and IKK (inhibitor of NF-κB kinase) complexes for NF-κB activation, mammalian STING utilizes an unconventional signaling pathway to activate this transcription factor [40]. However, the mechanisms of STING-dependent NF-κB activation remain unresolved. Some studies suggest that TBK1 functions upstream of NF-κB activation, while other studies propose a TBK1-independent mechanism [41]. In addition, it has been proposed that STING-dependent NF-κB activation may be governed by signals from the ligand-binding domains (LBD) of oligomerized STING [28]. Our results support TBK1-independent mechanisms of STING-mediated NF-κB activation in *Pkd1* mutant renal epithelia cells and kidneys. In addition, we found that treatment with STING inhibitor C-176 not only decreased the phosphorylation and activation of NF-κB but also the expression of STING mRNA and protein in *Pkd1* mutant renal epithelia cells and kidneys. C-176 is a selective and blood-brain barrier permeable STING inhibitor, which covalently targets transmembrane cysteine residue 91, thereby blocking activation-induced palmitoylation of STING [20]. How treatment with C-176 decreases the expression of STING is unknown. The results show that targeting STING normalizes mitochondrial structure and function and decreases the formation of micronuclei, suggesting that a decrease of mtDNA and DNA damage may contribute to the decrease of STING in C-176 treated cells and kidneys, which needs be further investigated.

Over the past decade, understanding the role of inflammatory responses in the pathogenesis of ADPKD has been a key area of focus [42]. Accumulating evidence indicates that interstitial macrophages contribute to cyst growth in both human ADPKD and rodent cystic models [4,6]. Recently, macrophage reprogramming and immunometabolism have been linked to the activation of STING [43]. Macrophages are typically categorized into classically activated (M1) and alternatively activated (M2) subtypes. M1 macrophages are characterized by an inflammatory profile, marked by the production of pro-inflammatory cytokines such as TNF-α, MCP-1, IL-1β, and nitric oxide (NO). M2 macrophages exhibit an anti-inflammatory phenotype, characterized by the secretion of cytokines such as IL-10 and increased mitochondrial oxidative phosphorylation (OXPHOS), which contributes to an immunosuppressive response [44]. STING is involved in the reprogramming of macrophages, shifting them towards an inflammatory phenotype [45]. The recruitment of M2-like macrophages was increased in interstitial and pericystic regions in ADPKD kidneys [3,6], which were reported to be mediated by inflammatory cytokines such as TNF-α and MCP-1 [6]. The depletion of macrophages can delay cyst growth and enhance renal function in *Pkd1* mutant mouse kidneys [6]. In this study, we show an alternative mechanism of STING mediating the recruitment of macrophages, which may be regulated by STING/NF-κB signaling to increase the expression of TNF-α, and MCP-1 in *Pkd1* mutant mouse and human ADPKD kidneys.

Emerging evidence suggests that the STING-dependent signaling network is pivotal in regulating various pathways of cellular death, including apoptosis, necroptosis, pyroptosis, ferroptosis, mitotic cell death, and immunogenic cell death [46]. The mechanism of STING-mediated apoptosis is intricate and appears to be closely related to ER stress [47]. The activation of STING in response to endoplasmic reticulum (ER) stress induces the activation of interferon regulatory factor 3 (IRF3). This can enhance mitochondrial outer membrane permeabilization (MOMP) mediated by BAX/BAK1 through the formation of an IRF3-BAX complex or by inhibiting BCL2L1, ultimately leading to caspase activation and apoptosis [48]. Increased apoptosis, in turn, can further regulate the STING-IRF3 pathway by releasing mtDNA or activating caspase 3-mediated cleavage of cGAS or IRF3. Additionally, STING can induce necroptosis by cooperating with the downstream type I interferon-mediated expression of mixed lineage kinase domain-like protein (MLKL) and tumor necrosis factor (TNF)-induced phosphorylation of receptor-interacting protein kinases 1 and 3 (RIPK1 and RIPK3) [46]. On the other hand, STING activation has been shown to attenuate tumor cell killing and promote tolerogenic responses during Lewis lung carcinoma (LLC) growth by stimulating local indoleamine 2,3 dioxygenase (IDO) activity and decreasing CD8^+^ T cell effector functions during tumorigenesis [49]. As an important mediator of cells death, p53 plays a fundamental role in mediating DNA damage response (DDR), promoting cell cycle arrest, senescence, and apoptosis [50]. A recent study demonstrates that p53 interacts with the cGAS/STING cytosolic DNA sensing pathway to facilitate tumor suppression. Specifically, p53 promotes the degradation of the cytosolic DNA exonuclease TREX1, leading to the accumulation of cytoplasmic DNA and the subsequent activation of the cGAS/STING pathway. The absence of cGAS or STING impairs the tumor suppressive function of p53 [51]. A key point of this study is that the inhibition of STING with C-176 induced a p53-dependent apoptosis in *Pkd1* mutant renal epithelial cells. Unexpectedly, we also found that the inhibition of STING with C-176 resulted in an increase of p53 protein in *Pkd1* mutant renal epithelial cells and tissues, suggesting that the upregulation of p53 should not contribute to STING activation but just cystic renal epithelial cell death in *Pkd1* mutant mouse kidneys treated with STING inhibitor. However, how targeting STING results in the upregulation of p53 needs be further investigated.

Another key feature of this study shows that targeting STING decreases renal fibrosis in *Pkd1* mutant mouse kidneys through regulating the expression of fibrotic markers. Renal fibrosis is frequently observed in ADPKD kidneys and contributes to end-stage renal disease (ESRD) associated with PKD mutations [1]. Various inflammatory factors, including TNFα, MIF, and TGF-β, have been reported to promote renal fibrosis in ADPKD [26,52], in which TGF-β has been identified as the most important regulator of renal fibrosis [53]. Mitochondrial dysfunction and inflammation have been implicated in renal fibrosis in kidney diseases [54]. A recent study reveals that the accumulation of mitochondrial DNA (mtDNA) in the cytosol of tubular epithelial cells activates the cGAS-STING pathway, initiating an immune response that contributes to fibrotic changes [36]. Our results further support that STING activation may integrate these two mechanisms in the regulation of renal fibrosis in ADPKD by a reduction in extracellular matrix deposition and a decrease in the expression of fibrotic marker genes. However, how STING regulates the expression of fibrotic markers needs to be investigated.

In conclusion, this study identifies that STING is a key regulator of cyst progression in ADPKD, in that genotoxic stress stemming from *Pkd1* gene mutation contributes to dysregulated STING mediated cellular processes, including inflammation, microphage recruitment, apoptosis, and fibrosis. Our results underscore a potential therapeutic strategy of inhibition of STING activation with pharmacological agents, such as C-176, for the treatment of ADPKD.

## Data Availability

Data for this work are available from the corresponding author on reasonable request.

## Supplementary Materials

The following supporting information can be downloaded at: https://www.mdpi.com/article/10.3390/biom14101215/s1, Table S1: Primers used for quantitative real time PCR. File S1: Original Western blot images.
