# Supplementary material for: STING Promotes the Progression of ADPKD by Regulating Mitochondrial Function, Inflammation, Fibrosis, and Apoptosis
Source: Biomolecules. 2024 Sep 26;14(10):1215. doi: 10.3390/biom14101215 (PMC11505933; doi:10.3390/biom14101215)

Figure 1A

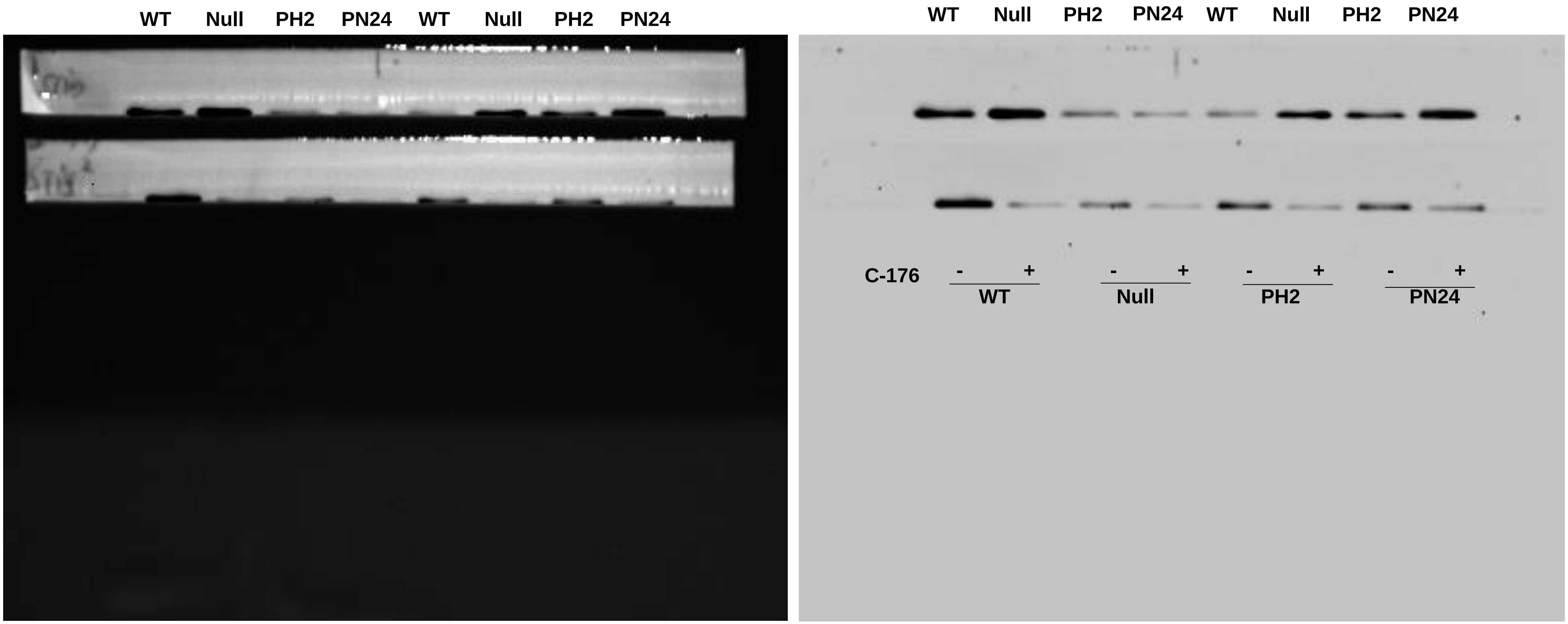
WT Null PH2 PN24 WT Null PH2 PN24
WT Null PH2 PN24 WT Null PH2 PN24
C-176 - + - + - + - +
WT Null PH2 PN24

Figure 1A

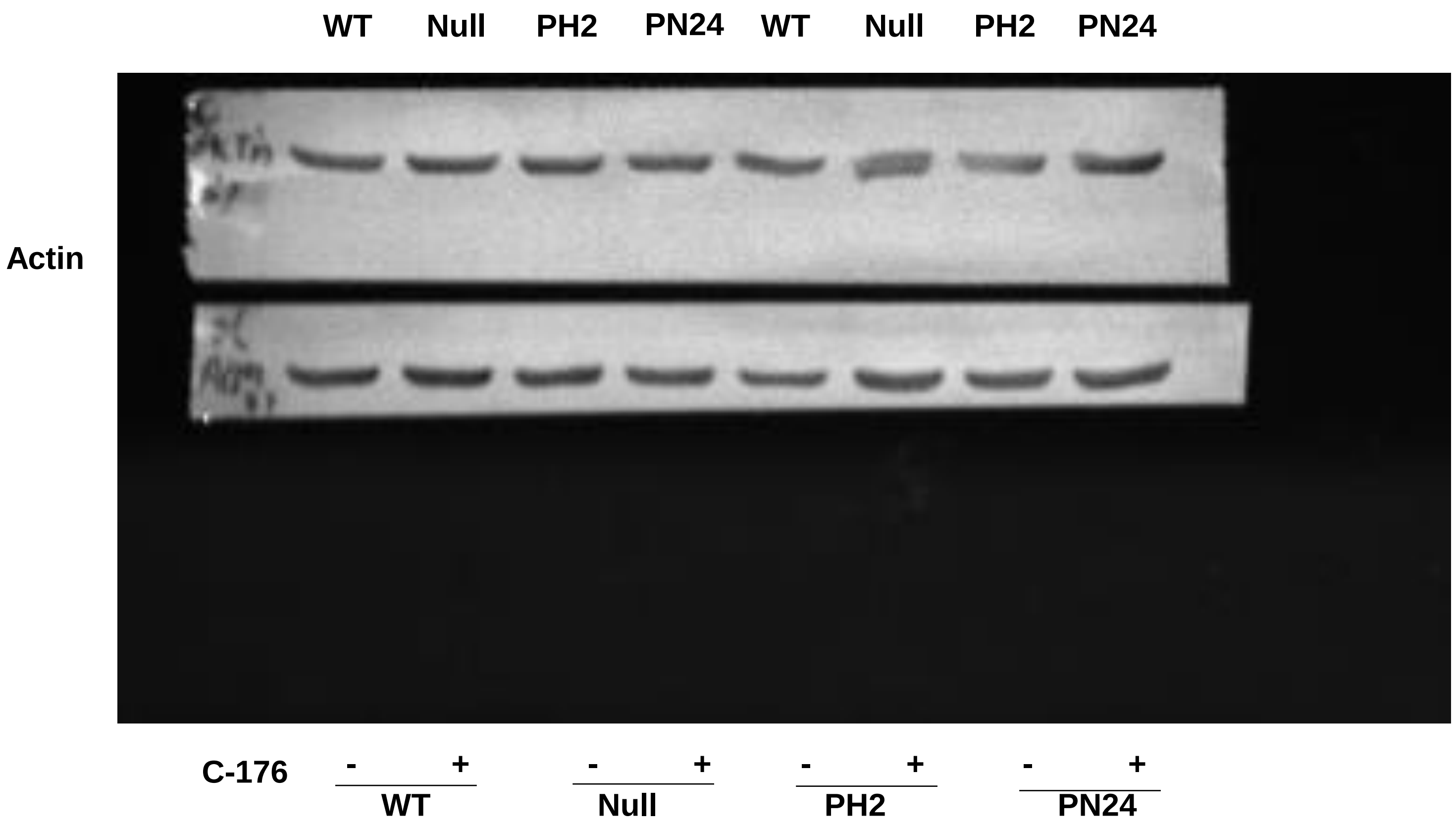

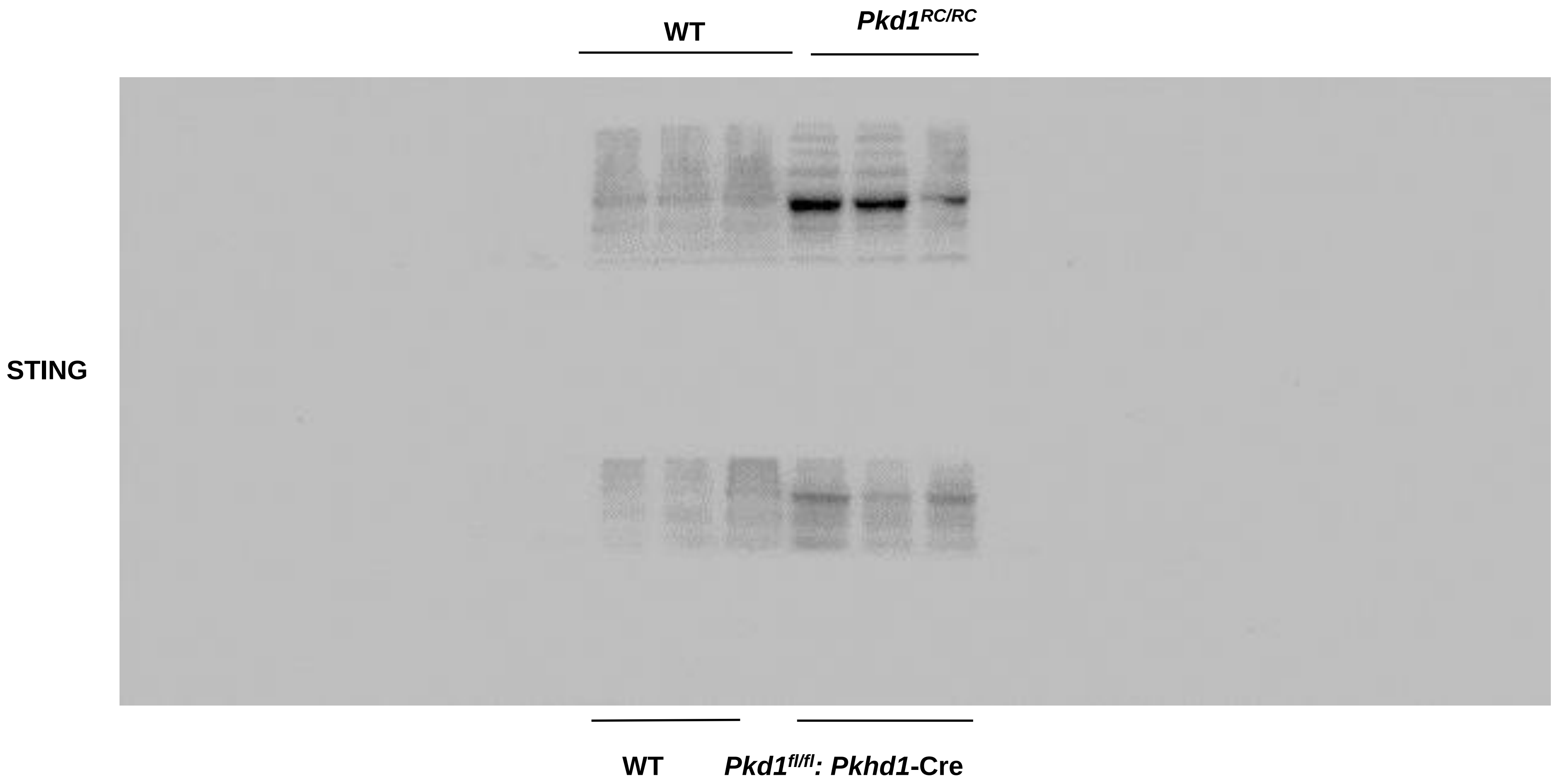
WT
*Pkd1^RC/RC^*
STING
WT
Pkd1^fl/fl^: Pkhd1-Cre

Figure 1B

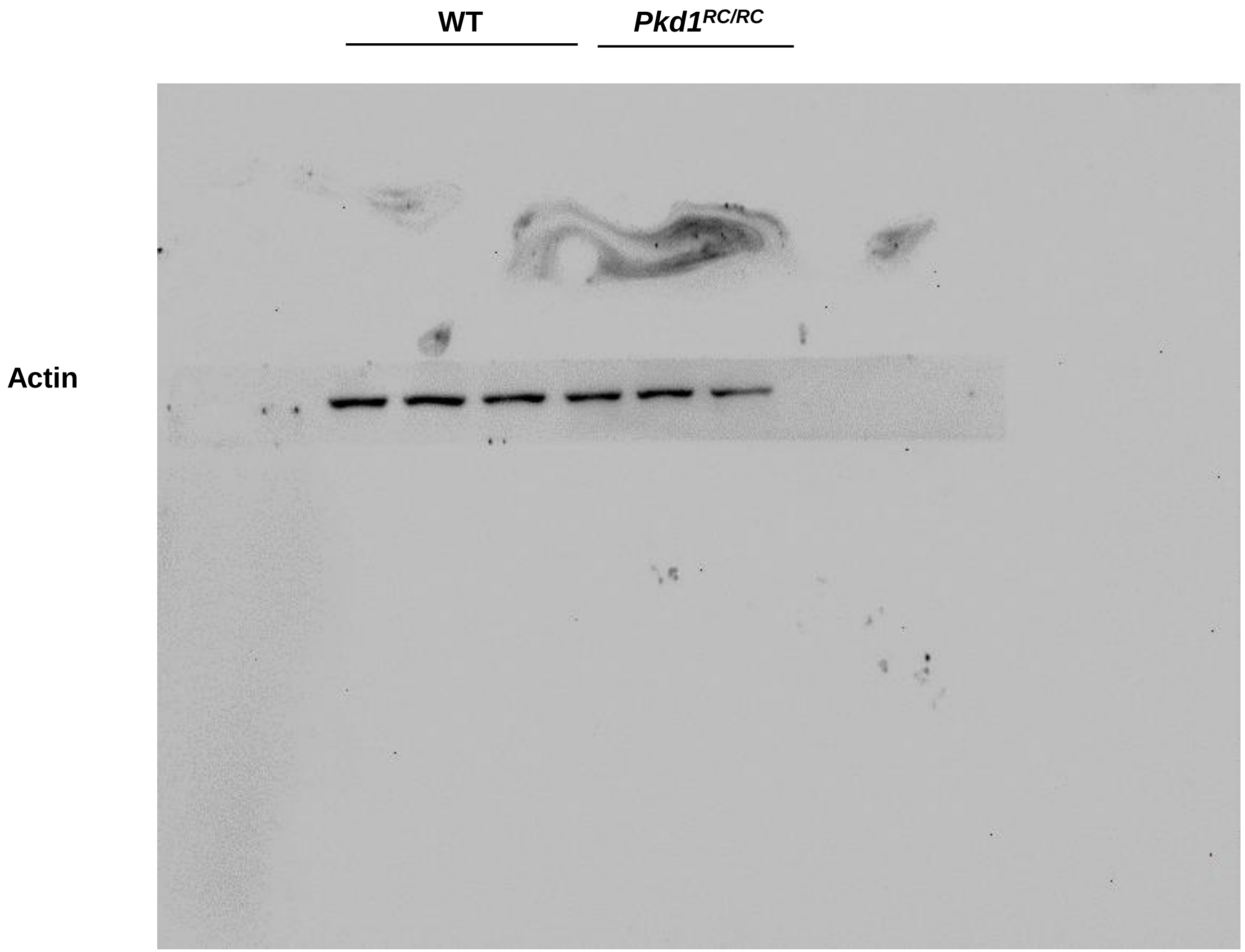

WT

Pkd1$^{fl/fl}$: Pkhd1-Cre

Actin

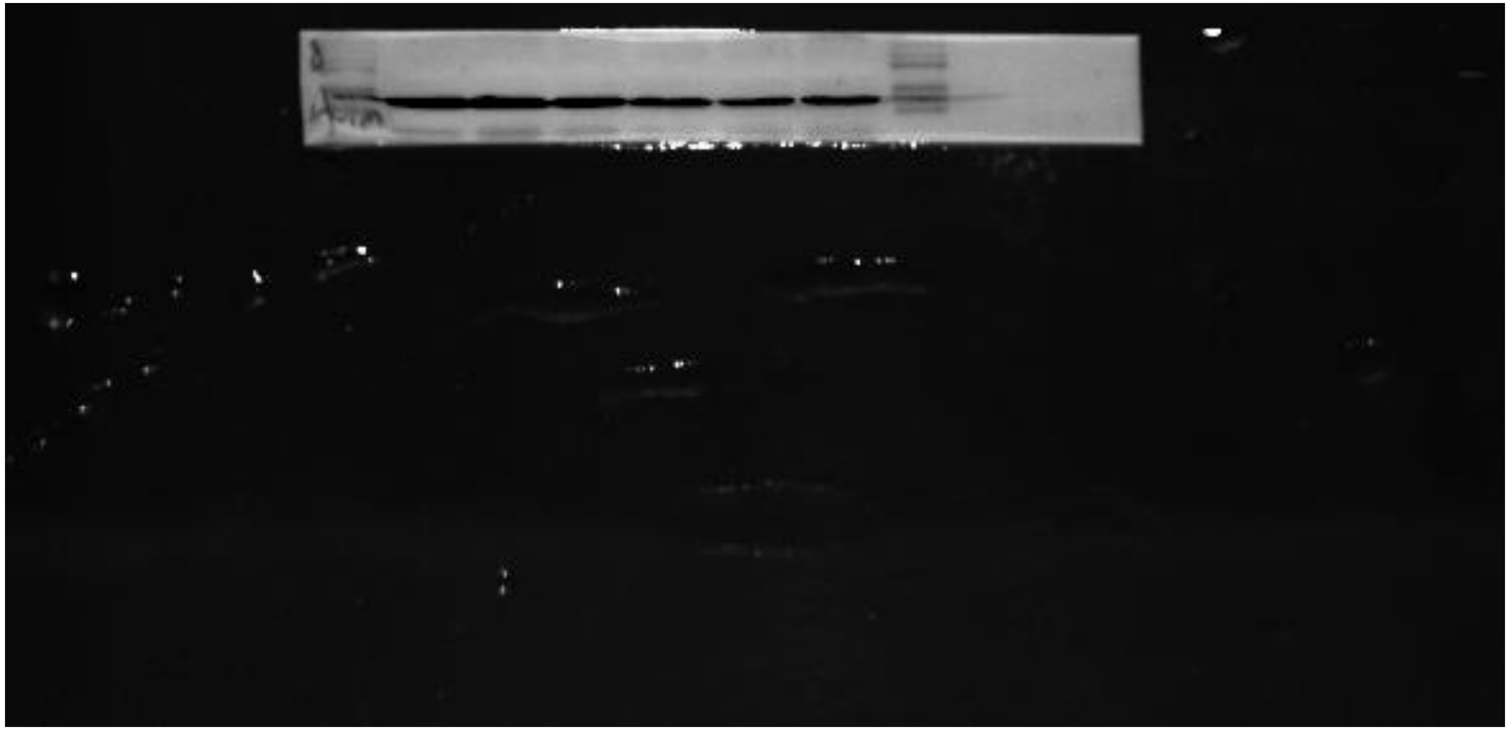

Figure 1G

PH2 PN24 WT Null

cGAS

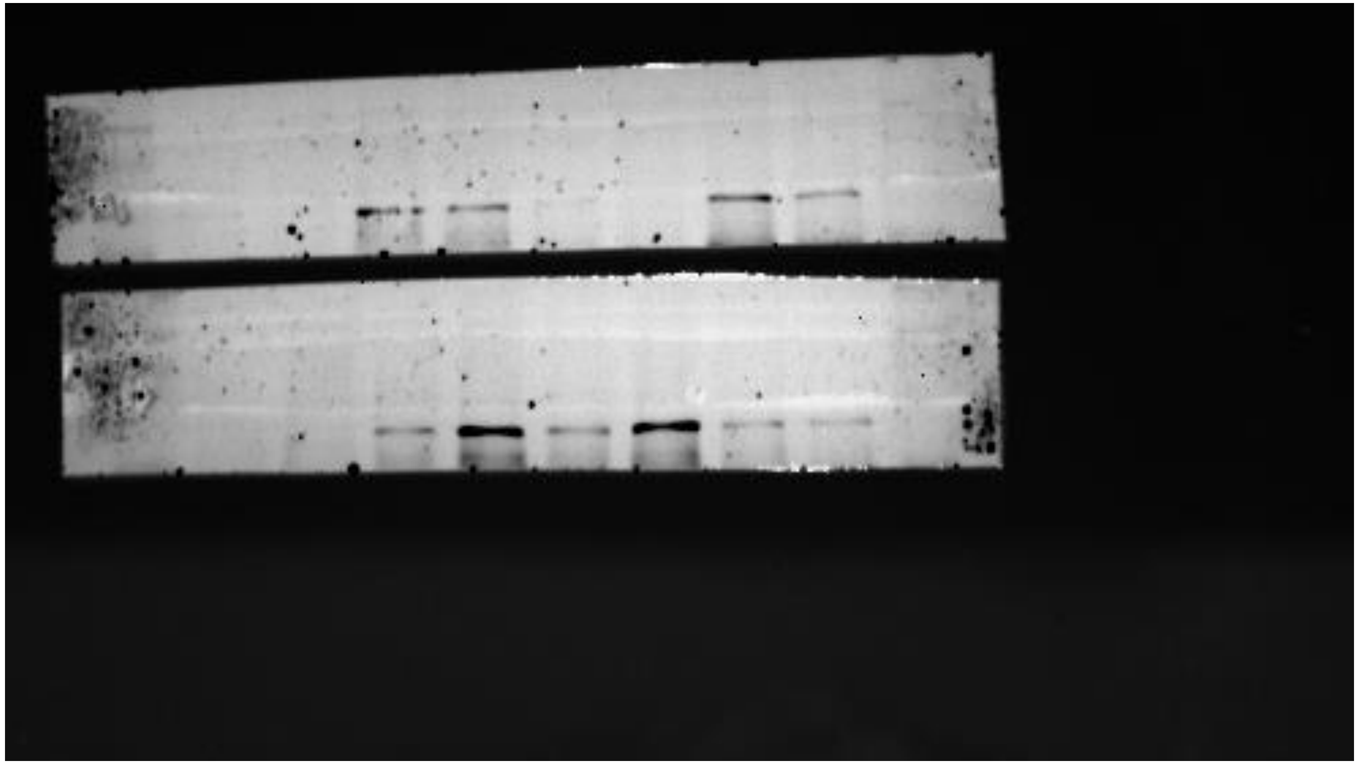

PH2 PN24 WT Null PH2 PN24 WT Null

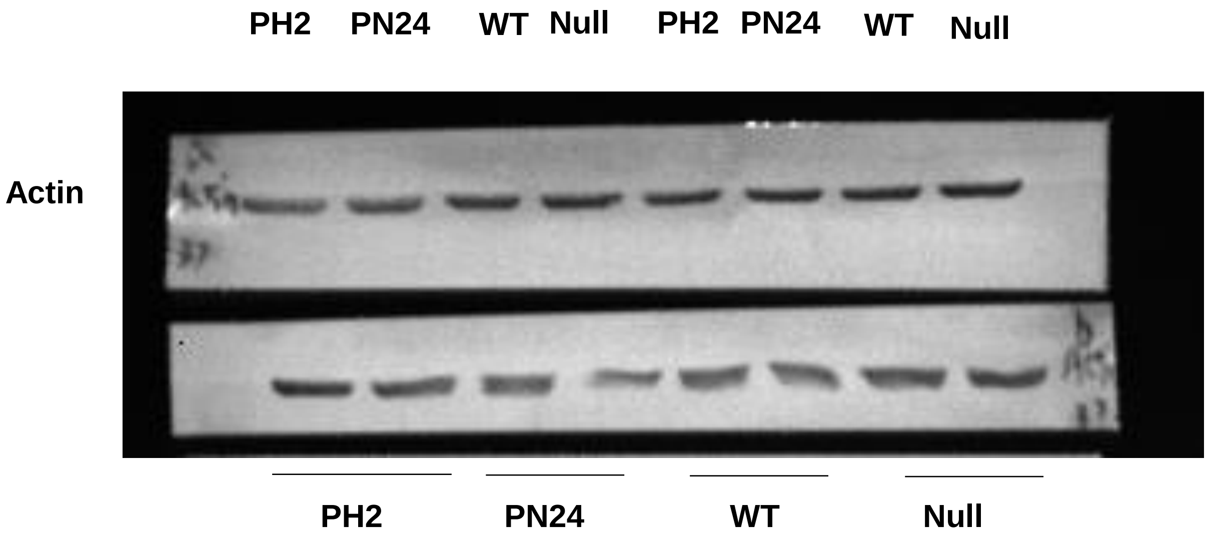

Figure 1G

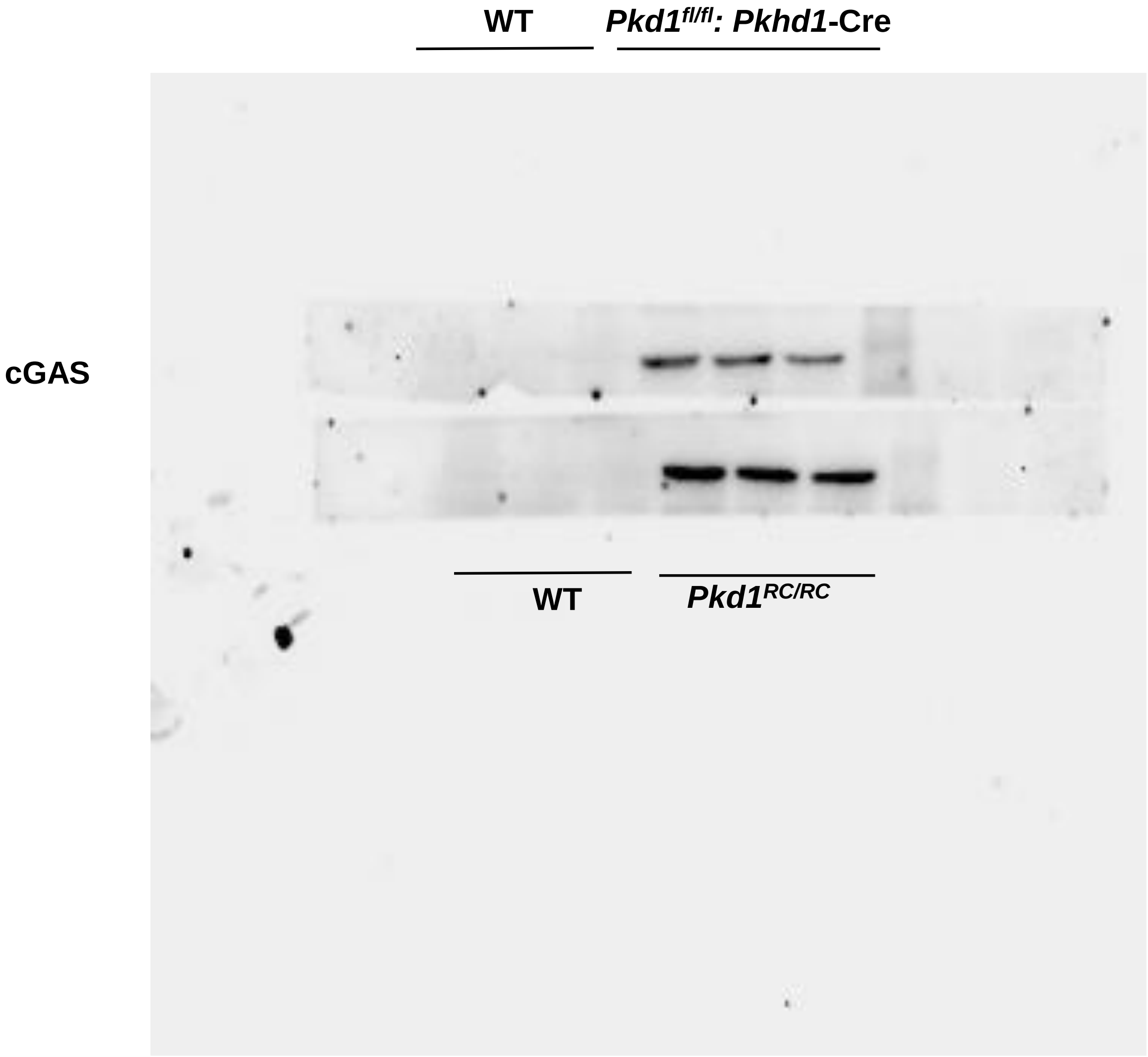
WT
Pkd1fl/fl: Pkhd1-Cre
cGAS
WT
Pkd1RC/RC

Figure 1H

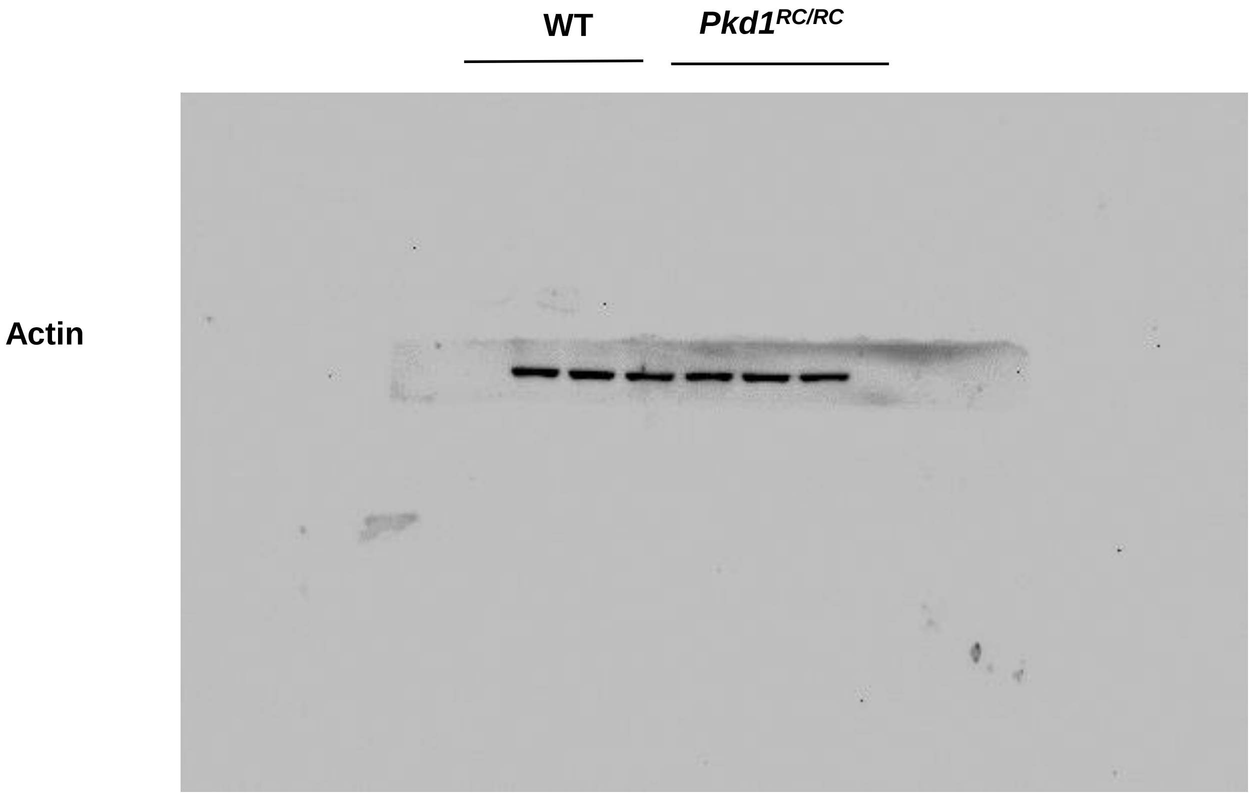

Figure 1I

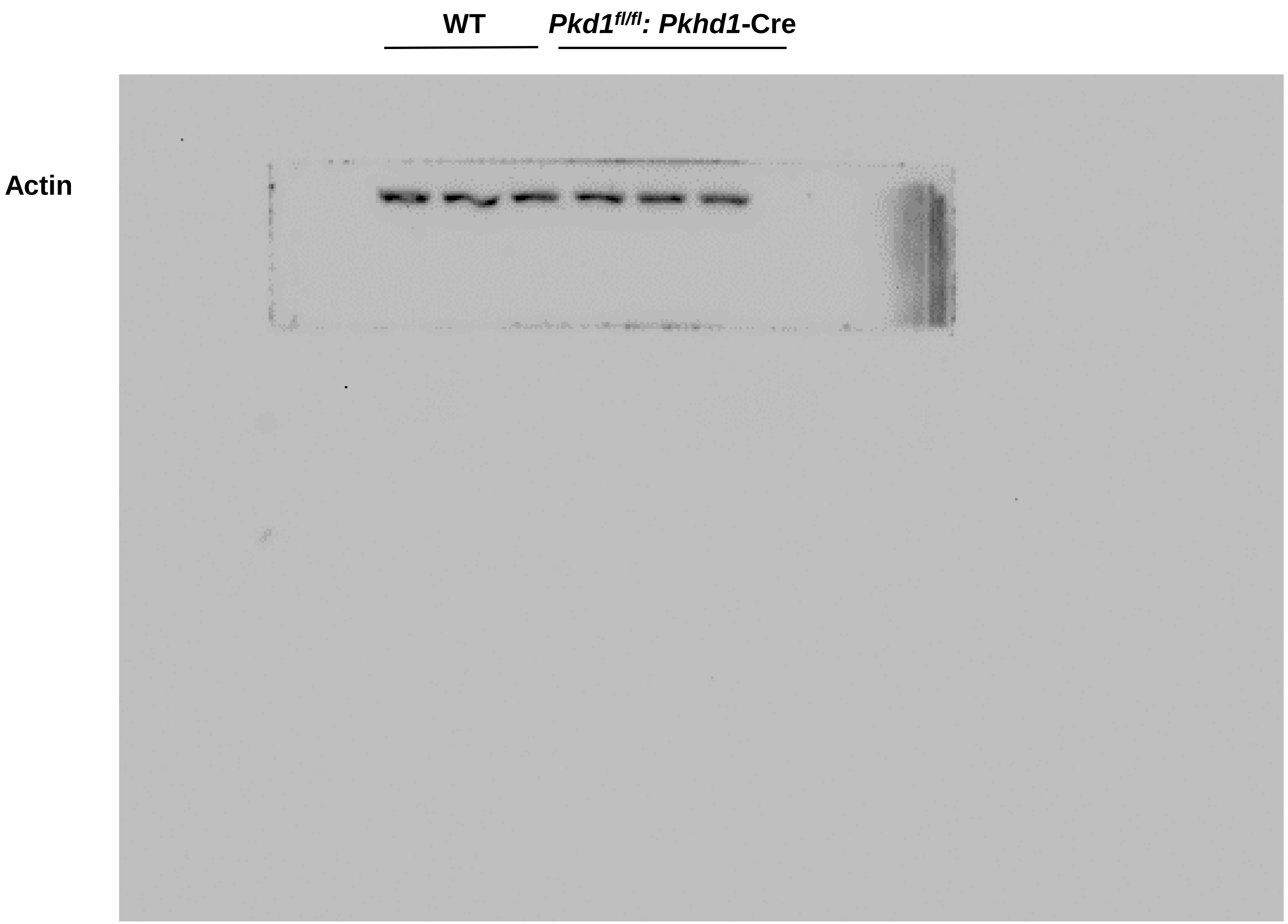

Figure 4A,B

WT | *Pkd1$^{RC/RC}$*

C-176 - - - - - - + + +

STING

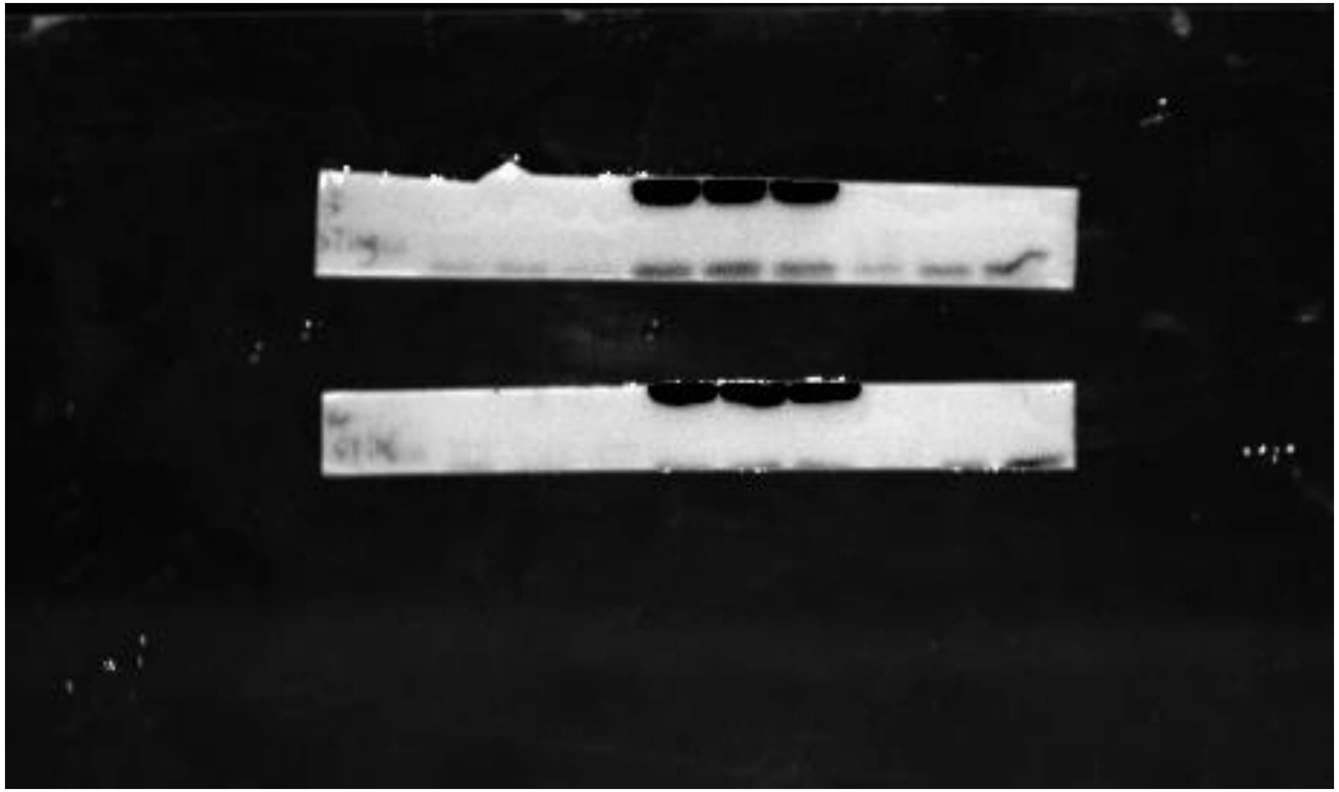

C-176 - - - - - - + + +

WT | *Pkd1$^{fl/fl}$: Pkhd1-Cre*

Figure 4A

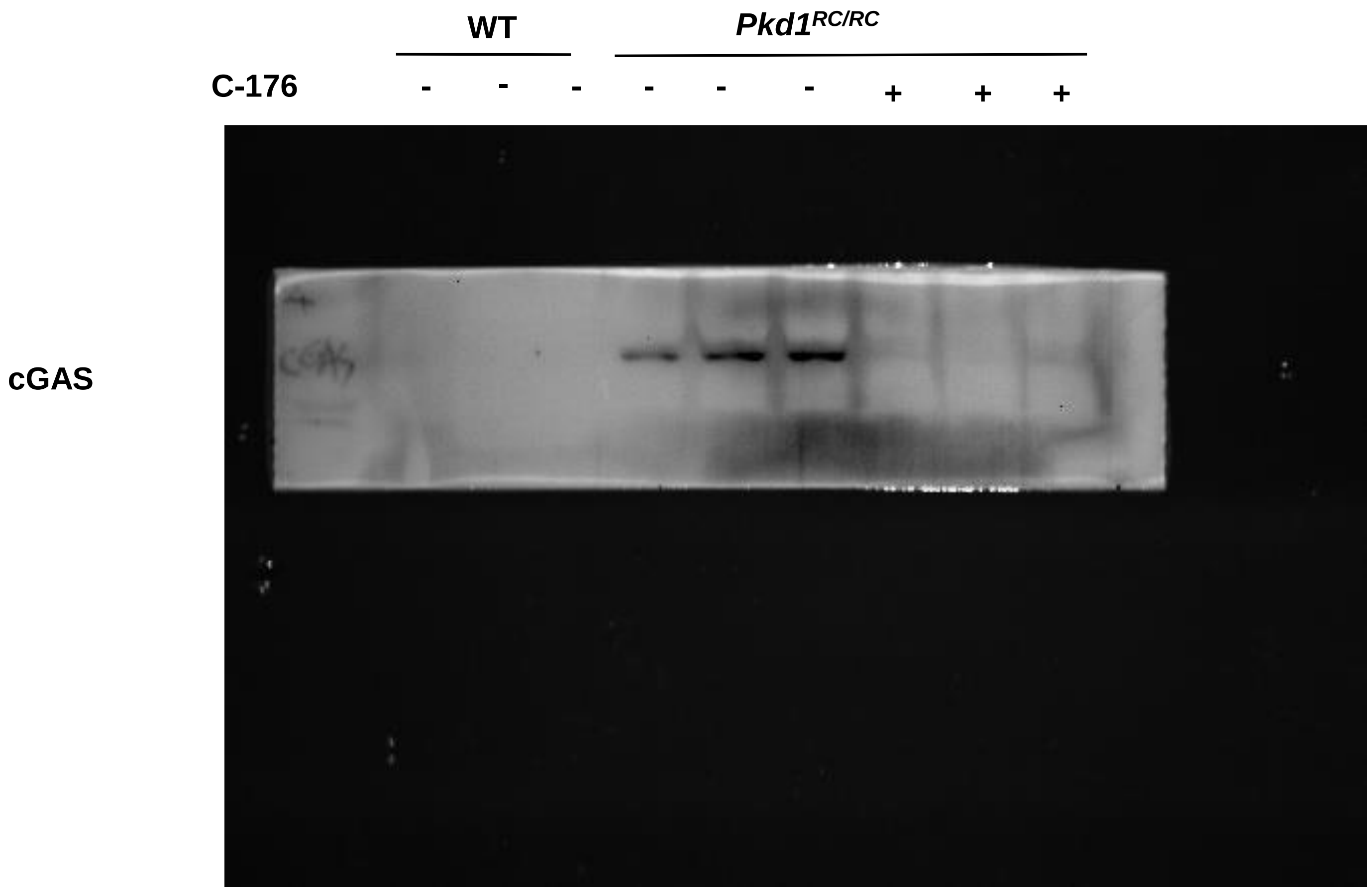

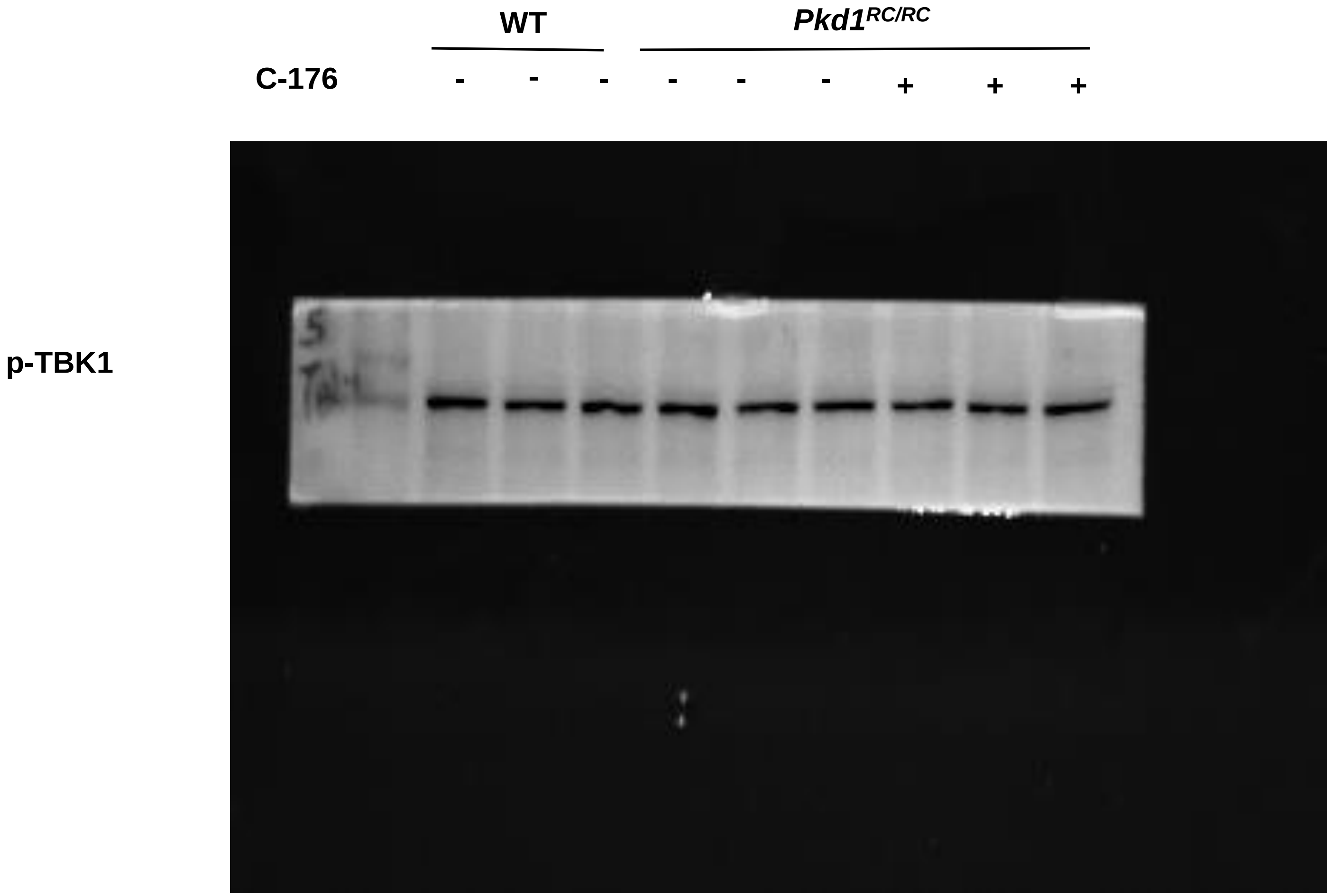
WT
Pkd1RC/RC
C-176
- - - - - - + + +
p-TBK1

Figure 4A

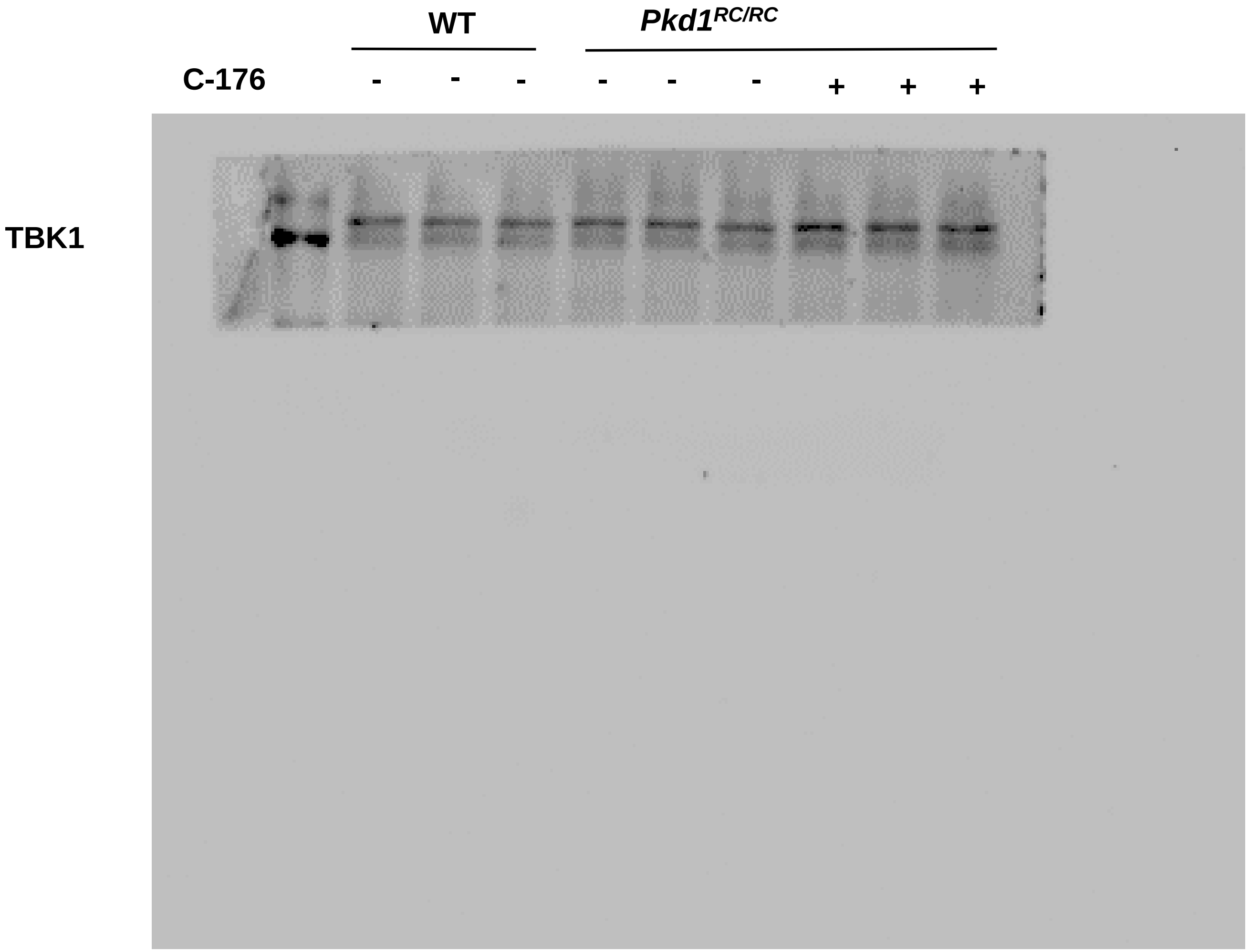

Figure 4A

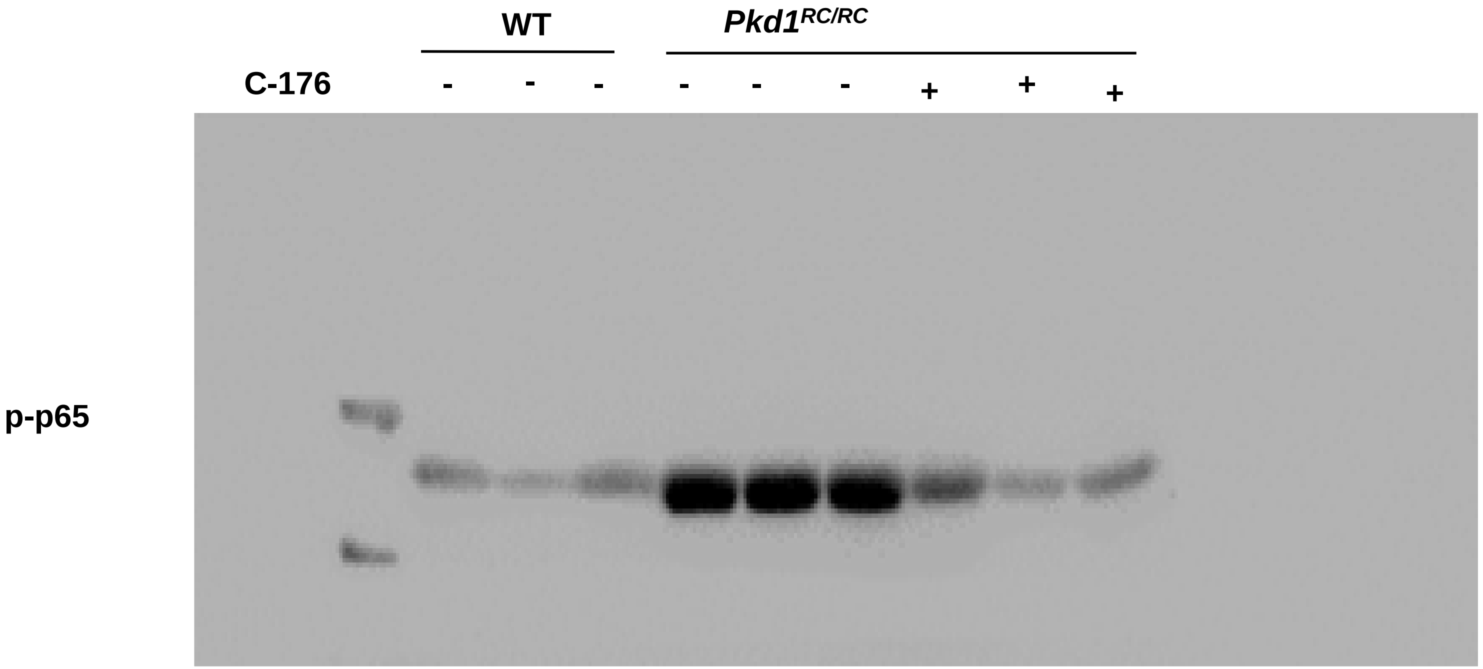

Figure 4A

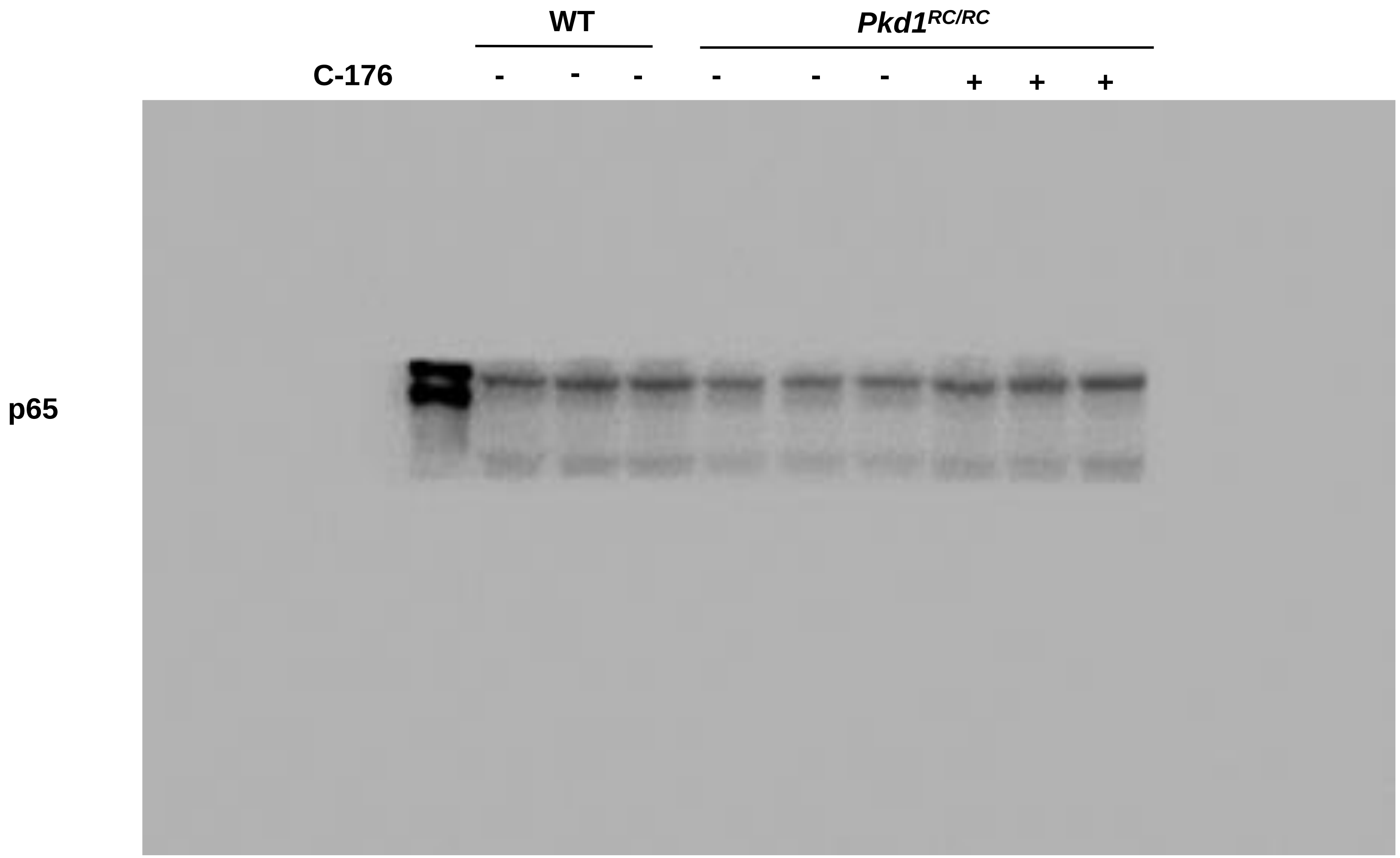

Figure 4A

	WT			*Pkd1$^{RC/RC}$*					
C-176	-	-	-	-	-	-	+	+	+

Actin

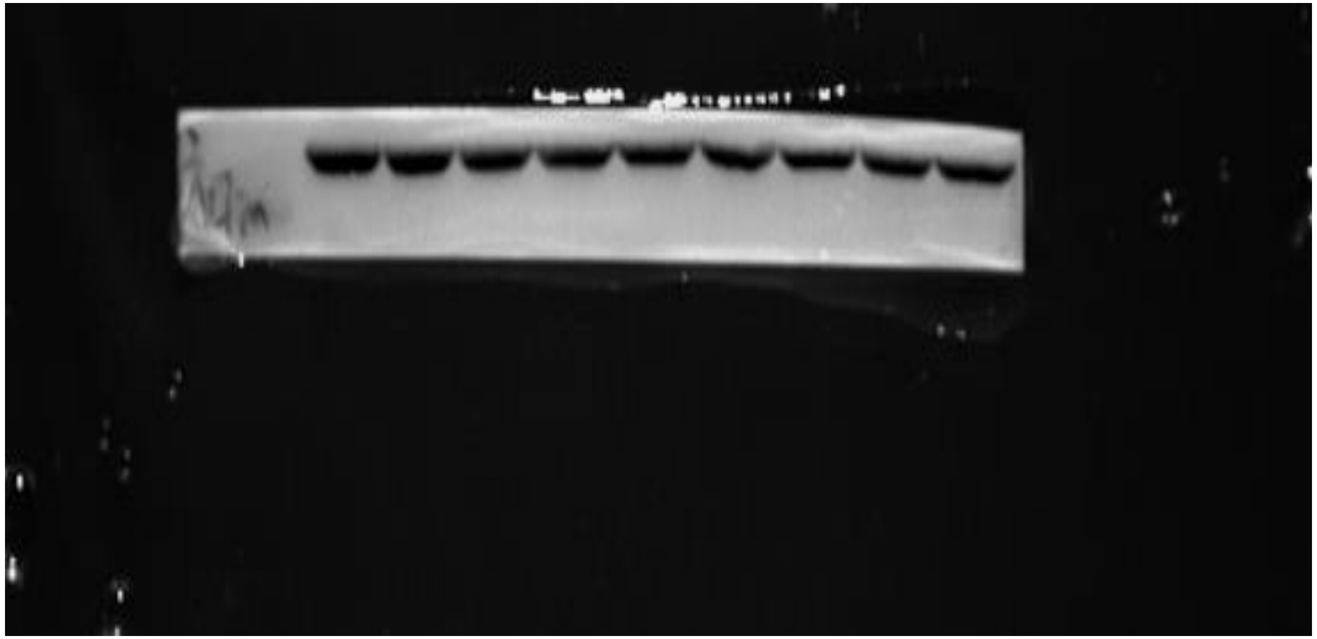

Figure 4B

STING

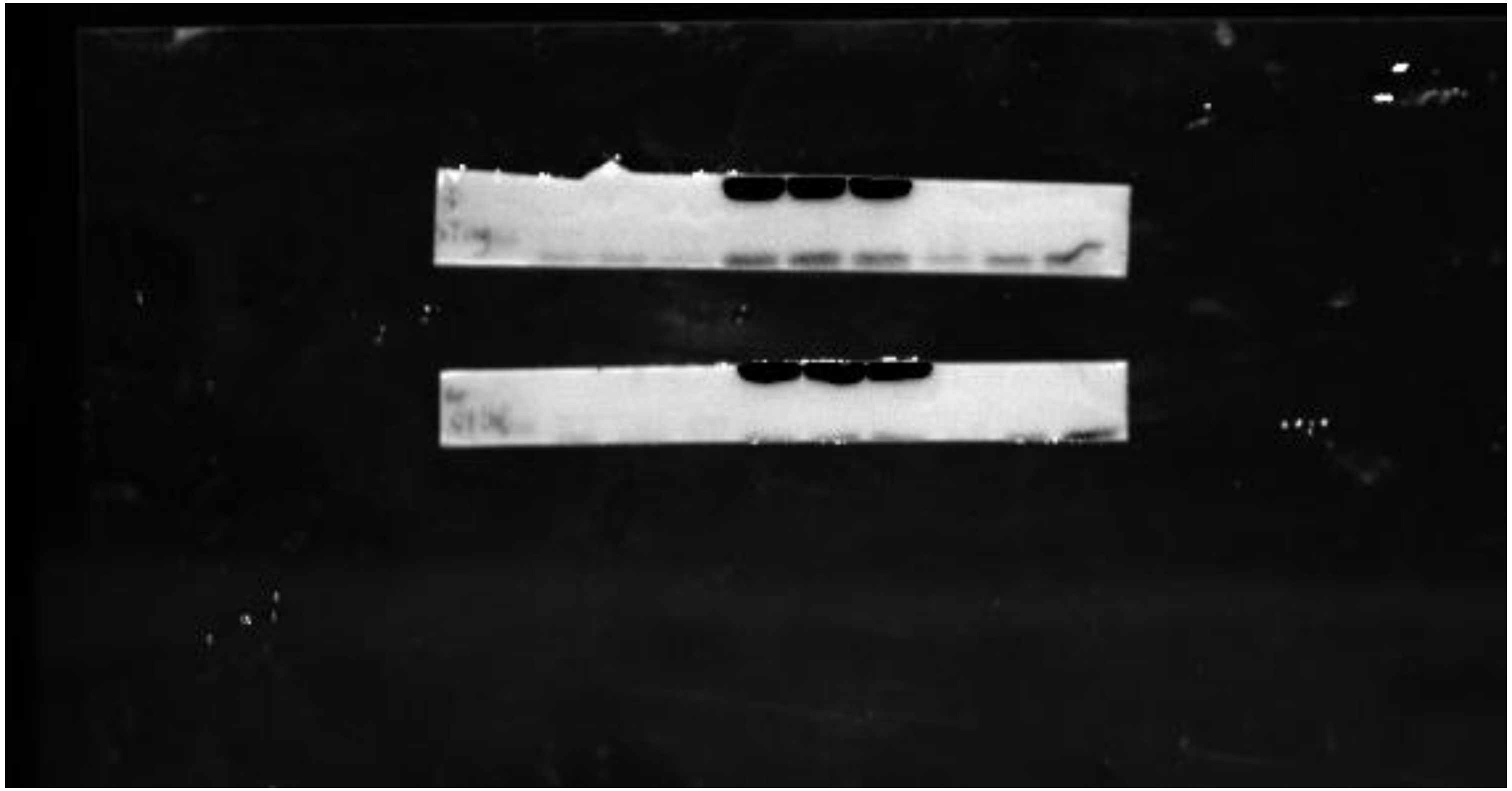

C-176 - - - - - - + + +

WT

Pkd1$^{fl/fl}$: Pkhd1-Cre

Figure 4B

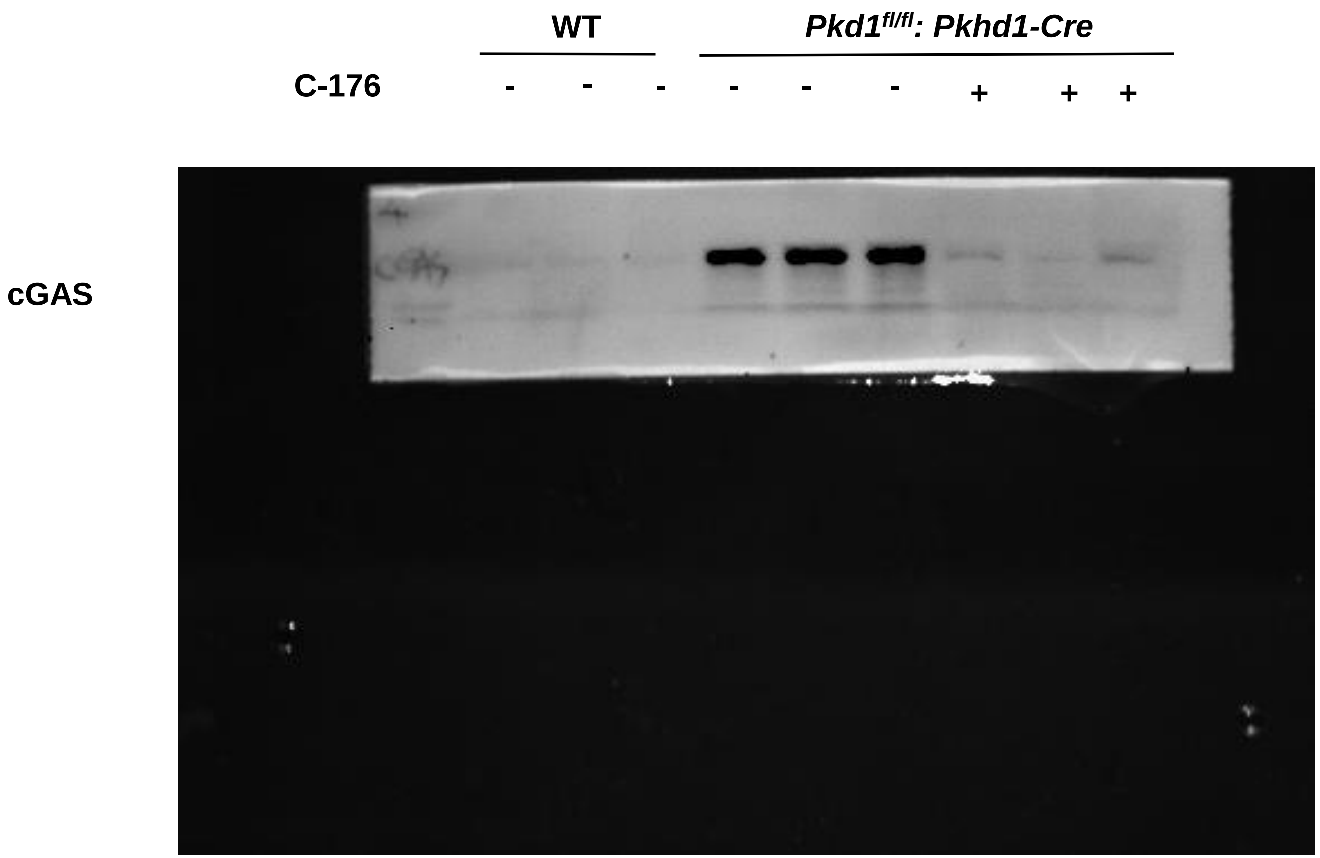

Figure 4B

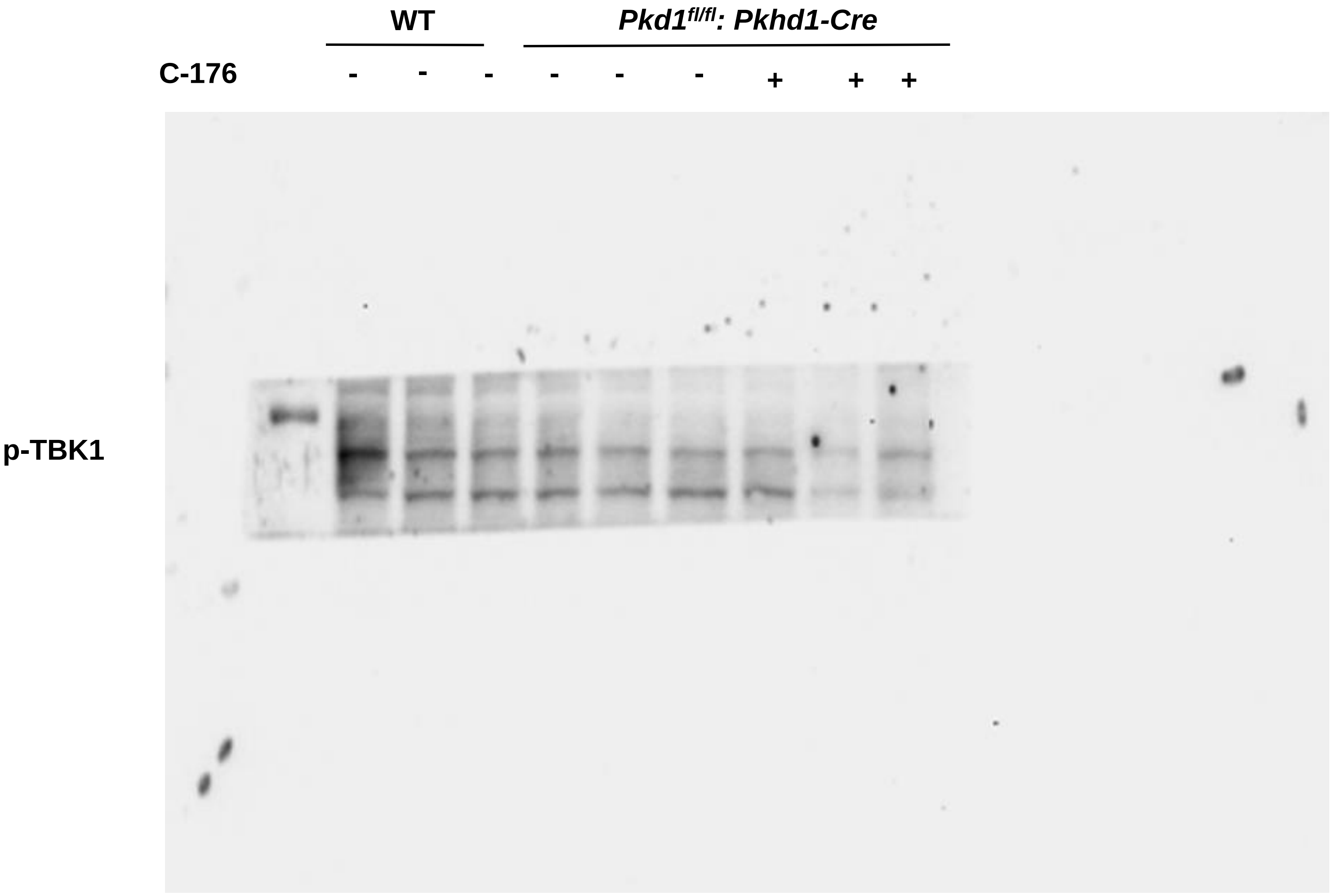

Figure 4B

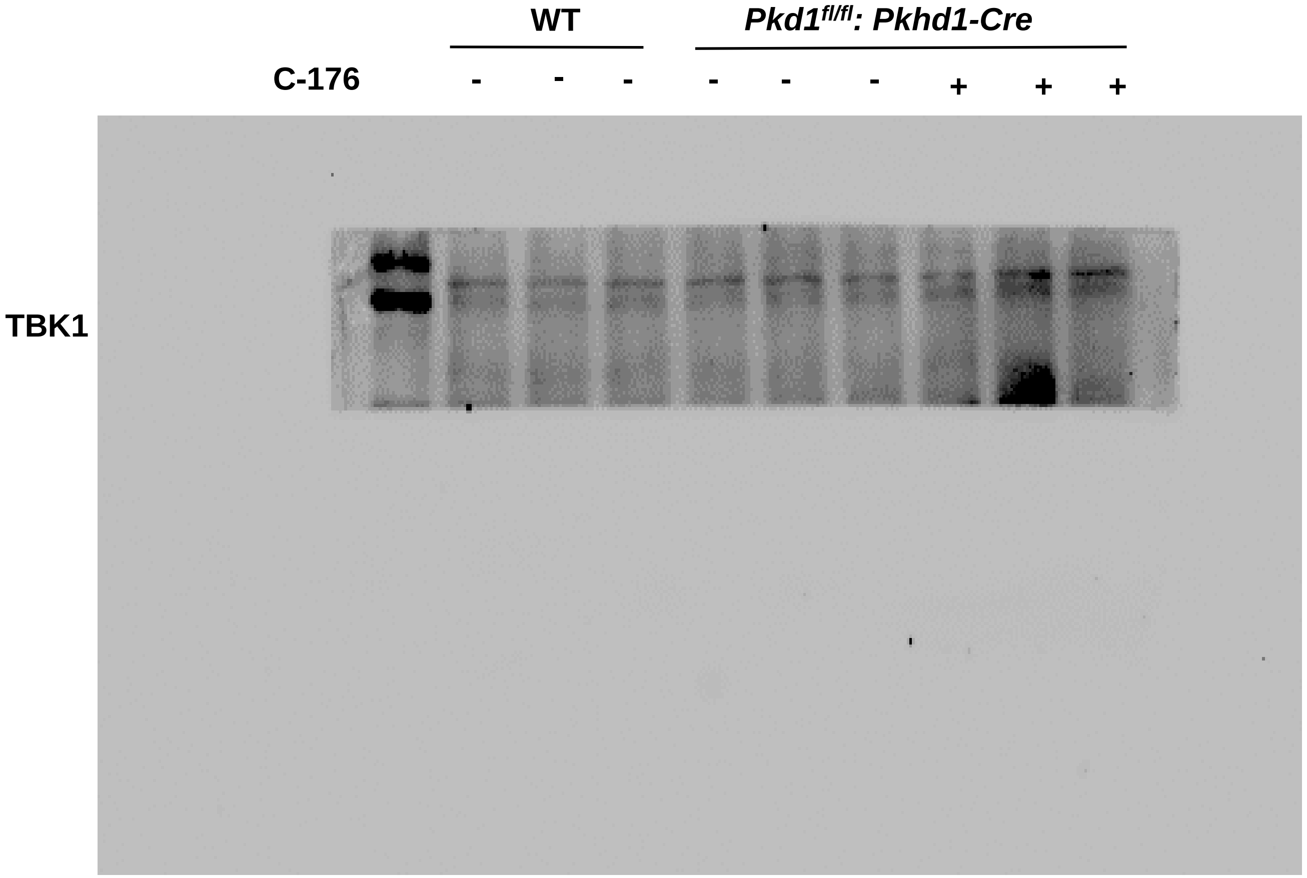

Figure 4B

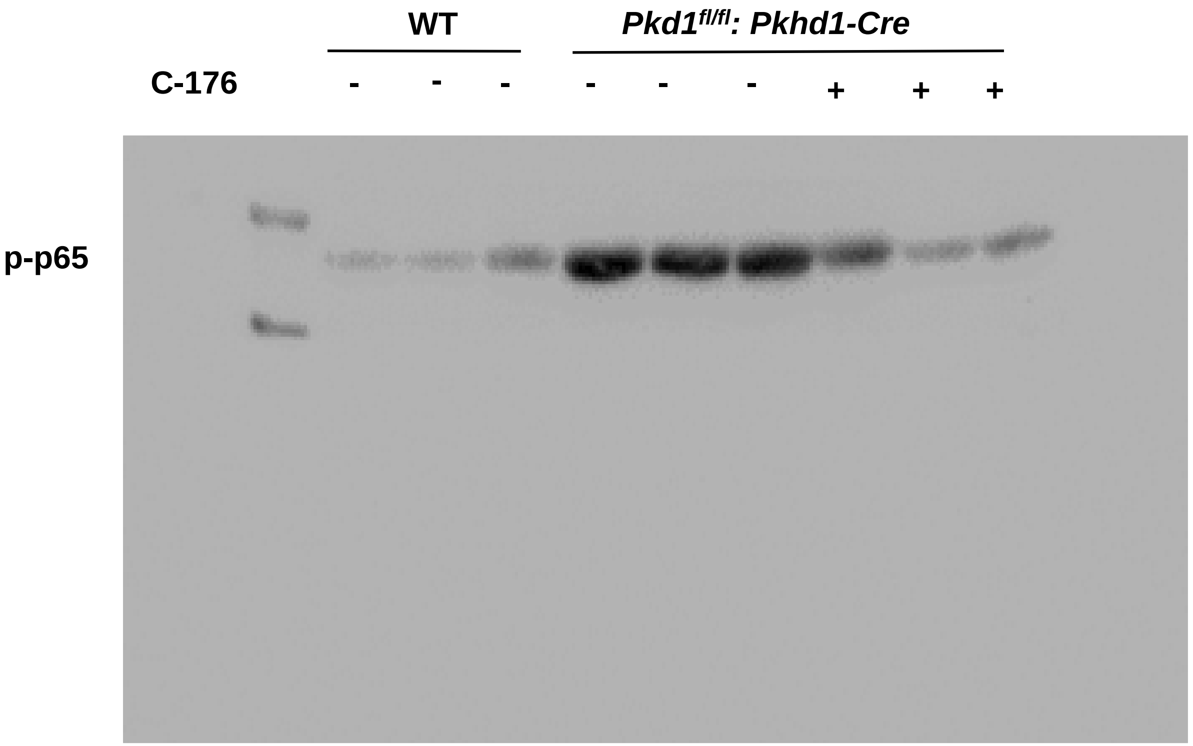

Figure 4B

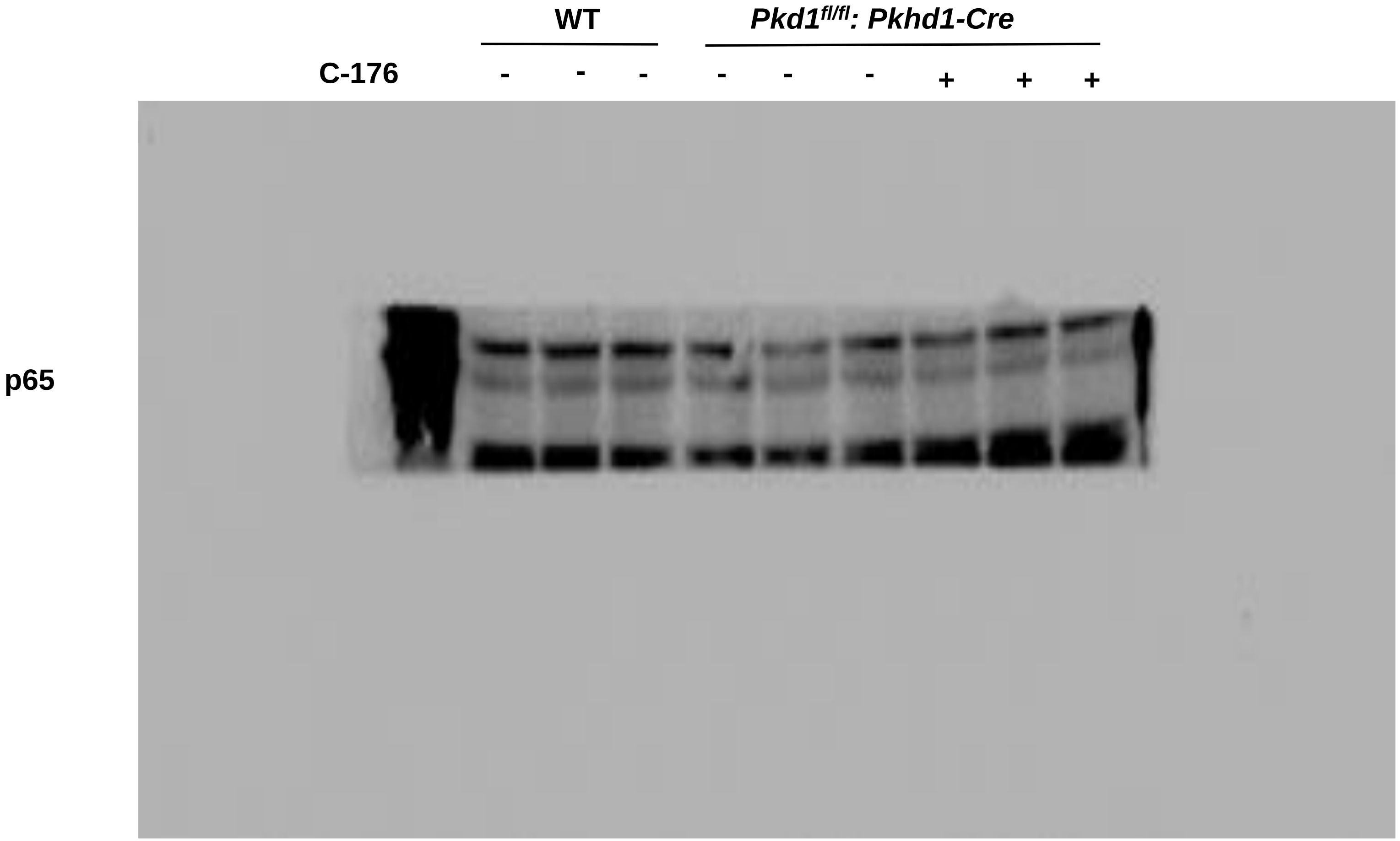

Figure 4B

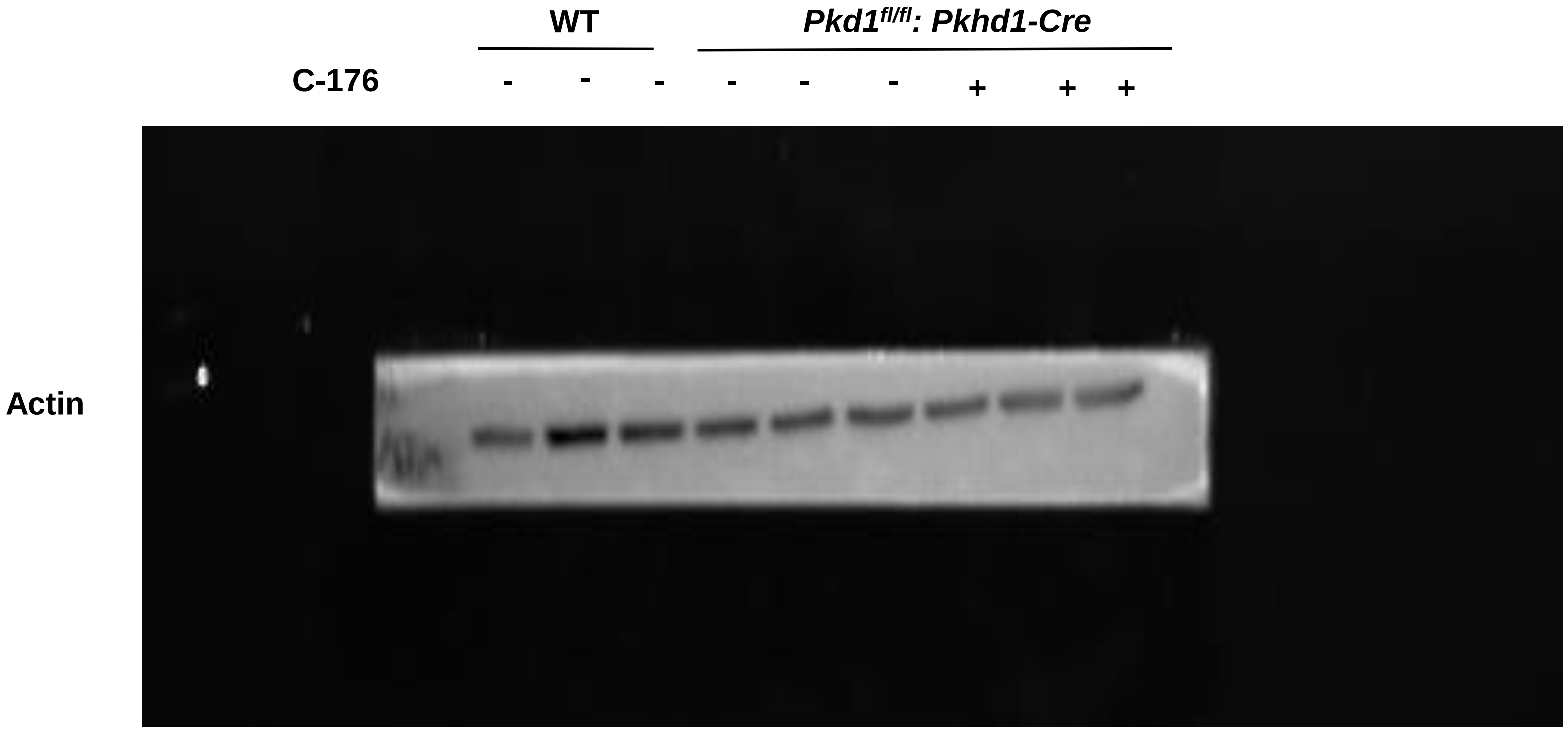

Figure 4C

C-176 (uM)

DMSO 0.01 0.1 1 5

STING

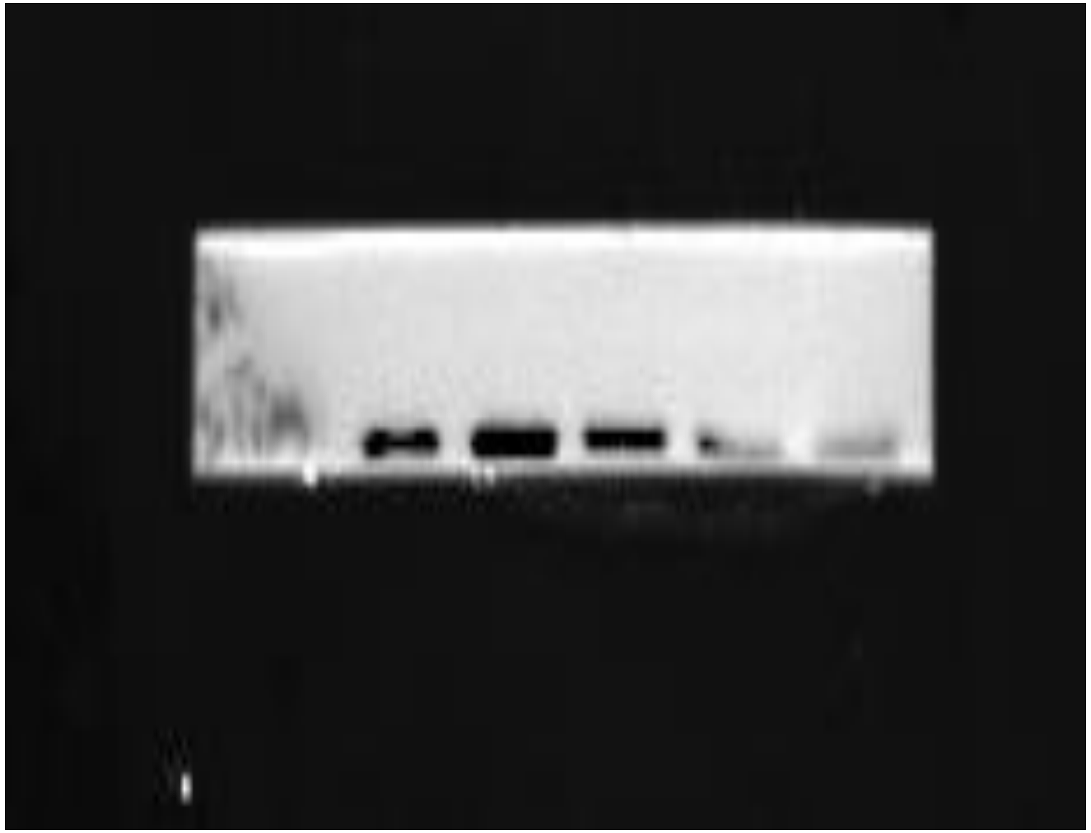

Figure 4C

C-176 (uM)

DMSO 0.01 0.1 1 5

p-p65

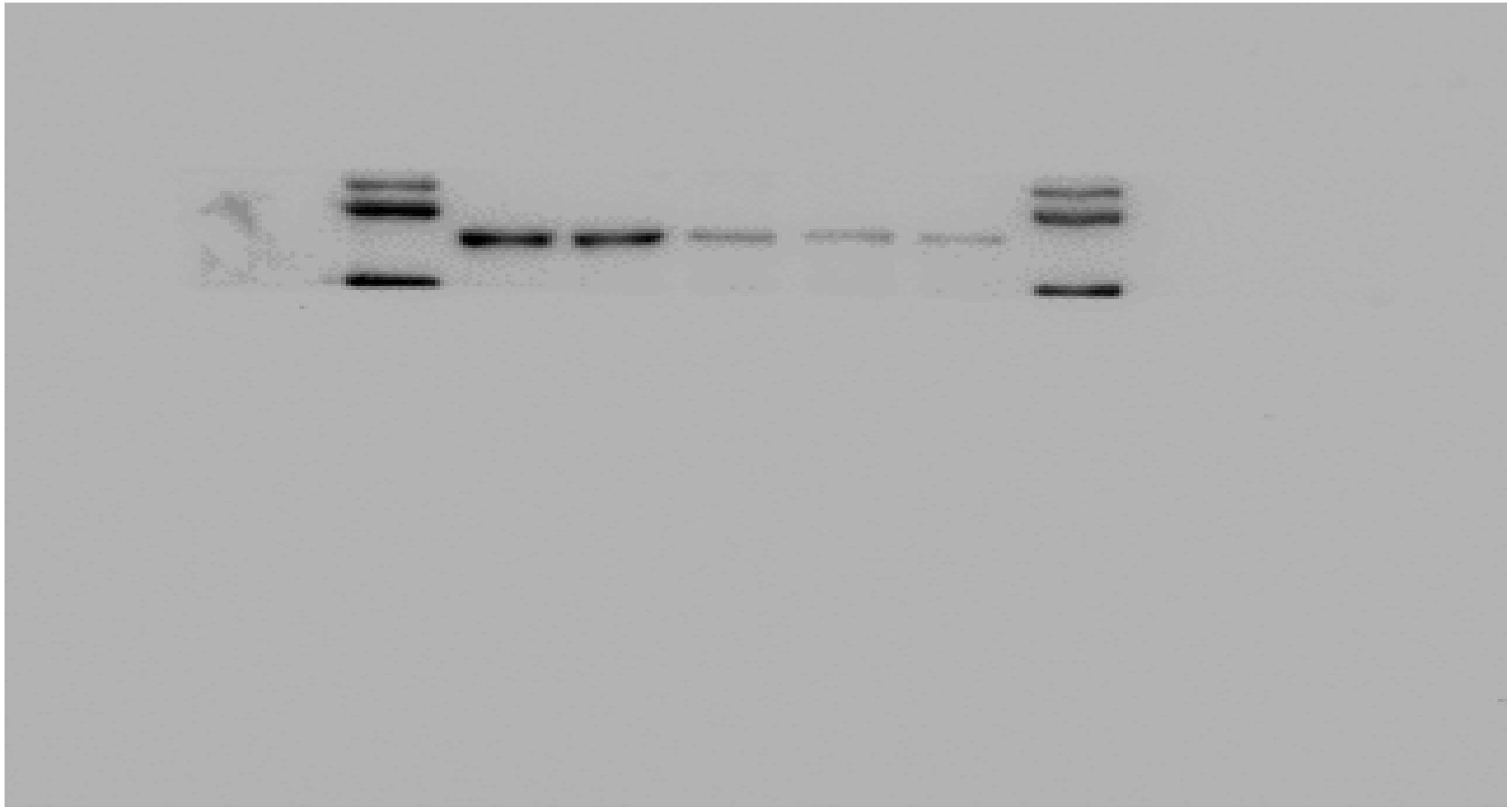

Figure 4C

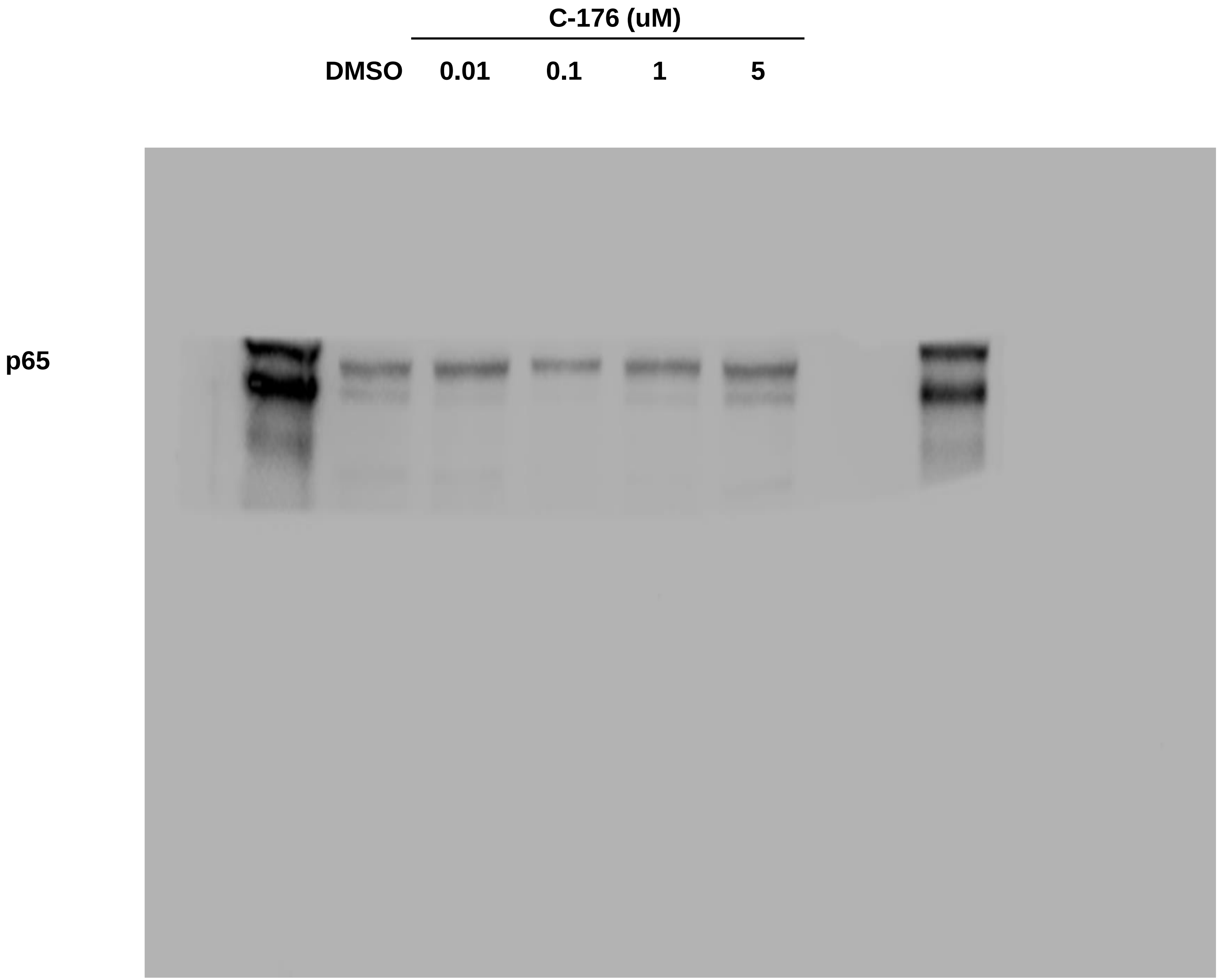

Figure 4C

C-176 (uM)

DMSO 0.01 0.1 1 5

Actin

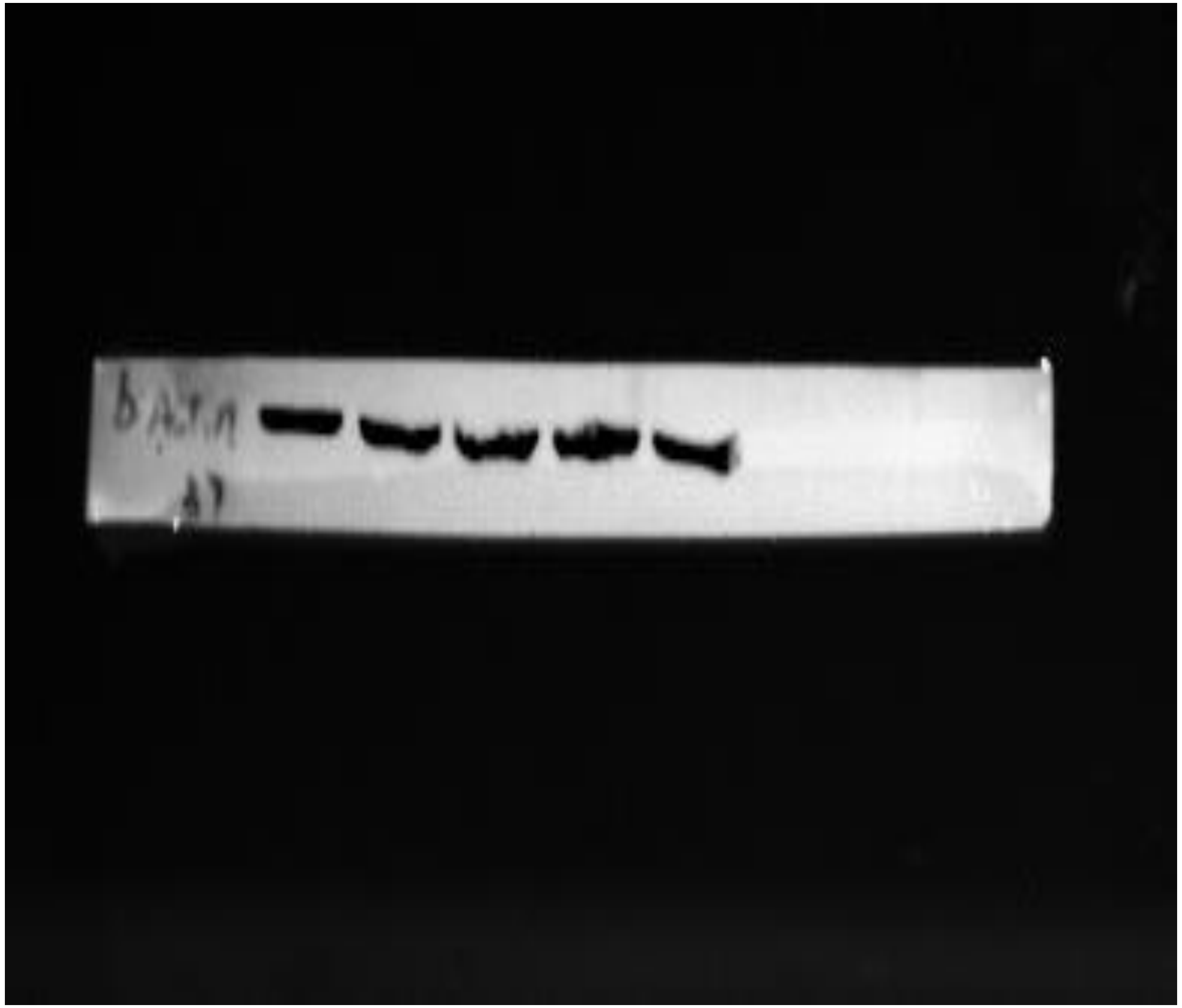

Figure 4D

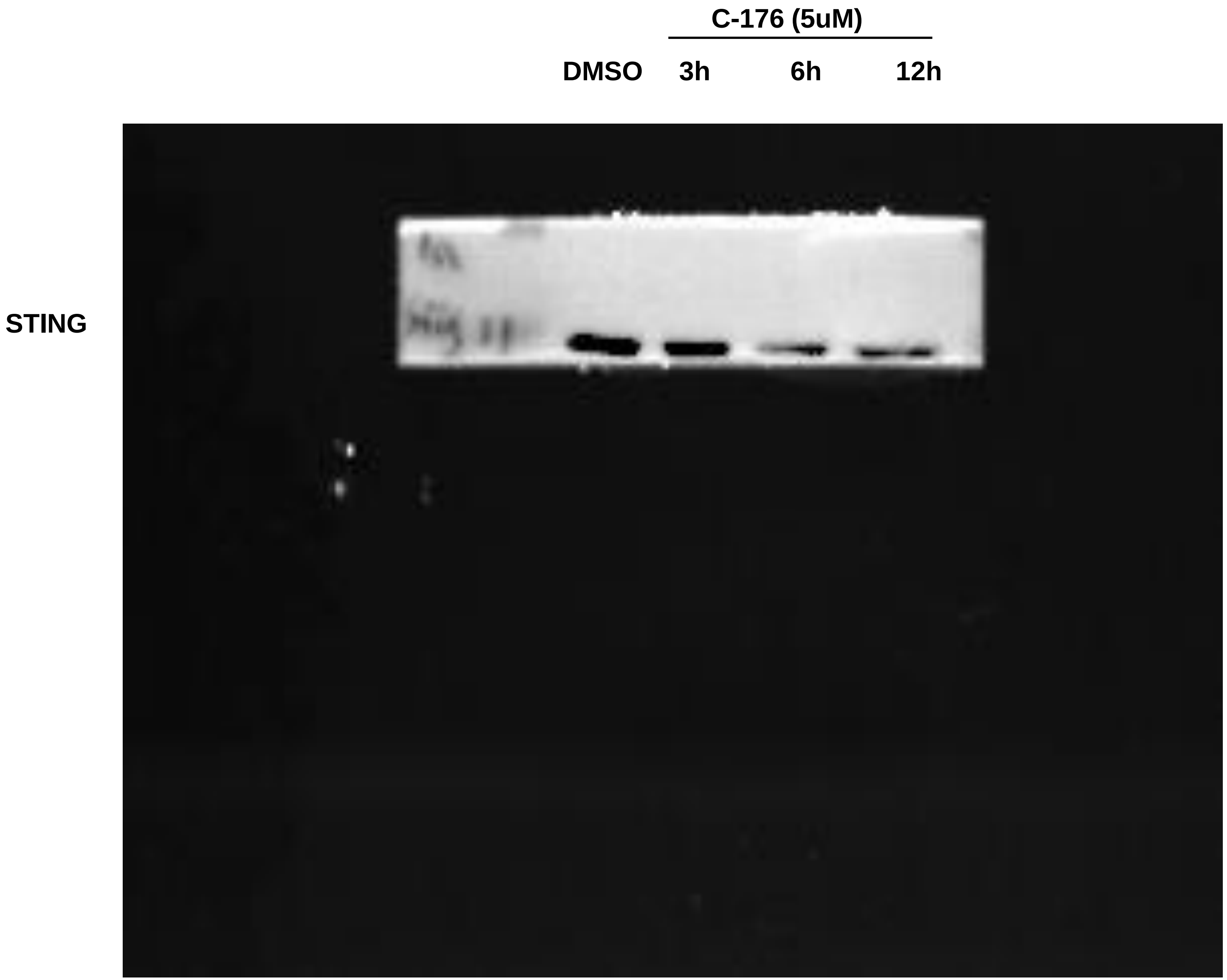

Figure 4D

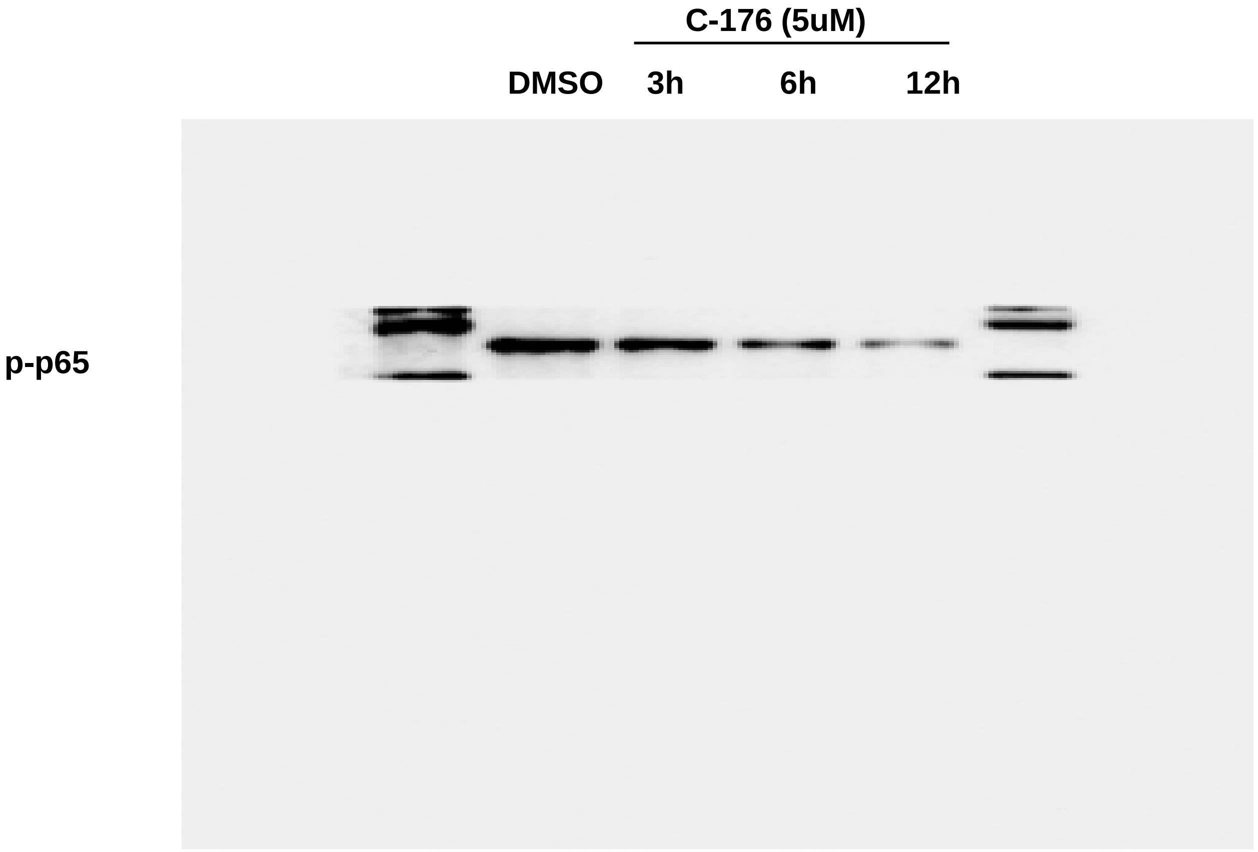

Figure 4D

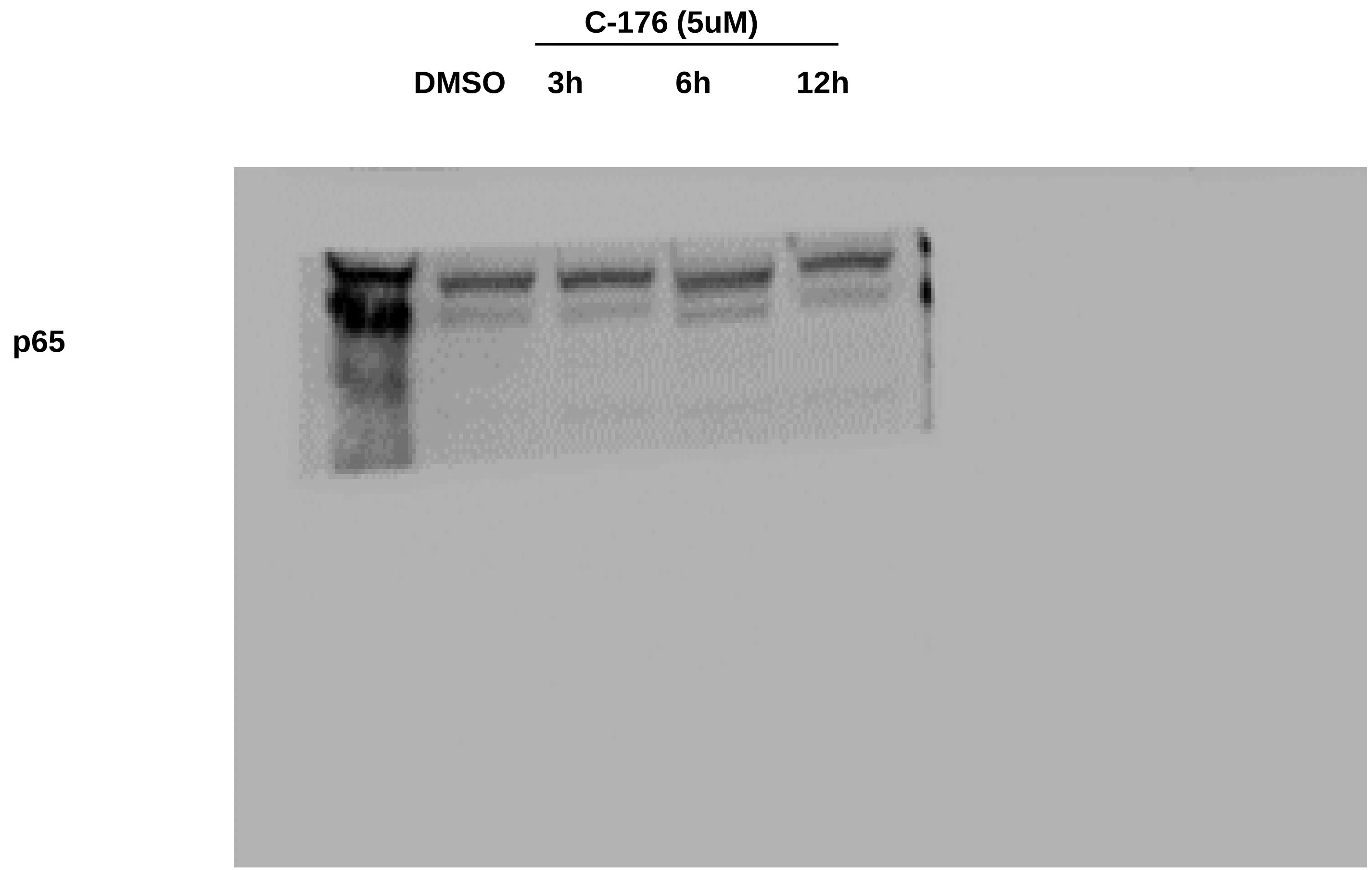

Figure 4D

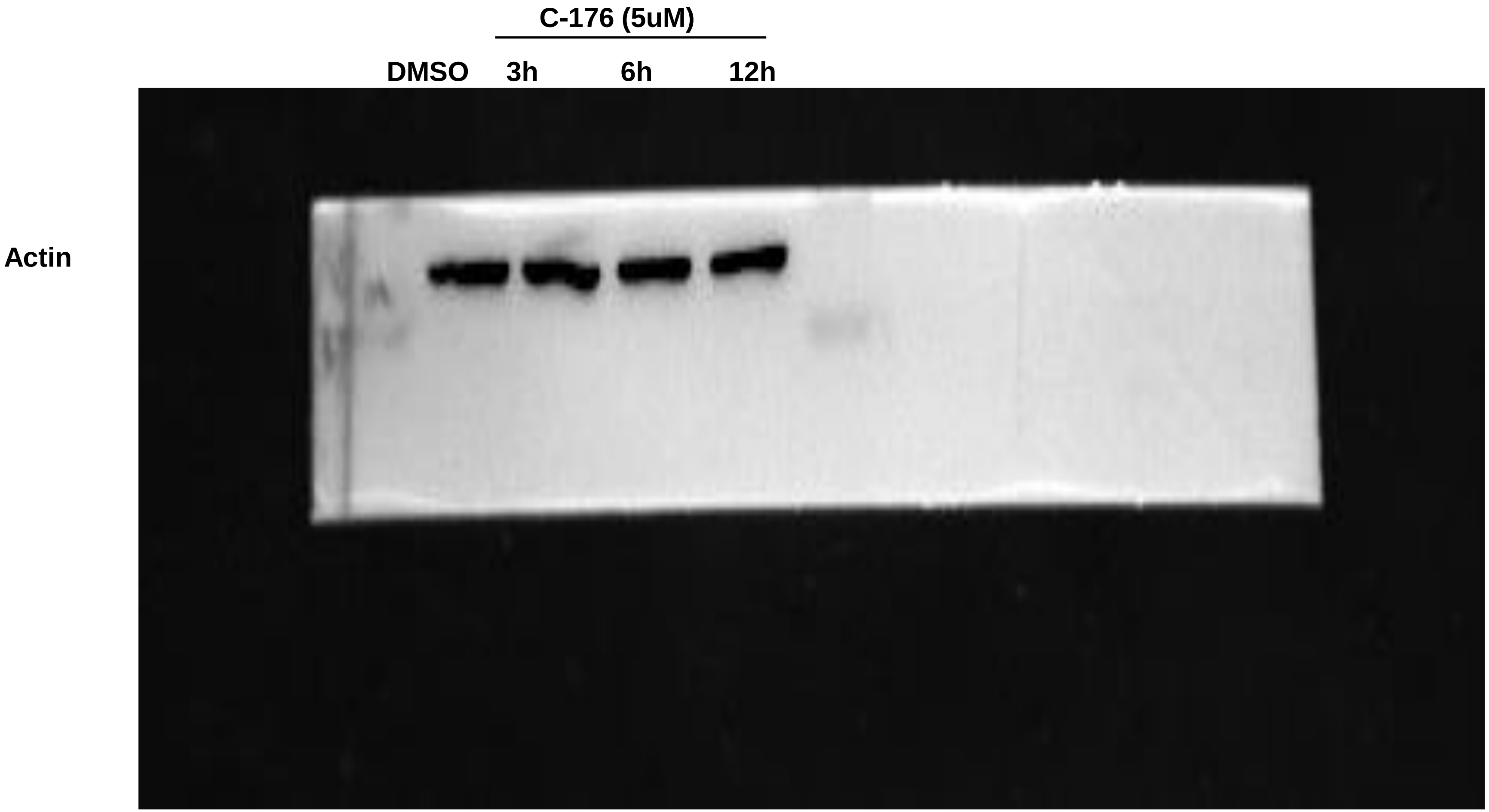

Figure 5A

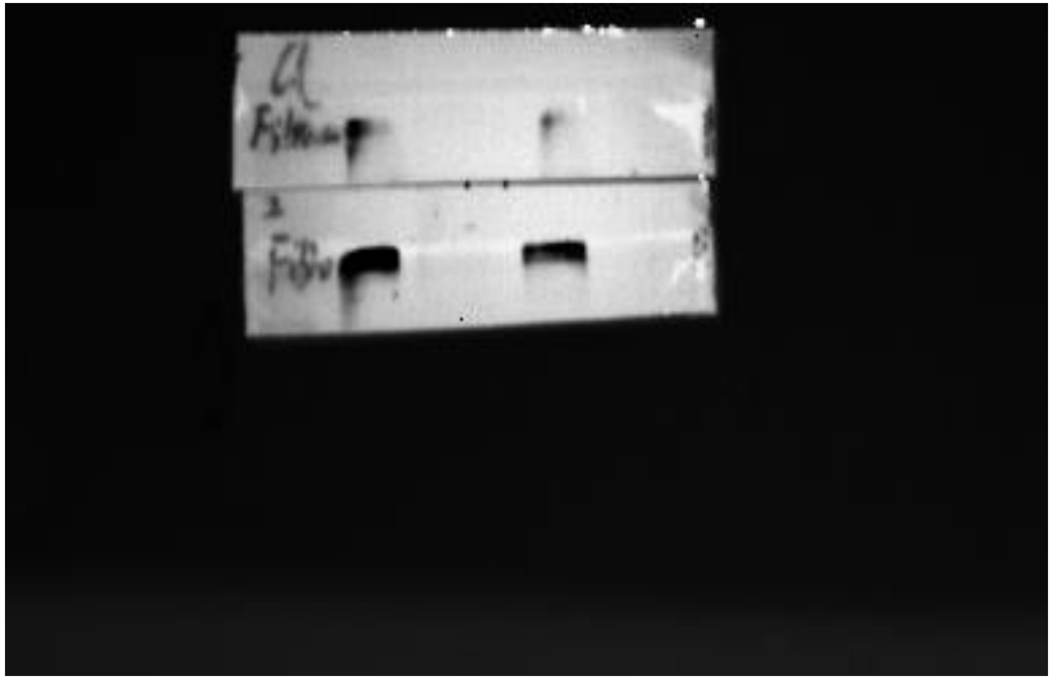

Figure 5A

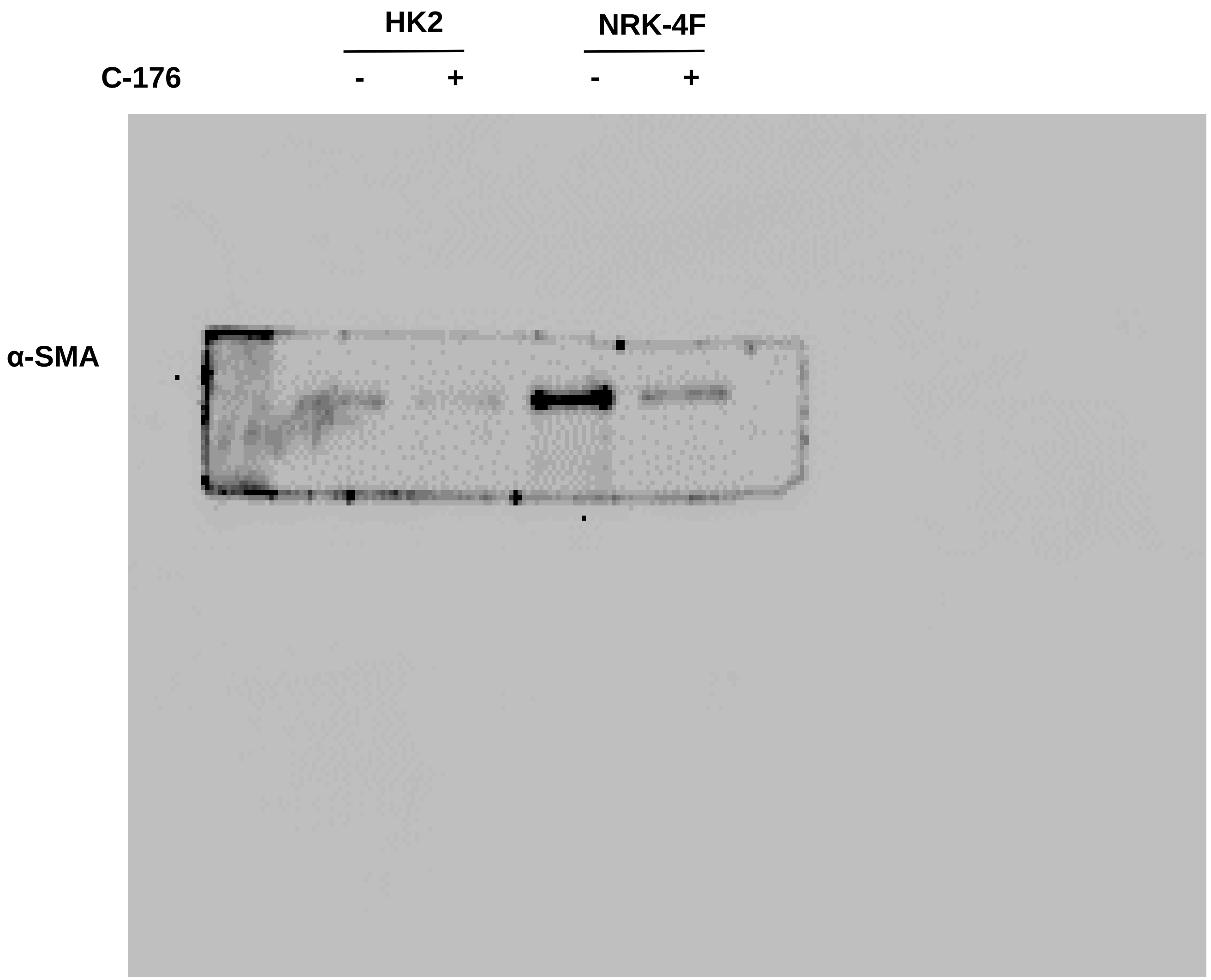

Figure 5A

HK2 NRK-4F

C-176 - + - +

Actin

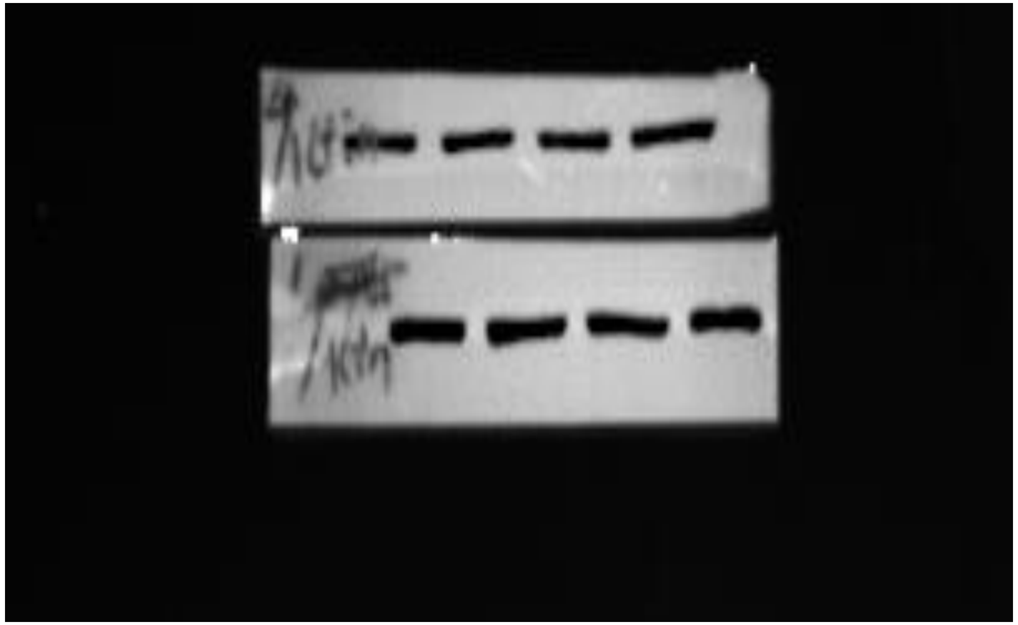

WT *Pkd1fl/fl: Pkhd1-Cre*

C-176 - - - - - - + + +

Fibronectin

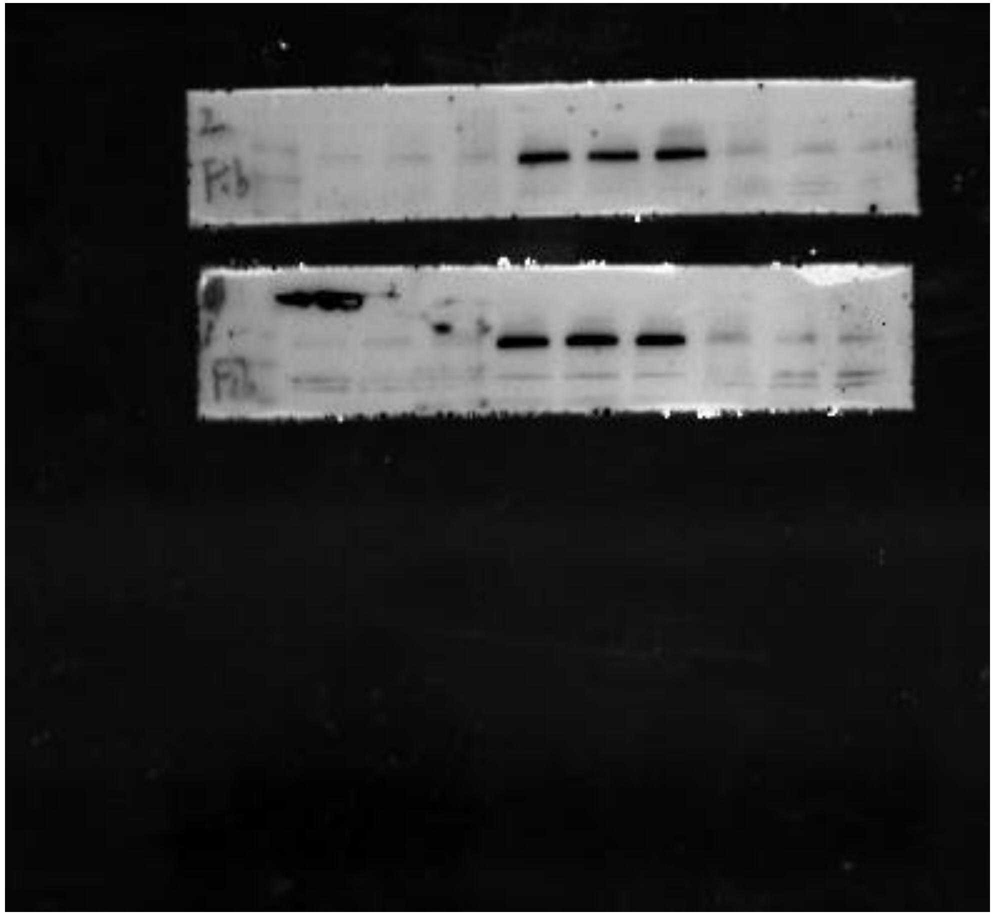

C-176 - - - - - - + + +

WT *Pkd1RC/RC*

Figure 5B

	WT			$Pkd1^{RC/RC}$					
C-176	-	-	-	-	-	-	+	+	+

α-SMA

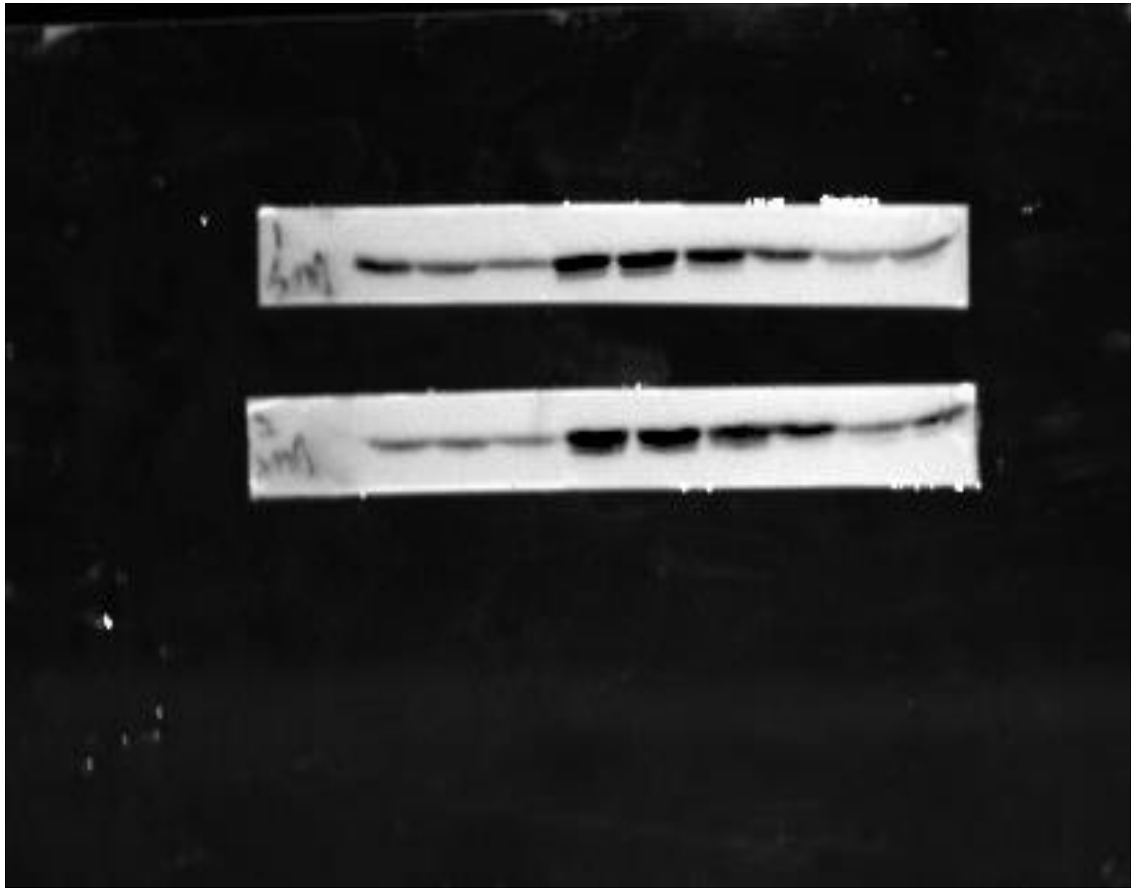

Figure 5B

WT | $Pkd1^{RC/RC}$

C-176 - - - - - - + + +

Actin

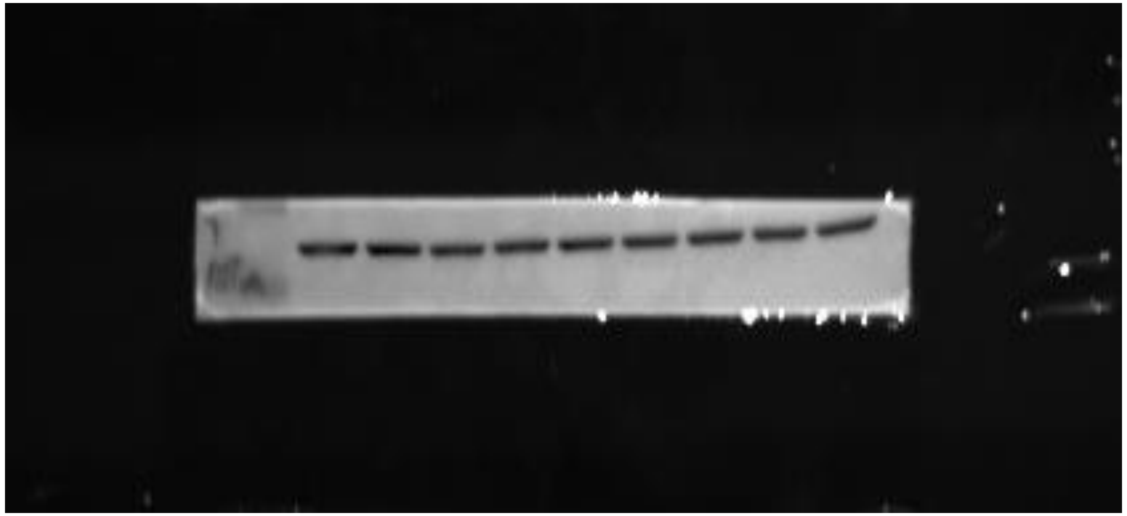

Figure 5C

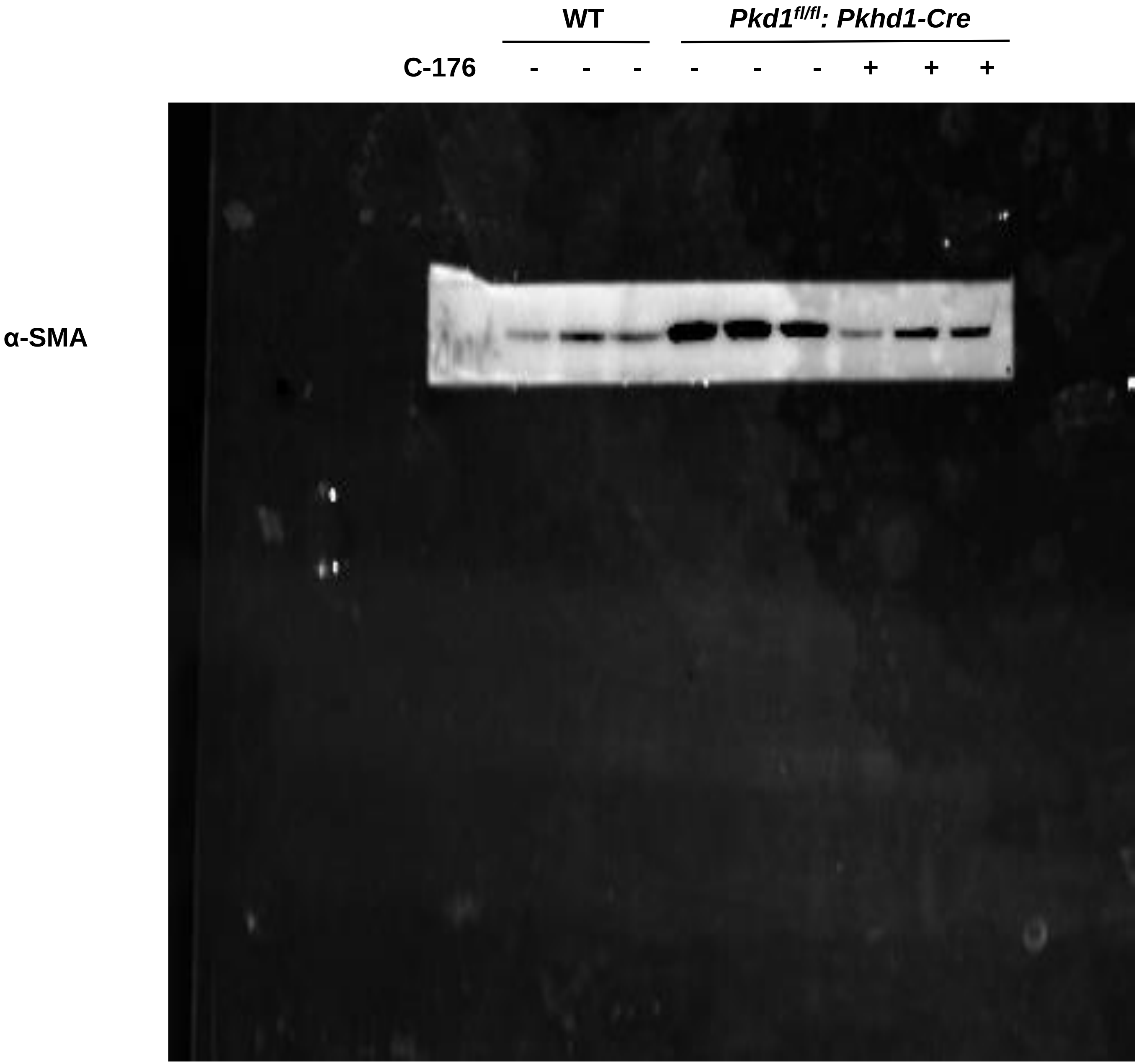

Figure 5C

	WT			*Pkd1$^{fl/fl}$: Pkhd1-Cre*					
C-176	-	-	-	-	-	-	+	+	+

Actin

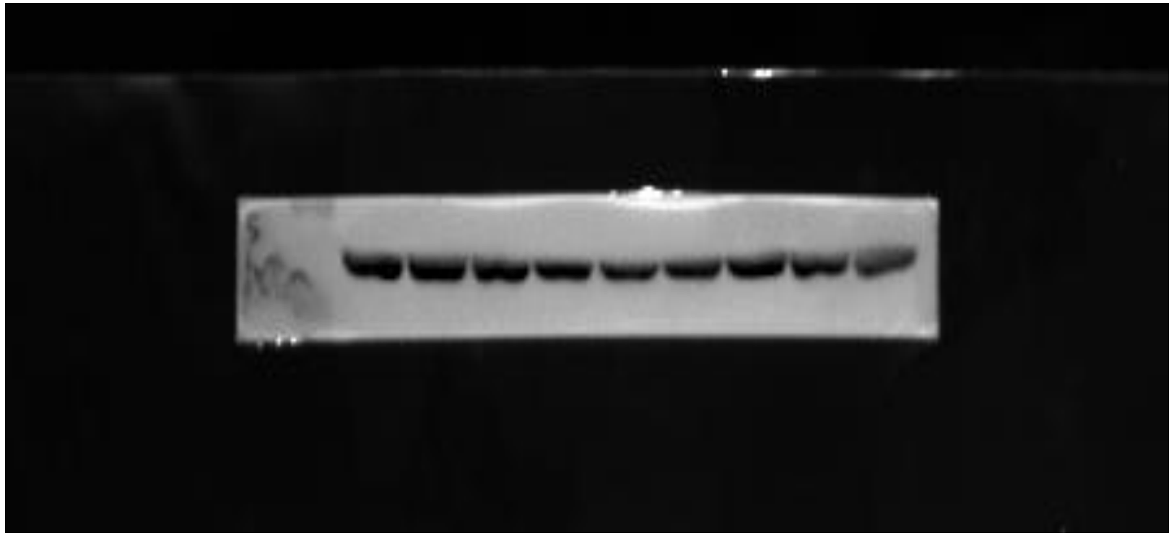

Figure 6C,D

Pkd1$^{RC/RC}$

C-176 - - - + + +

p53

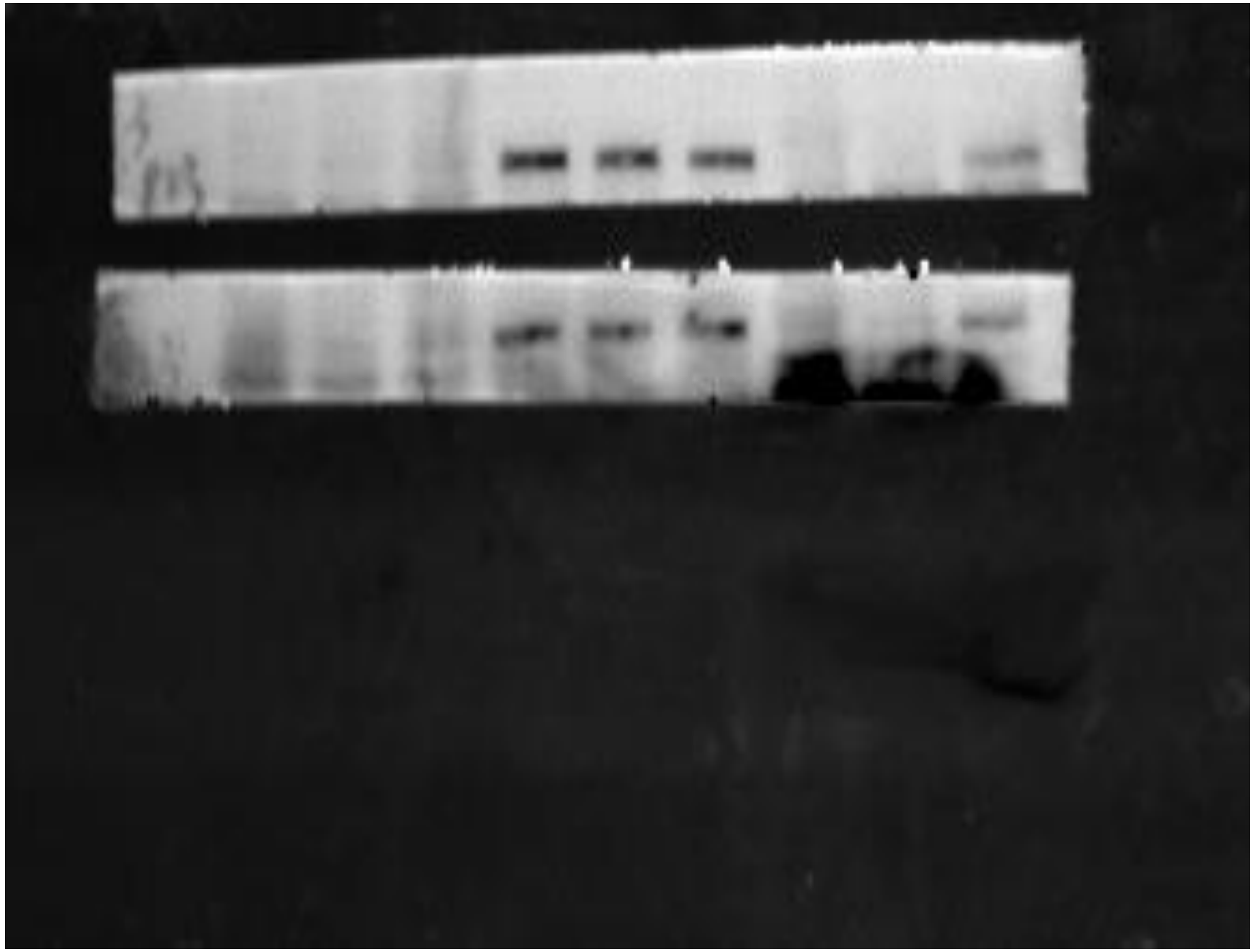

C-176 - - - + + +

Pkd1$^{fl/fl}$: Pkhd1-Cre

Figure 6C,D

*Pkd1^RC/RC^*

C-176 - - - + + +

Cleaved caspase3

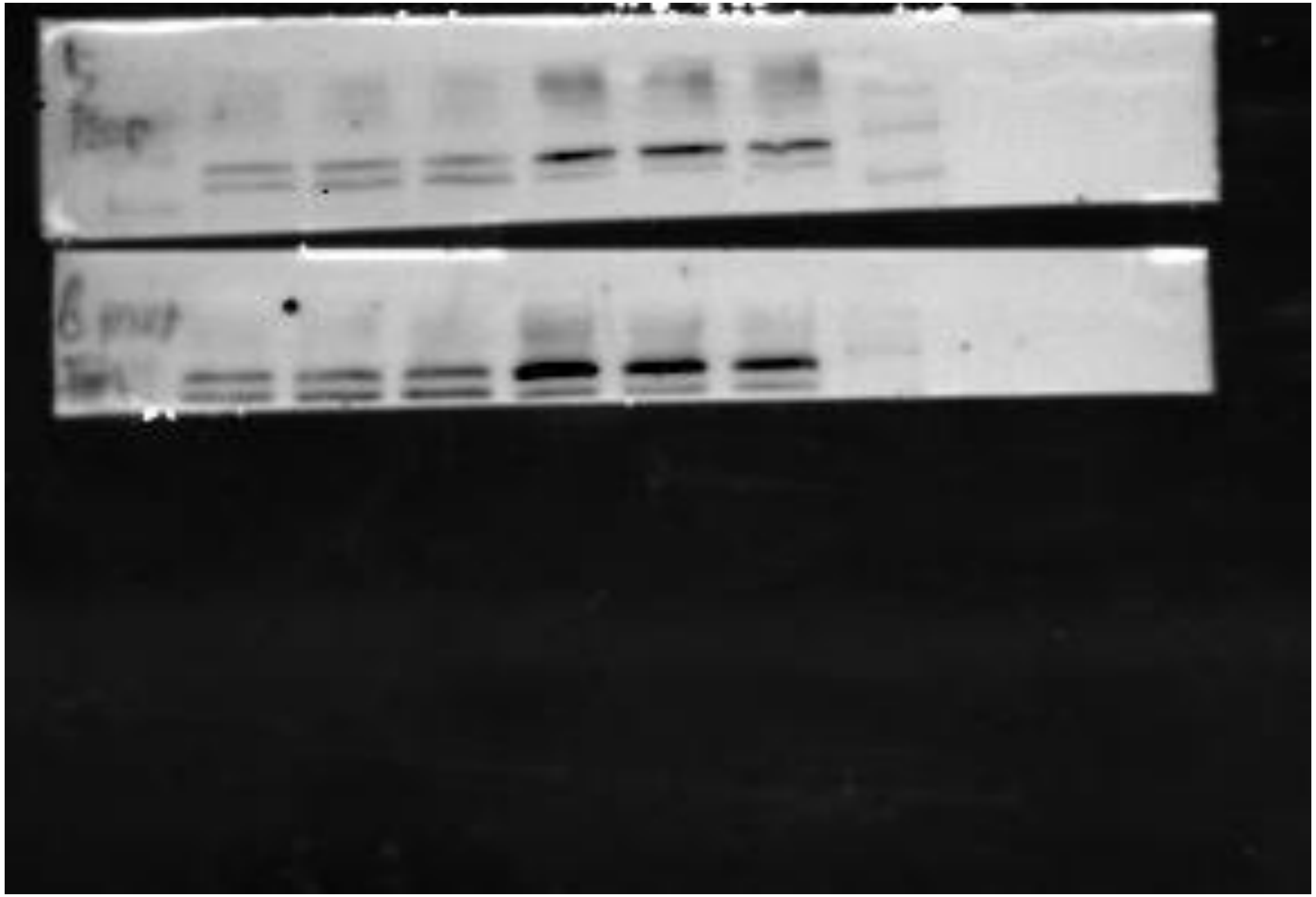

C-176 - - - + + +

Pkd1^fl/fl^: Pkhd1-Cre

Figure 6C

Pkd1$^{RC/RC}$

- - - + + +

Caspase3

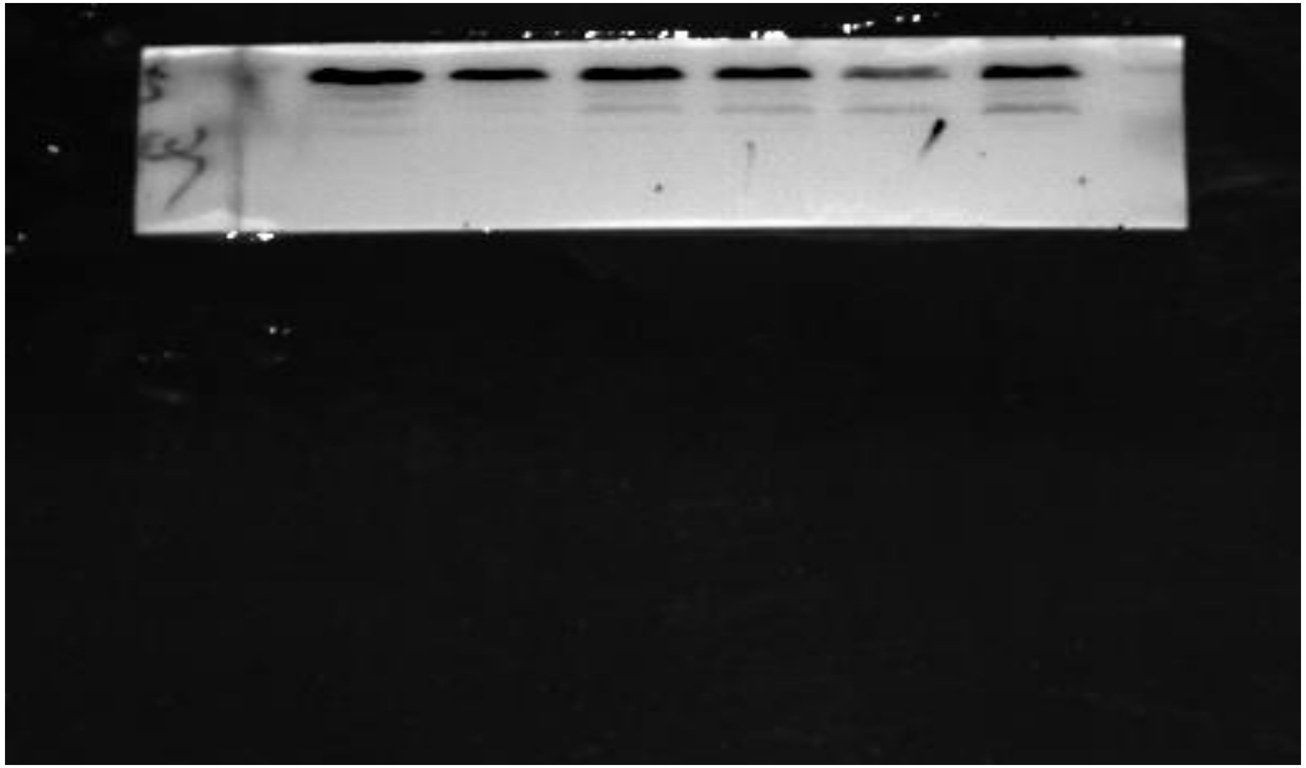

Figure 6D

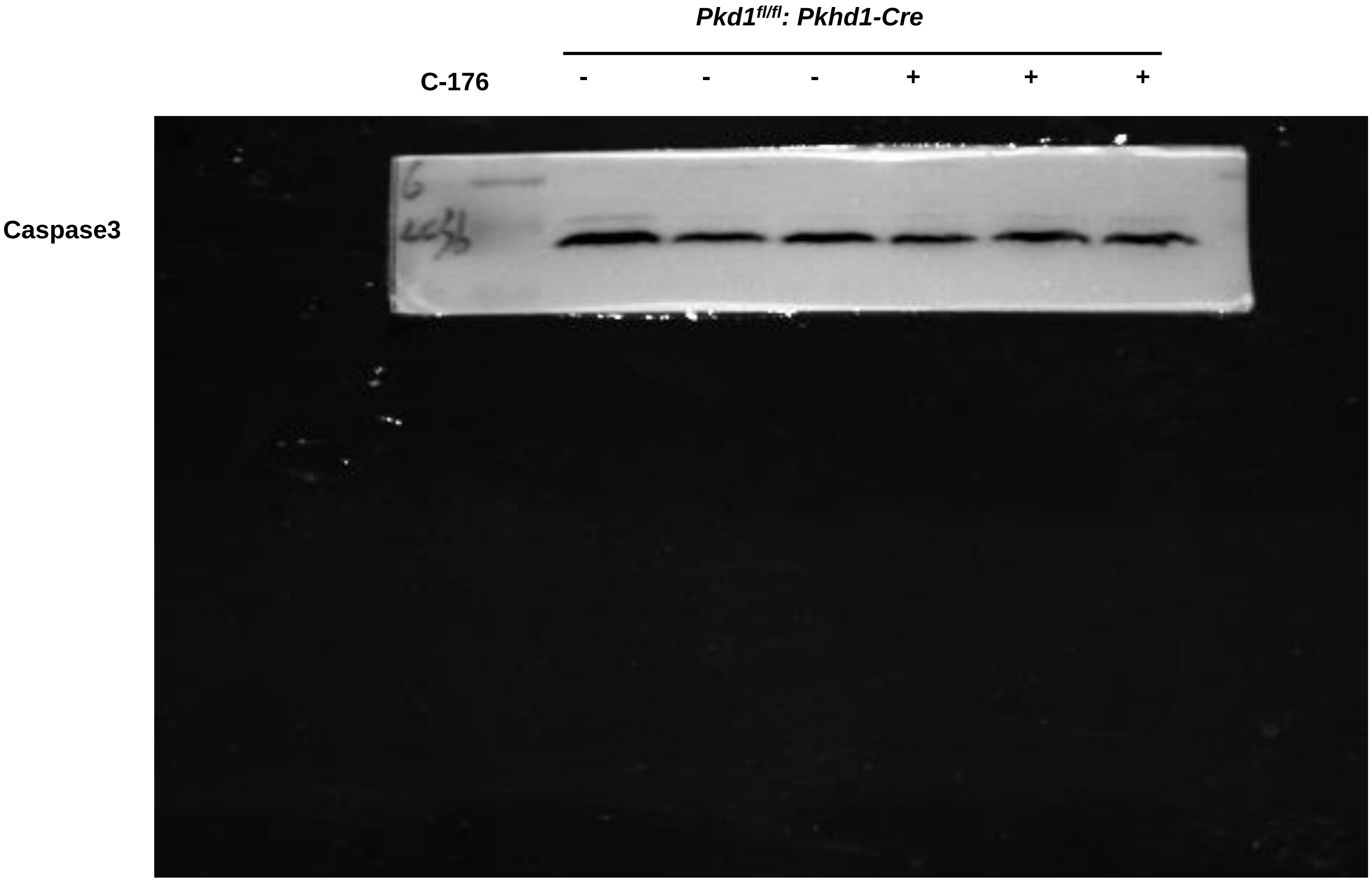

Figure 6C,D

Pkd1$^{RC/RC}$

C-176 - - - + + +

Actin

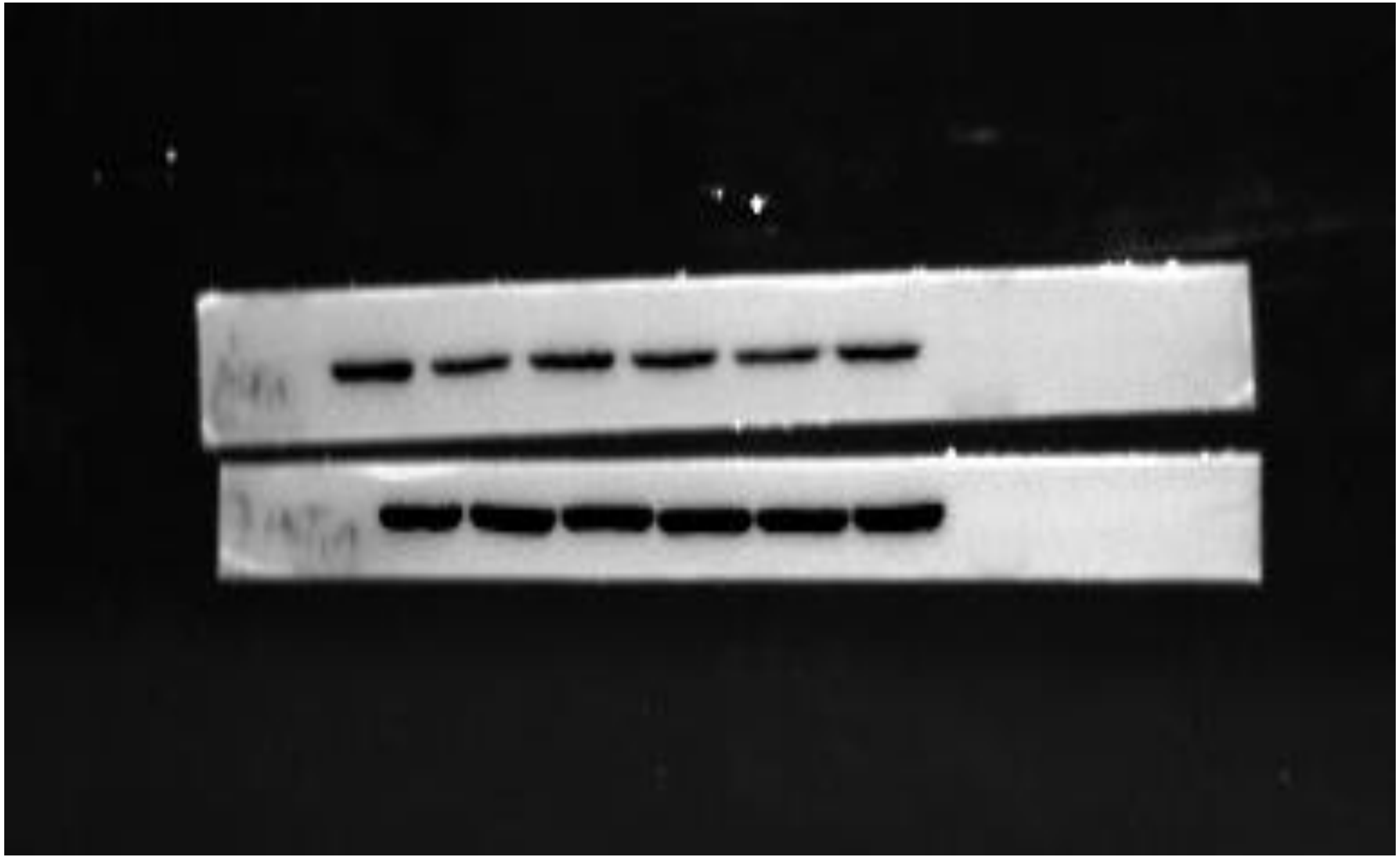

C-176 - - - + + +

Pkd1$^{fl/fl}$: Pkhd1-Cre

Figure 7C

p53 siRNA	-	+	-	+	-	+	-	+
C-176	-	-	+	+	-	-	+	+

p53

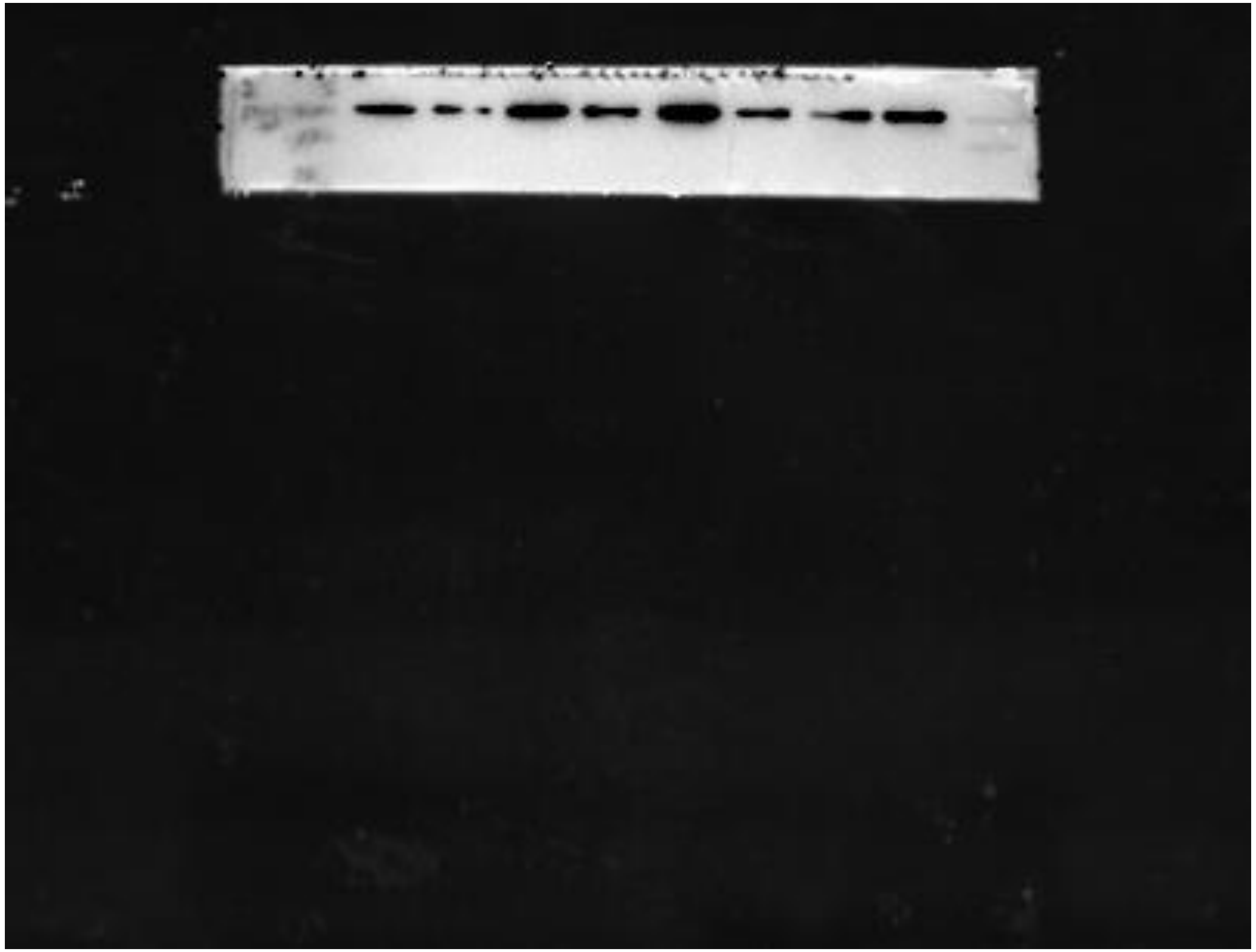

Figure 7C

p53 siRNA	-	+	-	+	-	+	-	+
C-176	-	-	+	+	-	-	+	+

Cleaved caspase3

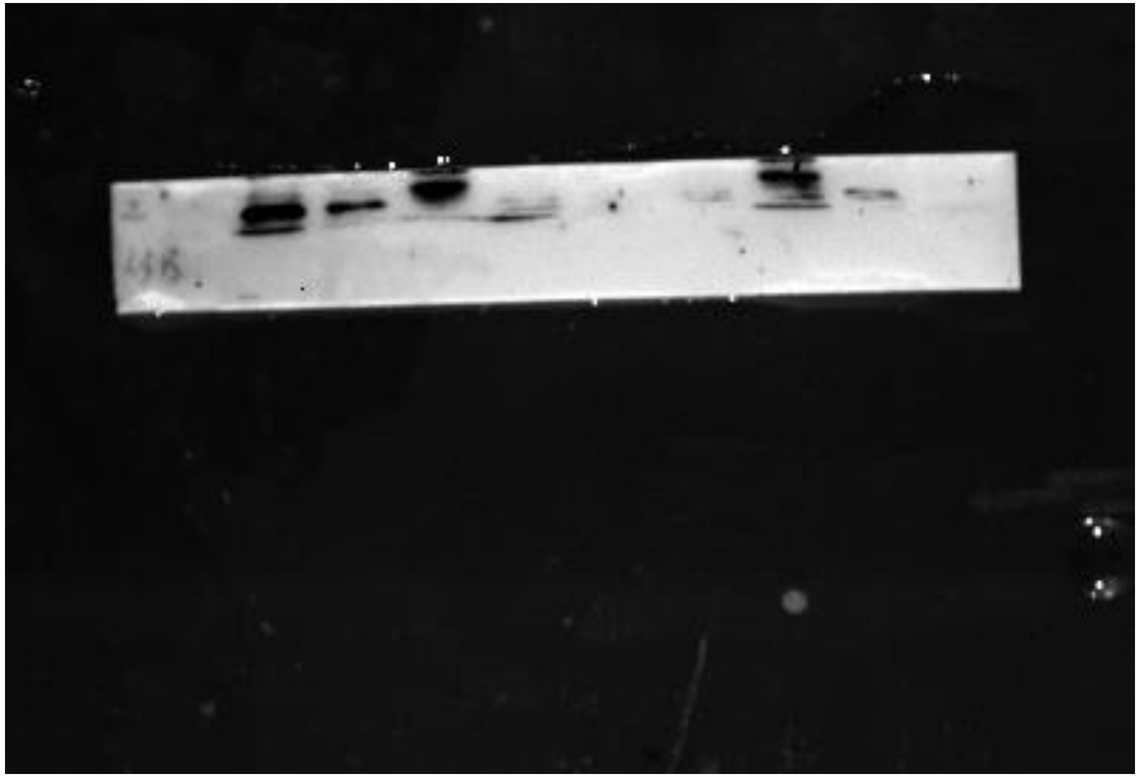

Figure 7C

p53 siRNA	-	+	-	+	-	+	-	+
C-176	-	-	+	+	-	-	+	+

Caspase3

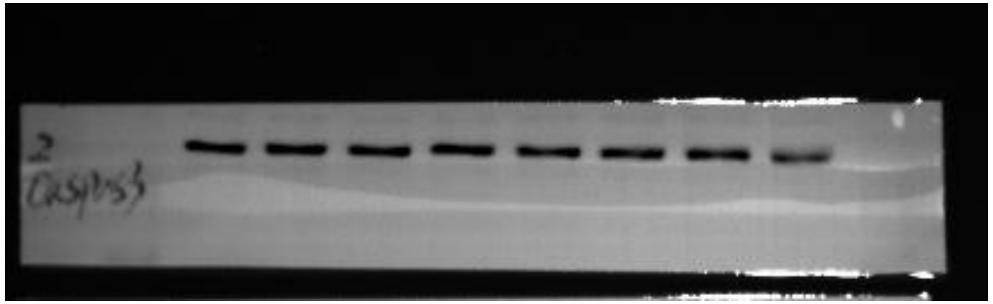

Figure 7C

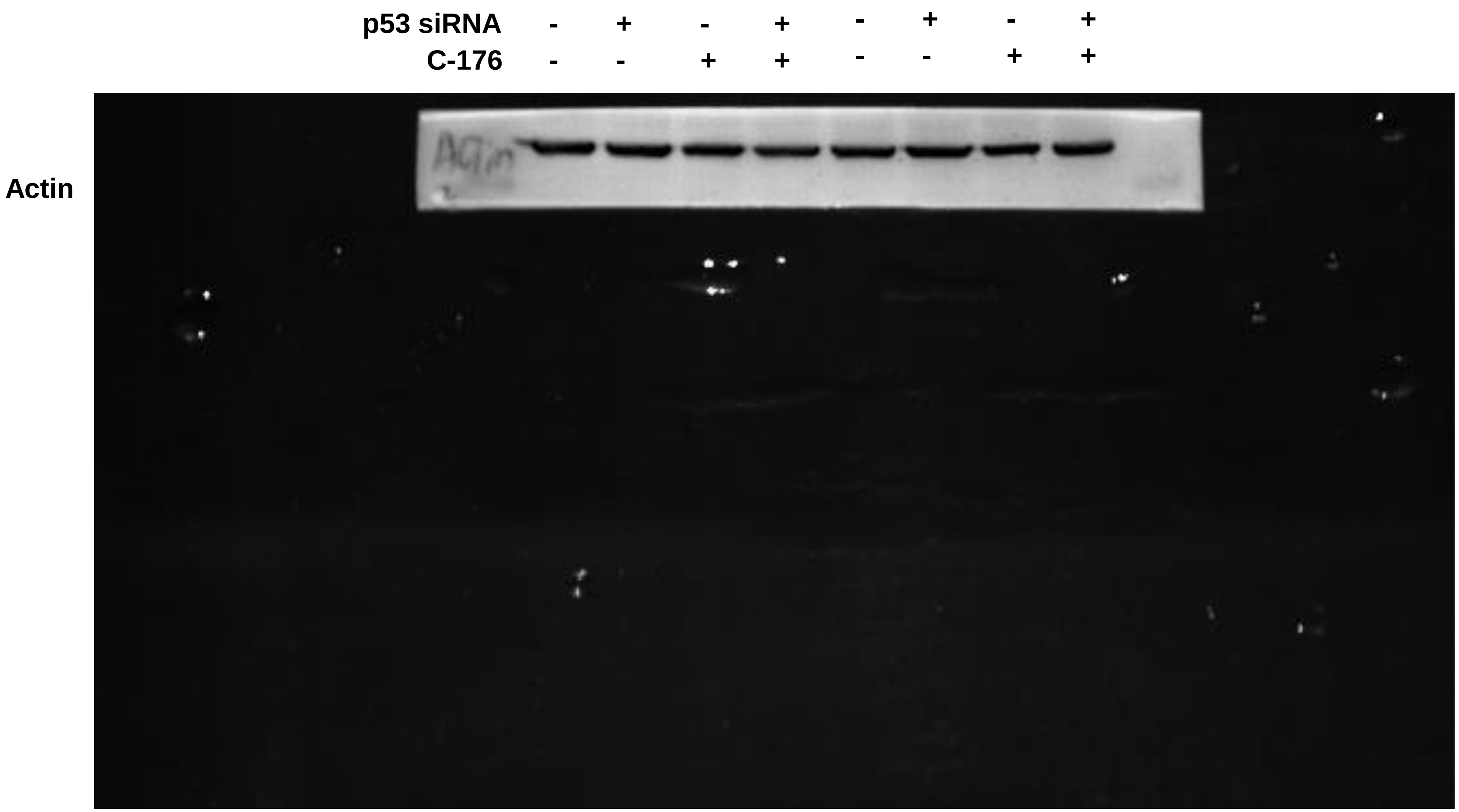

Supplement: Supplementary file 1 [file biomolecules-14-01215-s001.zip › biomolecules-3144628-supplementary-File S1.pdf]
