# Supplementary material for: STING Promotes the Progression of ADPKD by Regulating Mitochondrial Function, Inflammation, Fibrosis, and Apoptosis
Source: Biomolecules. 2024 Sep 26;14(10):1215. doi: 10.3390/biom14101215 (PMC11505933; doi:10.3390/biom14101215)
Supplement: Supplementary file 1 [file biomolecules-14-01215-s001.zip › biomolecules-3144628-supplementary-Table S1.pdf]

Supplementary Table S1. Primers used for quantitative real time PCR.

Gene name	Forward (5'-3')	Reverse (5'-3')
Actin	AAGAGCTATGAGCTGCCTGA	TACGGATGTCAACGTCACAC
Pkd1	CCCCGAATGTGGTTTCTATGG	GCCGTCCGATGTATGACTGC
a-SMA	GTCCCAGACATCAGGGAGTAA	TCGGATACTTCAGCGTCAGGA
TGF-β	CTGCTGACCCCCACTGATAC	AGCCCTGTATTCCGTCTCCT
Collagen1	GCTCCTCTTAGGGGCCAC	CCACGTCTCACCATTGGGG
TNF-a	ACCCTCACACTCAGATCATCTTC	TGGTGGTTTGCTACGACGT
MCP-1	TCTGGGCCTGCTGTTCACA	GGATCATCTTGCTGGTGAATGA
STING	GGAACACCGGTCTAGGAAGC	CAAGTGTCCGGCAGAAGAGT
mt-Nd6	TTAGCATTAAAGCCTTCACC	CCAACAAACCCACTAACAAT
mt-Co1	GCCCCAGATATAGCATTCCC	GTTCATCCTGTTCCTGCTCC
mt-Cytb	AGTAGACAAAGCCACCTTGA	CCGCGATAATAAATGGTAAG
mt-Rnr2	GTTACCCTAGGGATAACAGCGC	GATCCAACATCGAGGTCGTAAACC
